# Dynamics of DNA Damage Induced Pathways to Cancer

**DOI:** 10.1371/journal.pone.0072303

**Published:** 2013-09-04

**Authors:** Kun Tian, Ramkumar Rajendran, Manjula Doddananjaiah, Marija Krstic-Demonacos, Jean-Marc Schwartz

**Affiliations:** 1 Faculty of Life Sciences, University of Manchester, Manchester, United Kingdom; 2 School of Environment and Life Sciences, University of Salford, Salford, United Kingdom; 3 International Medical University, Kuala Lumpur, Malaysia; Semmelweis University, Hungary

## Abstract

Chemotherapy is commonly used in cancer treatments, however only 25% of cancers are responsive and a significant proportion develops resistance. The p53 tumour suppressor is crucial for cancer development and therapy, but has been less amenable to therapeutic applications due to the complexity of its action, reflected in 66,000 papers describing its function. Here we provide a systematic approach to integrate this information by constructing a large-scale logical model of the p53 interactome using extensive database and literature integration. The model contains 206 nodes representing genes or proteins, DNA damage input, apoptosis and cellular senescence outputs, connected by 738 logical interactions. Predictions from *in silico* knock-outs and steady state model analysis were validated using literature searches and *in vitro* based experiments. We identify an upregulation of Chk1, ATM and ATR pathways in p53 negative cells and 61 other predictions obtained by knockout tests mimicking mutations. The comparison of model simulations with microarray data demonstrated a significant rate of successful predictions ranging between 52% and 71% depending on the cancer type. Growth factors and receptors FGF2, IGF1R, PDGFRB and TGFA were identified as factors contributing selectively to the control of U2OS osteosarcoma and HCT116 colon cancer cell growth. In summary, we provide the proof of principle that this versatile and predictive model has vast potential for use in cancer treatment by identifying pathways in individual patients that contribute to tumour growth, defining a sub population of “high” responders and identification of shifts in pathways leading to chemotherapy resistance.

## Introduction

The p53 protein has been one of the most studied proteins since its discovery in 1979. It plays a central role in the regulation of cell survival and cancer development; p53 mutations are found in more than 50% of human tumours and alterations or lack of p53 function has been linked to most types of cancer cells. The p53 protein acts as a transcription factor, which regulates the expression of a large number of downstream genes by complex mechanisms [1]. It has anti-proliferative effects such as cell cycle arrest, apoptosis, and cell senescence in response to various stress signals. Moreover, p53 is a critical node of the cellular circuitry involved in the physiological response to growth factors or abnormal oncogenic stimuli. Post-translational modifications, protein-protein interactions and protein stabilization are found to be crucial levels of control of p53 activity.

However, despite its fundamental role p53 has been less amenable to therapeutic applications than other target genes or proteins that are successfully utilized in cancer treatments [2]. The understanding of p53 pathway mechanisms has both academic and commercial interest for the design of new cancer therapies and the selection of safer cancer drug candidates [3]. A major reason why it has been so difficult to exploit our knowledge of p53 for therapeutic applications is indeed the complexity of its action. There are more than 66,000 papers about p53 in the scientific literature, and yet we are still far from understanding the details of its function. This observation calls for a more systematic approach to integrate this vast amount of information into consistent representations that will enable better understanding of the systems-wide mechanisms regulating p53 function.

Network and systems biology approaches are offering promising new tools to study complex mechanisms involved in the development of diseases [4]. *In silico* models can integrate large sets of molecular interactions into consistent representations, amenable to systematic testing and predictive simulations. Models of various scales and computational complexity are being developed, from qualitative network representations to quantitative kinetic and stochastic models [5]–[7]. In the case of p53, the huge amount and complexity of molecular interactions involved makes a large-scale kinetic model out of reach. Nevertheless, a vast amount of biological knowledge is available on p53 that is not in the form of quantitative kinetic data, but in the form of qualitative information. For example, numerous reports indicated that ATM (ataxia telangiectasia mutated) affects p53 in response to DNA damage [8]. Although 1350 publications describe the link between ATM and p53 in PubMed, 57 papers indicate that ATM phosphorylates p53 and only 11 papers include the information that ATM phosphorylates and activates p53. Similarly, examples of downstream p53 target genes such as Bax (BCL2-associated X protein) that control the apoptosis process or CDKN1A (cyclin-dependent kinase inhibitor 1A (p21, Cip1)) that control cell cycle arrest are well studied [9], [10]. However, the detailed kinetics of only a subset of these interactions is known [11].

For this reason, we hypothesized that our understanding of p53 function can be enhanced by the systematic integration of such qualitative knowledge into a large-scale, consistent logical model. Unlike kinetic models, logical models do not use kinetic equations representing the detailed dynamic mechanism of each individual interaction, but unlike qualitative networks, they do incorporate information about the effects of interactions. This information is generally represented in the form of Boolean logic: each node (gene/protein) in the logical model can have two determined states, 0 or 1, representing an inactive or active form respectively; each interaction can have two determined effects, activation or inhibition of the target node. The advantages of logical models are that simulations are fast even for large models, they allow an extensive exploration of the space of node states with the identification of steady states or cycling attractors, and they provide an approximation of the actual nonlinear dynamics of the whole system. For example, Schlatter's group constructed a Boolean network based on literature searches and described the behaviour of both intrinsic and extrinsic apoptosis pathways in response to diverse stimuli. Their model revealed the importance of crosstalk and feedback loops in controlling apoptotic pathways [12]. Rodríguez et al. constructed a large Boolean network for the FA/BRCA (Fanconi Anemia/Breast Cancer) pathway and simulated the repair of DNA ICLs (interstrand cross-links). This model revealed the relationship between the activated DNA repair pathway and defects in the FA/BRCA pathway [13].

In this article, we present a logical model of the p53 system that integrates 203 genes/proteins, DNA damage input, apoptosis and cellular senescence outputs, connected by 738 logical interactions compiled from existing databases and the scientific literature. The model, hereafter named PKT206 (PKT standing for p53 model constructed by Kun Tian, and the number indicating the population of protein or gene nodes included in the model) can be used to predict effects of DNA damage pathways onto cellular fate. We present a functional analysis of this model and investigate the effects of knockouts using the CellNetAnalyzer software [14]. Several predictions produced by the model were validated from external literature and new experimental data, adding new contributions to our knowledge of the p53 system. The model's performance was tested using microarray analysis and we show that the ratio of good predictions substantially exceeds that of random predictions, ranging between 52% and 71%. It is found that the PKT206 model is a promising predictive tool that can increase our understanding of the complex mechanisms of p53 pathways and provides a novel approach to personalized cancer therapy.

## Results

### Model construction

**Figure 1:**
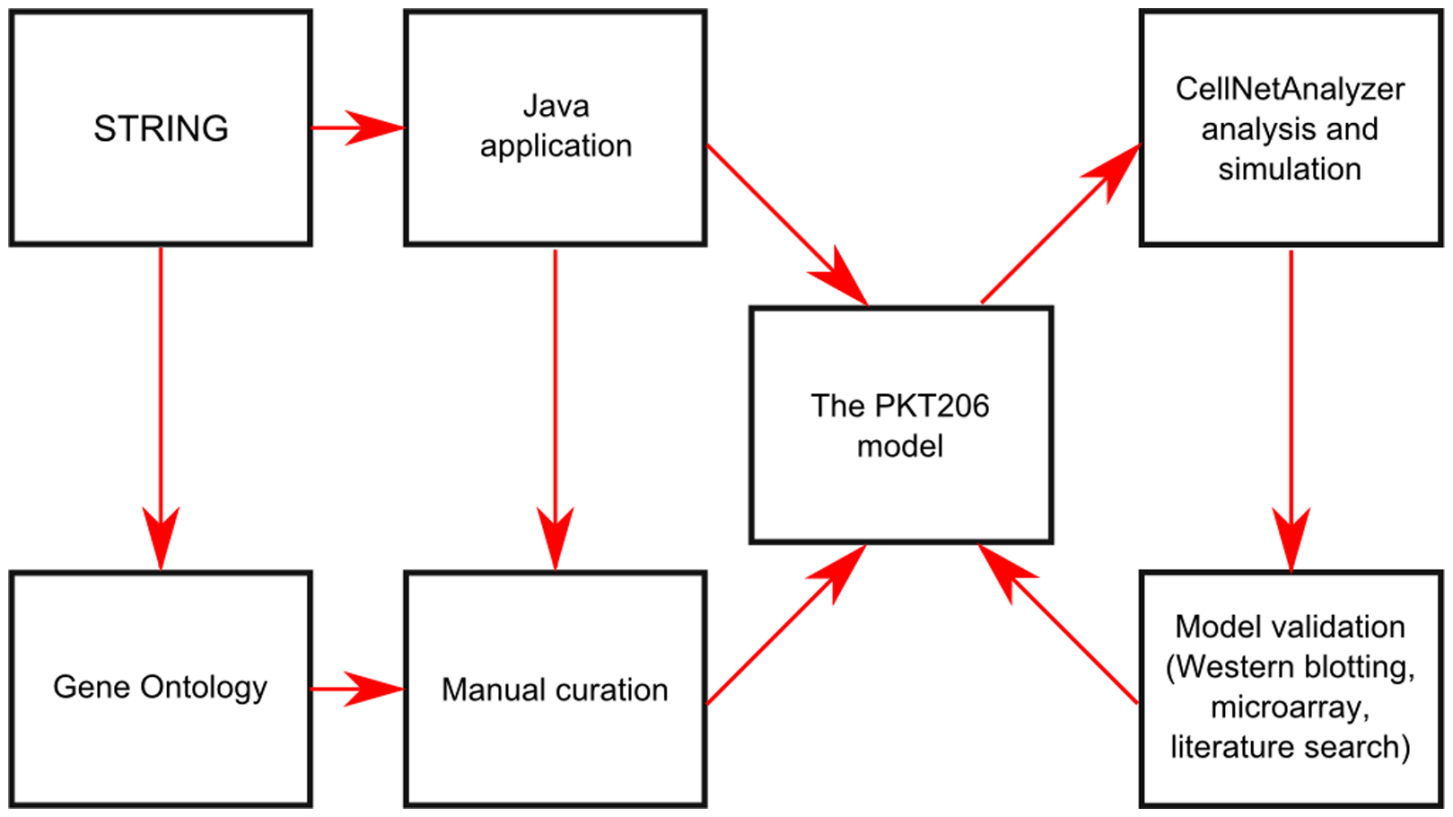
Flow chart of PKT206 logical model construction and analysis. Java interface programs were created to extract p53 interactions from the STRING database. We then manually curated the data and used Gene Ontology annotations to connect the network to DNA damage input and apoptosis output. CellNetAnalyzer was used for analysis and simulations, and the results were validated using literature surveys and experimental approaches including western blotting and microarray analysis.

In order to organize knowledge of the p53 interactome into a coherent framework, a logical model of the p53 system was constructed (Figure 1, Table S1 in File S1). In this model, nodes represent genes or associated proteins that interact with p53, and edges represent the interactions between them. Two types of interacting processes are considered: activating or inhibiting. In an activating interaction, the result is an induction of activity of target node(s), and in an inhibitory interaction, the result is a repression of activity of target node(s) [14]. For example, the induction of p53 stimulates the expression of MDM2 (Mdm2, p53 E3 ubiquitin protein ligase homolog (mouse)) [15], which is represented by an activating interaction from p53 to MDM2. At the same time, MDM2 activation leads to the down-regulation of p53, which is represented by an inhibiting interaction from MDM2 to p53 [16].

Although there are numerous databases recording genetic and protein-protein interactions, few record the effect the interaction has on the target node. A notable exception is the STRING (Search Tool for the Retrieval of Interacting Genes/Proteins) database [17], which distinguishes between different modes of action, including activation, inhibition and binding. Interaction records of the human p53 interactome were first retrieved automatically from the STRING database (see Material and Methods). The interactions were filtered by retaining only high confidence scores as defined by STRING (more than 0.7). However, because of the limitations of current text mining methods in identifying modes of action, even the group of high-confidence interactions was found to contain some errors. To avoid incorrect data being included into the model, all interaction records were thus manually curated by surveying the associated literature and searching for additional evidence. Examples of the types of errors found and details of interactions that were corrected following the manual curation process are provided in Table 2 and Figures S1–S4 in File S1.

**Table 2:** List of scenarios for the logical steady state analysis.

Scenario name	Input signal	Model type	Percentage of determined nodes
Scenario 1	DNA damage ON	P53 wild-type	87.9%
Scenario 2	DNA damage OFF	P53 wild-type	88.3%
Scenario 3	DNA damage ON	P53 knock-out	45.9%
Scenario 4	DNA damage OFF	P53 knock-out	46.3%

Four scenarios of logical steady state analysis with different input signals are defined with their input signal, model type and percentage of nodes having a determined state.

A recurrent question in the construction of *in silico* models is to define the boundaries of the system. In order to obtain a complete coverage of the p53 interactome, yet keep the size of the system within acceptable limits for simulation, we included all high-confidence interactions with genes/proteins interacting directly with p53, and added all interactions between these genes/proteins that do not involve p53 directly. This process ensured that regulatory feedback loops were included in the model. In a few cases, different proteins were combined into a single node reflecting the fact that earlier literature did not distinguish between them: this was the case for HRAS (v-Ha-ras, Harvey rat sarcoma viral oncogene homolog), KRAS (v-Ki-ras2, Kirsten rat sarcoma viral oncogene homolog), NRAS (neuroblastoma RAS viral (v-ras) oncogene homolog) and RASD1 (RAS, dexamethasone-induced 1), represented as a single node RAS; CCNA1 (cyclin A1) and CCNA2 (cyclin A2) represented as CCNA; CSNK2A1 (casein kinase 2, alpha 1 polypeptide) and CSNK2A2 (casein kinase 2, alpha prime polypeptide) represented as CSNK2.

Cells respond to numerous stress stimuli including ionizing and UV (ultraviolet) radiation, oncogene activation, heat shock, hypoxia, etc [18]. The DNA damage response mediated by p53 is well studied and most clinically relevant as the majority of cancer treatment strategies involve DNA damage pathways. Therefore, DNA damage was added as an input signal by connecting the network to a single input node representing DNA damage. Similarly, apoptosis and cellular senescence were selected as the best studied and most clinically relevant outputs among numerous other possibilities including cell cycle arrest, DNA repair and angiogenesis. Thus, the network was connected to two output nodes representing apoptosis and senescence. Links from DNA damage and towards apoptosis and senescence were curated using Gene Ontology terms (Tables S3–S5 in File S1) as well as additional manual curation. The resulting model, named PKT206, comprised 203 gene/protein nodes, an input node (DNA damage), two output nodes (apoptosis and senescence) and 738 interactions. Complete lists of genes/proteins and interactions with references to literature based evidence are provided in Tables S1 and S3–S5 in File S1.

### Structure of the p53 logical model

**Figure 2:**
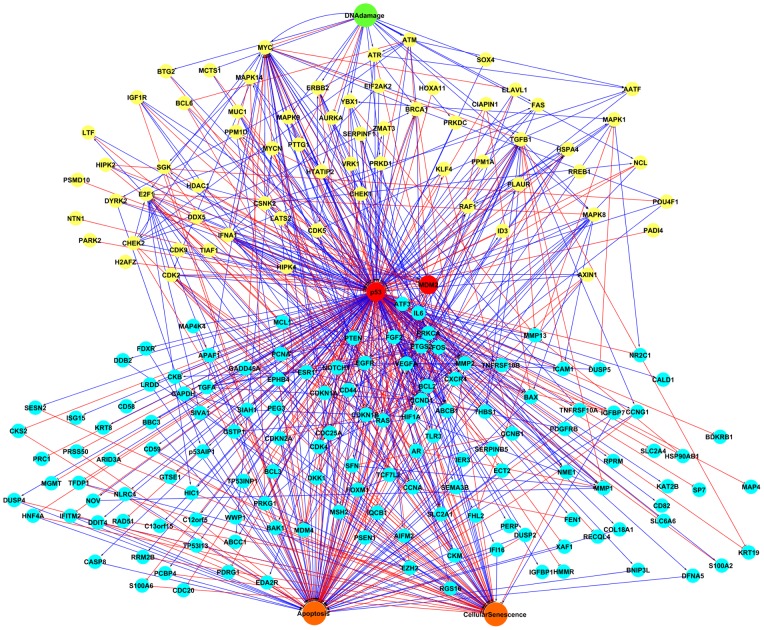
The PKT206 model. The PKT206 model represented by Cytoscape includes 203 gene/protein nodes, an input node (DNA damage), two output nodes (apoptosis and cellular senescence) and 738 edges. Activation and inhibition connections are represented by blue and red arrows, respectively. The input node was marked by green; the nodes upstream of p53 were marked by yellow; p53 and MDM2 were marked by red, the nodes downstream of p53 were marked by light blue and the output nodes were marked by orange.

The p53 node is connected to 202 genes or proteins in the network and participates in 225 interactions (Figure 2). Five layers can be distinguished in the network according to the relationship of nodes to p53: the input signal, DNA damage; upstream nodes of p53; p53 itself and MDM2; downstream nodes of p53; and the outputs, apoptosis and senescence. It was found that 67 nodes functioned as upstream nodes of p53. For example, ATM functions as a DNA damage inducible node upstream of p53 [19]; it activates p53 directly as well as through CHEK2 (checkpoint kinase 2) up-regulation [20]–[22]. 146 nodes functioned as p53 target genes, including well studied pro apoptotic genes such as BAX [9] and CDKN1A that controls cell cycle arrest [23]. 11 genes functioned both as upstream and downstream nodes of p53 and were involved in two step feedback loops.

**Figure 3:**
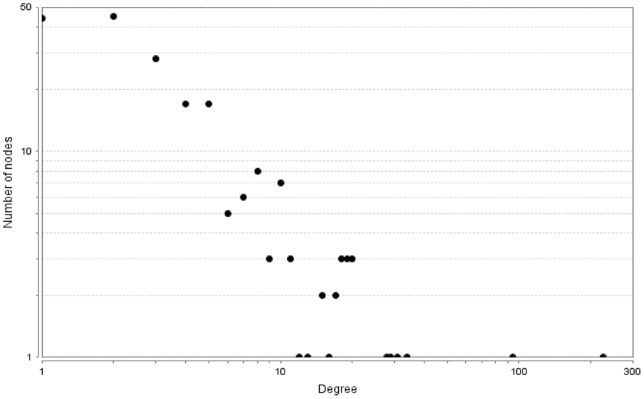
Connectivity degree distribution of 206 nodes. The degree distribution of 206 nodes in the model was obtained by the NetworkAnalyzer plugin for Cytoscape; both axes in the figure are in logarithmic scale.

We calculated the connectivity degree of the 206 nodes in the network (Figure 3). The connectivity degree of a gene indicates the number of interactions for this gene. The most connected gene was p53, which participated in 225 interactions in the PKT206 model. There were 30 genes with connectivity degree between 10 and 100 and the remaining genes were involved in less than 10 interactions.

The network contains 30 two-step feedback loops in total, with 14 involving p53. Some of them play a significant role in p53 regulation; for example, the feedback loops involving p53, MDM2 and MDM4 (Mdm4 p53 binding protein homolog (mouse)), which include five interactions: p53 activates MDM2; MDM2 inhibits p53; MDM2 inhibits MDM4; MDM4 activates MDM2 and MDM4 inhibits MDM2 [24]. Feedback loops play a crucial role in p53 regulation and are thought to increase the robustness of the system in response to perturbations [25].

P53 has been implicated in numerous cellular responses to stress including IR (ionizing radiation), UV, oncogene activation, and hypoxia. For this model to be able to predict cellular fate in response to stress, we linked 20 nodes to the input signal DNA damage (Table S3 in File S1). Most of the links from DNA damage are activations and only 3 are inhibitions (DNA damage inhibits PTTG1 (pituitary tumour-transforming 1), MYC (v-myc, myelocytomatosis viral oncogene homolog (avian)) and AURKA (aurora kinase A). Similarly, p53 controls numerous cellular responses to stress such as cell cycle arrest, DNA damage repair, senescence and apoptosis. We found 95 links between downstream gene nodes and apoptosis and 77 nodes interact with the apoptosis node. Among them, 18 nodes both promoted and prevented apoptosis, 38 nodes only induced apoptosis and 21 nodes only had anti-apoptotic function. We found 52 genes connected to senescence by 61 links, among which 28 promote and 33 prevent senescence.

### Analysis of dependencies in the p53 model

Logical dependencies between genes/proteins are represented by the dependency matrix [14], which represents the effects between all pairs of nodes in the model. Six types of effects are defined by CellNetAnalyzer based on the existence (or not) of positive and negative paths between two nodes: no effect, ambivalent factor, weak inhibitor, weak activator, strong inhibitor, and strong activator (see Methods for details). There are 42,436 (206×206) elements in the dependency matrix, of which 23,468 correspond to interactions having no effect; 16,540 are ambivalent factors; 1100 are weak inhibitors; 1240 are weak activators; 33 are strong inhibitors and 55 are strong activators (Table S6 in File S1). The majority of dependency matrix elements are no effect or ambivalent factors. The large number of ambivalent factors is due to the complexity of regulatory effects between nodes, which are affected by both positive and negative feedback loops and pathways. For example, there are both positive and negative paths from ATM to CHEK2: the positive path is a direct activation of CHEK2 by ATM, while the negative path is an indirect inhibition, as ATM activates p53, p53 inhibits MYC, MYC activates E2F1 (E2F transcription factor 1), and E2F1 activates CHEK2. As a result, the interaction between these two nodes is determined by opposing activating and inhibiting effects, resulting in it being classified as ambivalent (Figure S5 in File S1).

### *In silico* simulation of mutation effects

**Figure 4:**
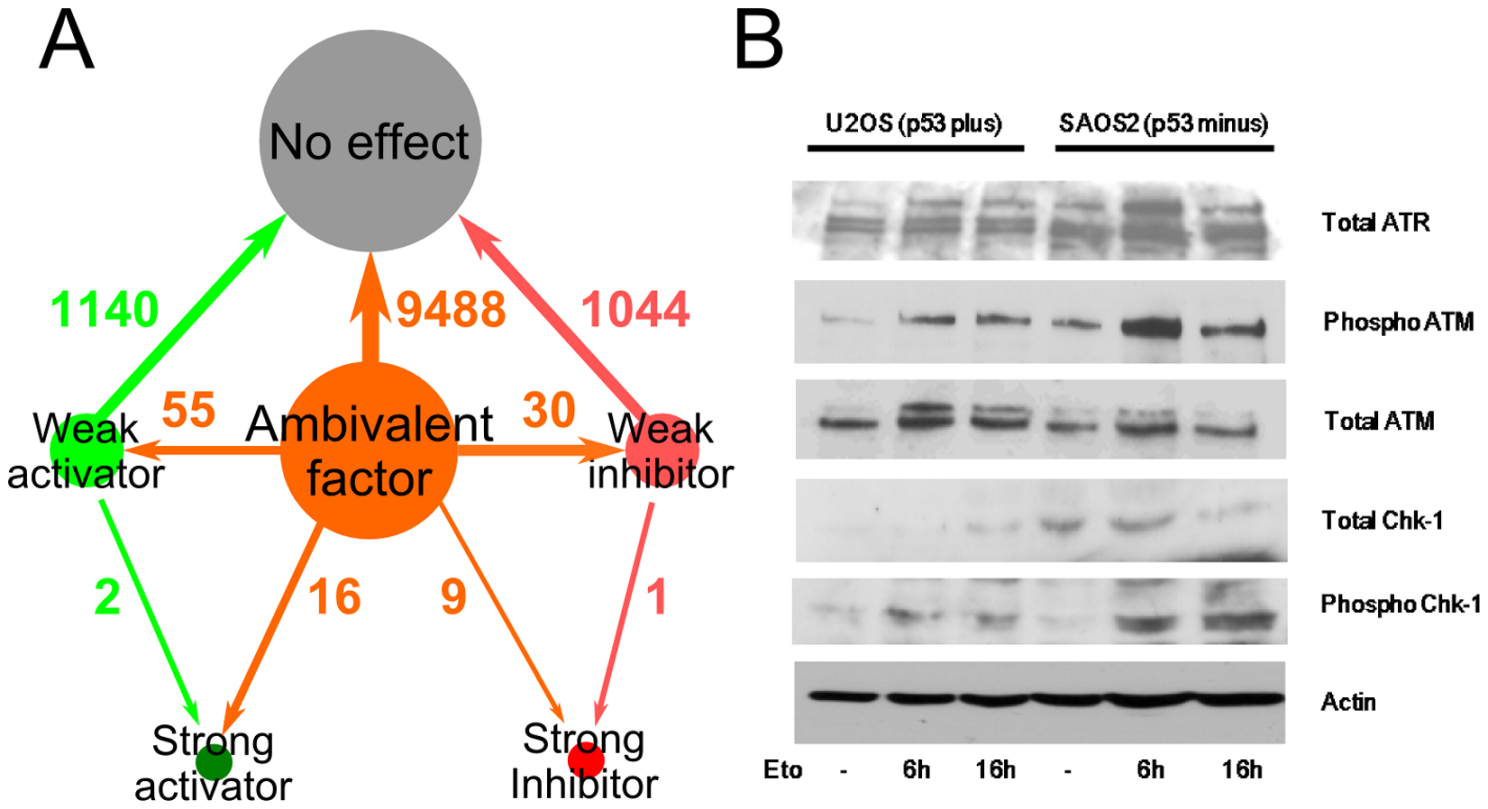
Validation of the PKT206 model. (A) Distribution of changes in the dependency matrix of the p53 *in silico* knock-out compared to the wild-type. The gray cycle represents no effect elements, the orange circle represents ambivalent factors, the light green circle represents weak activators, the pink circle represent weak inhibitors, the dark red circle represents strong inhibitors, and the dark green circle represents strong activators; the direction of the arrow represents the direction of changes in the knock-out. (B) Chk1 (CHEK1) activation is increased in p53 negative background. U2OS cells that have functional p53 and SAOS2 cells that lack functional p53 were treated with 10 µM etoposide for 16 hours. Cell extracts were analyzed by SDS PAGE and western blot analysis using antibodies against total Chk1, ATR and ATM. ATM and ATR phosphorylated Chk1 at Ser 345.

In order to evaluate the capacity of the PKT206 model to predict perturbation effects, we performed *in silico* knock-out tests, in which a particular node was removed from the network thus mimicking in vivo mutation effects. As 85% of genes or proteins in the PKT206 model were poorly connected, p53 and those 30 genes with more than 10 interactions were selected to perform *in silico* knock-out tests. For instance, we simulated a p53 knock-out by removing the p53 node from the network and analyzed the effects of this perturbation. By comparing the dependency matrix after the p53 node was removed with the wild-type case, changes in matrix elements revealed how relationships between nodes were affected by the deletion. 11,785 out of the 42,025 (205×205) elements in the matrix changed as a result of p53 removal (Figure 4A). Major changes are listed in Table S7 in File S1. The most significant changes were from ambivalent factors to activators or inhibitors, reflecting the fact that p53 plays a major role in modulating the system's effects. 11 out of 31 *in silico* knock-out tests had major changes in the new dependency matrix when a certain node was removed (Table S6 in File S1). 63 potential predictions of major changes in dependency cells were obtained from those 11 *in silico* knock-out tests (Table 1). There were no major effect changes found in the other 20 *in silico* knock-out tests.

**Table 1:** Validations of model predictions in the *in silico* knock-out tests.

Gene deleted	Activated node	Reported effects from literature	References	Predictions	Verified status
p53	DNA damage	Expression level of Fas enhanced	[26]	DNA damage promoted upregulation of FAS	Verified by literature
p53	LATS2	Cell death enhanced	[27]	LATS2 induced apoptosis	Verified by literature
p53	DNA damage	Expression level of CHEK1 enhanced	[28]	DNA damage promoted upregulation of CHEK1	Verified by literature
P53	KLF4	CCNB1 reduced	[29]	KLF4 reduced expression of CCNB1	Verified by literature
P53	ATM			ATM enhanced CHEK1	Verified in this publication
P53	ATR			ATR enhanced CHEK1	Verified in this publication
P53	MAPK14	Stimulation of BAX	[59]	BAX enhanced	Consistent with prediction
VEGFA	SERPINB5	Apoptosis enhanced in the presence of MMP3 and MMP9 inhibition	[60]	Apoptosis enhanced	Consistent with prediction
MDM2	ATM	DYRK2 induced in the presence and absence of DNA damage	[61]	DYRK2 enhanced	Consistent with prediction
MDM2	ATR	DYRK2 induced in the presence and absence of DNA damage	[61]	DYRK2 enhanced	Consistent with prediction
CDK2	CDKN1A	Apoptosis decreased but not confirmed directly	[62], [63]	Apoptosis reduced	Consistent with prediction
CDK2	CDKN1A	CDK2 regulates senescence suppression by MYC	[55]	Cellular senescence increased	Consistent with prediction
P53	SGK			Cellular senescence decreased	PNP
P53	MAPK14			Cellular senescence decreased	PNP
P53	LATS2			Cellular senescence decreased	PNP
VEGFA	FOXM1			Cellular senescence decreased	PNP
P53	IFNA1			CDK4 reduced	PNP
P53	IFNA1			FGF2 reduced	PNP
P53	PPM1D			CHEK1 reduced	PNP
P53	SFN			CCNB1 reduced	PNP
P53	DNA damage			CDK4 reduced	PNP
P53	DNA damage			FGF2 reduced	PNP
P53	FGF2			CDK4 enhanced	PNP
P53	FOXM1			CCNB1 enhanced	PNP
P53	FAS			Apoptosis enhanced	PNP
P53	PTTG1			CDK4 enhanced	PNP
P53	PTTG1			FGF2 enhanced	PNP
P53	IFNA1			FAS enhanced	PNP
P53	DYRK2			P53AIP1 enhanced	PNP
P53	DYRK2			Apoptosis enhanced	PNP
P53	MAPK14			MMP2 enhanced	PNP
P53	MAPK14			SGK enhanced	PNP
MYC	TCF7L2			Apoptosis reduced	PNP
VEGFA	TLR3			CXCR4 reduced	PNP
VEGFA	TLR3			TNFRSF10B reduced	PNP
VEGFA	CXCR4			TNFRSF10B enhanced	PNP
VEGFA	CXCR4			Apoptosis enhanced	PNP
VEGFA	FOXM1			MMP2 enhanced	PNP
VEGFA	FOXM1			BAX enhanced	PNP
VEGFA	FOXM1			CCNB1 enhanced	PNP
VEGFA	FOXM1			Apoptosis enhanced	PNP
CCND1	PDGFRB			Apoptosis reduced	PNP
TGFB1	DKK1			Apoptosis reduced	PNP
TGFB1	DNA damage			MAPK8 enhanced	PNP
E2F1	AATF			CDK5 enhanced	PNP
E2F1	CHEK2			AATF enhanced	PNP
E2F1	CHEK2			CDK5 enhanced	PNP
E2F1	CSNK2			MYCN enhanced	PNP
E2F1	ATM			AATF enhanced	PNP
E2F1	ATM			CHEK2 enhanced	PNP
E2F1	ATM			CDK5 enhanced	PNP
E2F1	ATR			AATF enhanced	PNP
E2F1	ATR			CDK5 enhanced	PNP
E2F1	DNA damage			AATF enhanced	PNP
E2F1	DNA damage			CHEK2 enhanced	PNP
E2F1	DNA damage			CDK5 enhanced	PNP
EGFR	BCL3			Apoptosis reduced	PNP
HIF1A	GAPDH			SIAH1 enhanced	PNP
HIF1A	GAPDH			Apoptosis enhanced	PNP
HIF1A	SIAH1			Apoptosis enhanced	PNP
CXCR4	TLR3			Apoptosis enhanced	PNP
P53	IFNA1	TLR3 reduced	[30]	TLR3 enhanced	Opposite to prediction

This table lists 63 predictions in the selected gene deletion background. Some predictions were verified by existing literature survey or experimental approaches. Potential novel predictions (PNP) indicate that we were unable to identify literature based evidence relevant to the prediction.

We confirmed 4 out of these 63 predictions through literature searches, focusing on major changes caused by the p53 deletion which were expected to have stronger experimental effects. For example, the effect of DNA damage onto FAS (Fas (TNF receptor superfamily, member 6)) changed from an ambivalent factor in the p53 wild-type model to a strong activator when p53 was removed. The effect of DNA damage onto FAS was classified as ambivalent in the wild-type cells because there are potential negative paths from DNA damage to FAS through MYC and PTTG1, in addition to a direct positive path from DNA damage to FAS. When p53 is deleted, only the positive path subsists. Manna et al. have determined that in p53 minus cells, Fas protein levels are elevated under DNA damage compared to p53 wild-type cells, which is in agreement with our prediction [26]. Similarly to FAS, the effect of LATS2 (LATS, large tumour suppressor, homolog 2 (Drosophila)) onto apoptosis was changed from an ambivalent factor in the p53 wild-type model to a strong activator when p53 was removed. It was found that in both p53 wild-type (A549) and p53 minus cells (H1299), LATS2 was able to induce apoptosis and that apoptosis is slightly increased in H1299 as measured by PARP and caspase 9 cleavage [27]. We observed that the effect of DNA damage onto CHEK1 (checkpoint kinase 1) changed from an ambivalent factor in the p53 wild-type to a strong activator when p53 was removed. CHEK1 protein levels were found to be higher in p53 −/− cells than in p53 +/+ HCT116 colorectal cancer cells treated by daunorubicin [28], which also matches our predictions (Table 1). It was reported that KLF4 (Kruppel-like factor 4(gut)) caused more reduction of CCNB1 (cyclin B1) expression in p53 −/− HCT116 than in p53 +/+ HCT116 cells [29] and it matched our model prediction. However, one prediction out of those 63 predictions was found opposite to the literature evidence. The prediction pointed out that IFNA1 (interferon, alpha 1) enhanced TLR3 (toll-like receptor 3) in p53 mutant cells compared to p53 wild type cells. But this was opposite to the fact reported by Taura et al. that IFNA1 exposed to the DNA damaging drug 5-fluoro-uracil(5-FU) reduced the expression of TLR3 in p53 −/− HCT116 cell compared to p53 +/+ HCT116 cells [30].

**Figure 5:**
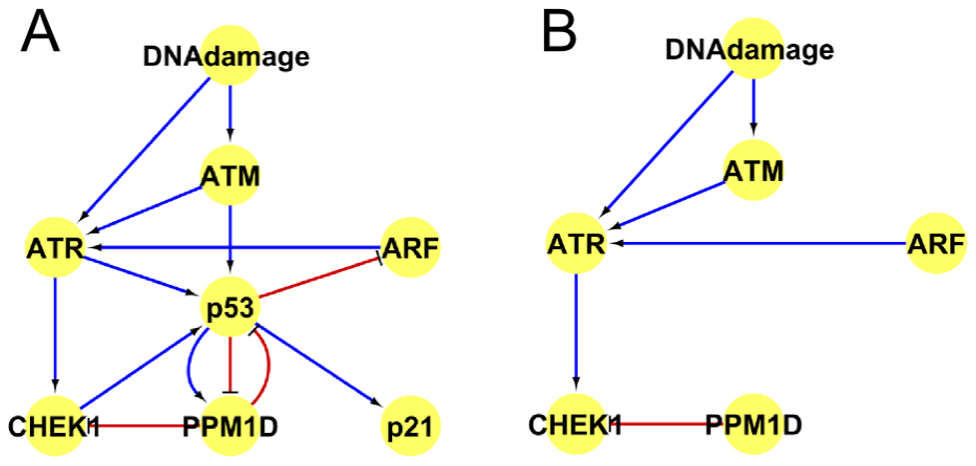
Positive and negative pathways from ATM/ATR to CHEK1. (A) Positive and negative pathways from ATM/ATR to CHEK1 in p53 wild type cells as known from literature survey; (B) Positive and negative pathways from ATM/ATR to CHEK1 in p53 minus cells. ARF is cyclin-dependent kinase inhibitor 2A. PPM1D is protein phosphatase 1D. pRB is retinoblastoma 1.

In addition to literature based validation, we obtained *in vitro* based experimental evidence to support novel predictions of the model. The model predicted that in the absence of functional p53, the effects of ATM and ATR (ataxia telangiectasia and Rad3 related) onto CHEK1 would both change from ambivalent factors to strong activators. A western blot analysis of U2OS human osteosarcoma cells that have wild-type p53, and of SAOS2 cells that have mutant non-functional p53, demonstrated that CHEK1 is activated to a higher extent in the p53 mutant background than in the p53 wild-type background (Figure 4B) validating this prediction. Furthermore, higher levels and potential activation of ATM and ATR kinases was observed in p53 minus cells than in p53 positive cells. According to the model, there are both positive and negative paths between ATM, ATR, CHEK1, and p53 in the wild type cells, and therefore in p53 mutant cells this balance is disturbed (Figure 5). This confirms the predictive capability of our modelling approach and has consequences for treatment of p53 negative tumours.

### Logical steady state analysis

The p53 protein is known to maintain genomic stability and the absence of p53 leads to cellular proliferation in response to DNA damage [31]. The absence of genetic stability triggers the accumulation of mutations in normal cells and causes cancer [32]. In order to investigate how such loss of stability could be captured by our model, we carried out a comparative logical steady state analysis in the p53 wild-type model and *in silico* p53 knock-out.

**Figure 6:**
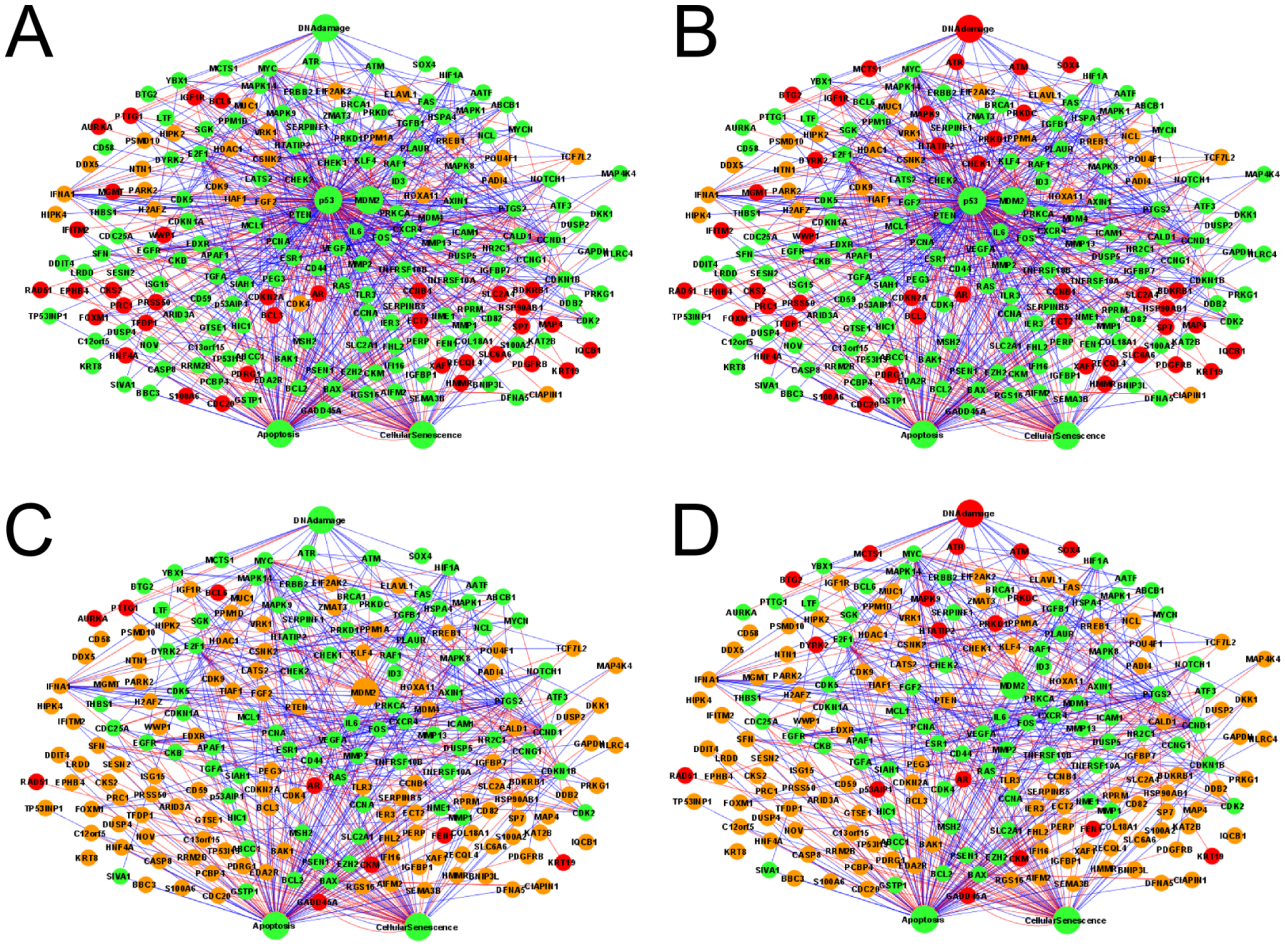
Logical steady state analysis of *in silico* p53 knock-out test. The nodes with state “1” were represented in green, the nodes with state ”NaN” (un determined) were represented in orange, and the nodes with state “0” were represented in red. (A) P53 wild type when DNA damage was ”ON”; (B) P53 wild type when DNA damage was ”OFF”; (C) P53 mutant when DNA damage was ”ON”; (D) P53 mutant when DNA damage was ”OFF”.

In a logical steady state (LSS), the state of each node remains the same over time [33]. Each node can have three different states: inactivated (“0”), activated (“1”) or undetermined (“NaN”). We investigated four scenarios for logical steady state analysis: (1) DNA damage is activated in p53 wild-type background; (2) DNA damage is not activated in p53 wild-type background; (3) DNA damage is activated in p53 knock-out background; (4) DNA damage is not activated in p53 knock-out background (Figure 6, Table 2 and Table S8 in File S1). The comparison of logical steady states in different scenarios revealed that a large number of node states did not change with the change of input signal. This result is explained by the large number of ambivalent effects between nodes and feedback loops in the network, which make the model robust to input signal perturbations. The proportion of determined states was 181 out of 206 nodes (87.9%) in scenario (1), 182 out of 206 nodes (88.3%) in scenario (2), 94 out of 205 nodes (45.9%) in scenario (3) and 95 out of 205 nodes (46.3%) in scenario (4) (Table 2). These numbers show that almost half of the nodes whose state is determined in the wild-type, become undetermined in the *in silico* p53 knock-out.

Comparing the state of 202 genes which interact with p53 in p53 wild type cells in the presence of DNA damage and those in p53 mutant cells in the presence of DNA damage, we found that only 29 genes were up-regulated, 113 genes did not change and 60 genes were down regulated (Table 3). The change of FEN1 (flap structure-specific endonuclease 1) state was moreover experimentally verified by Christmann et al, through finding that FEN1 was repressed in p53 null cells under DNA damage [34]. TLR3 was found to be down-regulated in p53 mutant cells under DNA damage [35].

**Table 3:** Distribution of gene state alterations caused by p53 removal and DNA damage from *in silico* logical steady state analysis.

Source scenario	Target scenario	Number of up-regulated genes	Number of unchanged genes	Number of down-regulated genes
P53 wild type with DNA damage	P53 mutant with DNA damage	29(14%)	113(56%)	60(30%)
P53 wild type without DNA damage	P53 mutant with DNA damage	30(15%)	112(55%)	60(30%)
P53 wild type with DNA damage	P53 wild type without DNA damage	5(2%)	185(92%)	12(6%)
P53 mutant with DNA damage	P53 mutant without DNA damage	7(3%)	181(90%)	14(7%)

The distribution of 202 p53-interacting genes with different changes between four scenarios was calculated using logical steady state analysis.

Comparing the state of these 202 genes in p53 wild type cells in the absence of DNA damage and those in p53 mutant cells in the absence of DNA damage (Table 3), we found that 30 genes were up-regulated, 112 genes remained the same and 60 genes were down-regulated in p53 wild type cells in the absence of DNA damage. The change of 6 nodes were verified by O'Prey et al. [36]. 4 nodes were demonstrated as correct predictions: the expression levels of FAS (TNF receptor superfamily, member 6), TNFRSF10B (tumour necrosis factor receptor superfamily, member 10b), PERP (PERP, TP53 apoptosis effector) and p53AIP1 (tumour protein p53 regulated apoptosis inducing protein 1), were down-regulated from p53 wild type cells without DNA damage to p53 mutant cells without DNA damage, whereas the other 2 nodes MDM2 and CDKN1A were predicted as unchanged by model simulation. However, O'Prey et al. observed their down-regulation from p53 wild type cells without DNA damage to p53 mutant cells without DNA damage [36]. According to the criteria defined in the methods section, four predictions were correct and the other two were small error predictions.

Comparing the state of these 202 nodes in p53 wild type cells in the presence of DNA damage and those nodes in p53 wild type cells in the absence of DNA damage (Table 3), we found that only 5 genes were up-regulated, 185 genes were not changed and 12 genes were down-regulated in p53 wild type cell induced by DNA damage.

Comparing the state of these 202 nodes in p53 mutant cells in the presence of DNA damage and those nodes in p53 mutant cells in the absence of DNA damage (Table 3), we found that 7 genes were up-regulated, 181 genes remained the same and 14 genes were down-regulated in p53 mutant cells induced by DNA damage. Together these above results reflect the fact that p53 helps to stabilize the system.

The changes in the state of anti-apoptotic and anti-senescence genes are shown in Table S9 in File S1 and those of pro-apoptotic and pro-senescence genes are listed in Table S10 in File S1. This distribution illustrates the reason why the apoptosis output was also activated in p53 mutant cells. The majority of those 56 pro-apoptotic genes and 39 anti-apoptotic genes were not changed in the same type of cells treated by DNA damage. The absence of p53 caused obvious changes of both pro-apoptotic and anti-apoptotic genes once the cells were treated with DNA damage. The number of pro-apoptotic and anti-apoptotic genes which were up-regulated or down-regulated increased with the depletion of p53. Among those 56 pro-apoptotic genes, FAS and p53AIP1 were up-regulated in p53 mutant cells when treated by DNA damage. FGF2 (fibroblast growth factor 2(basic)) had both pro-apoptotic and anti-apoptotic function in the PKT206 model and it was down-regulated in p53 wild type cells or p53 mutant cells in the presence of DNA damage. Notably, IGF1R (insulin-like growth factor 1 receptor) and PDGFRB (platelet-derived growth factor receptor, beta polypeptide) were upregulated in p53 minus scenarios, which together with FGF2 changes highlighted growth factor mediated signalling pathways as important factor contributing to survival of these tumours. Approaches that will decrease expression of antiapoptotic genes and increase expression of proapoptotic genes would improve cancer therapy and therefore these genes represent potential therapeutic targets. Two anti-senescence genes were upregulated in the absence of p53, in the presence or absence of DNA damage (CDK4 and FGF2). If DNA damage was applied to either wild type or p53 minus cells, seven anti-senescence genes were increased (Table S9 in File S1). Only three pro-senescence genes increased when DNA damage was applied to wild type or p53 minus cells, and 12 pro-senescence genes decreased in the same conditions (Table S10 in File S1).

### Genome-wide experimental validation

In order to evaluate the predictive capability of our logical model on a genome-wide level, predictions of logical steady state analysis in the *in silico* p53 knock-out were compared with gene expression profiles from microarray analysis. The simulation results of our model were compared with microarray data from 4 different cell types. For this purpose U2OS human osteosarcoma cells that are p53 wild-type and SAOS2 cells that lack functional p53 were treated with the clinically used drug etoposide that causes DNA damage and activates p53. Moreover, we utilized microarray experimental data sets obtained from HCT116 cell lines that have wild type and mutant p53 not treated by DNA damage from GSE10795 [37].

In order to compare both sets of values and evaluate the performance of our model, we used the approach presented by Christensen et al [38]. The predicted change of gene state between p53 wild-type and *in silico* knockout was quantified by a variable *E_mod_* which could take one of three values: −1, 0 or 1 (see Materials and Methods for details). The experimentally observed change of gene state was represented by a variable *E_exp_* which could take the same three values. For both *E_mod_* and *E_exp_*, a value of −1 meant significantly decreased expression, 0 meant no significant change, and 1 meant significantly increased expression.

Using the results of logical steady state analysis, we calculated the value *E_mod_* of those 3 types of different cell lines. We extracted relevant genes in the microarray data whose gene names matched those in our logical model. A threshold *θ* was considered to determine whether a gene was significantly up-regulated, down-regulated or unchanged. In a similar way, we calculated the value *E_exp_* of each gene and listed the number of genes with different changes in Table S11 in File S1.

We then defined the difference between model predictions and experimental results as |*E_mod_ – E_exp_*|. This difference can take three possible values: 0, 1 or 2. Here, a value of 0 meant that the simulation prediction matched the experimental result; 1 meant that there was a small error between the prediction and the experimental result; 2 meant that there was a large error between the prediction and the experimental result. The distribution of values was calculated and listed in Table 4. Comparing the changes of gene states between different scenarios with experimental microarray data, 5 scenarios were analyzed. The correct prediction rate ranged from 52% to 71% depending on the cell type, with highly significant p-values compared to random predictions. The percentage of small error predictions ranged from 28% to 42%, and large error predictions were obtained for less than 6% of the genes depending on the cell type. Remarkably, growth factors and receptors FGF2 and IGF1R were identified as common, and PDGFR and TGFA as specific factors contributing to U2OS human osteosarcoma and HCT116 colon cancer cells growth, respectively (Table S11 in File S1). For example, IGF1R is an anti-apoptotic gene upregulated in SAOS2 cells when compared to U2OS cells, whereas FGF2 that can be both pro and antiapoptotic gene is upregulated in SAOS2 cells. In HCT116 cells, with the mutant p53 similarly to SAOS2, there is upregulation of antiapoptotic IGF1R, but PDGFRB and TGFA (transforming growth factor, alpha) are also upregulated, and FGF2 does not change in these cells (Table S11 in File S1), indicating that both general (IGF1R) and cell type specific (PDGFRB and TGFA) pathways were uncovered by the model. Two anti-senescence genes (DDIT4 and DKK1) were upregulated and three (RRM2B, FGF2, FHL2) were down-regulated in SAOS2 cells in the presence of DNA damage, whereas in the absence of DNA damage S100A6 and DKK1 were upregulated and only FGF2 was downregulated. Interestingly, DDIT4 was downregulated in U2OS cells exposed to DNA damage, but upregulated in SAOS2 exposed to DNA damage. There were more changes among pro-senescence genes, where CDKN1A (p21) featured as a major regulator of cell senescence, amongst growth factors and DNA repair genes (Table S11 in File S1).

**Table 4:** Model evaluation by logical steady state and microarray analysis.

Experiment source scenario	Experiment target scenario	Model LSSA simulation	Total number of genes	Number of true predictions	p-value of true predictions	Number of small error predictions	Number of large error predictions
U2OS cells under DNA damage	SAOS2 cells under DNA damage	P53 wt with DNA damage ON vs p53 null with DNA damage ON	200	109(54.5%)	2.6×10^−10^	80(40%)	11(5.5%)
U2OS cells without DNA damage	SAOS2 cells without DNA damage	P53 wt with DNA damage OFF vs p53 null with DNA damage OFF	200	111(55.5%)	4.1×10^−11^	77(38.5%)	12(6%)
U2OS cells without DNA damage	U2OS cells under DNA damage	P53 wt with DNA damage ON vs p53 wtwith DNA damage OFF	200	142(71%)	<<10^−15^	56(28%)	2(1%)
SAOS2 cells without DNA damage	SAOS2 cells under DNA damage	P53 null with DNA damage ON vs p53 null with DNA damage OFF	200	131(65.5%)	<<10^−15^	65(32.5%)	4(2%)
HCT116 cells p53+/+ without DNA damage	HCT116 cells p53−/−without DNA damage	P53 null with DNA damage OFF vs p53 wt with DNA damage OFF	169	88(52.1%)	1.8×10^−7^	72(42.6%)	9(5.3%)

The changes of gene expression in experimental microarray data were compared with model simulation results. The number of true predictions, small errors, large errors and their percentage were calculated and listed.

## Discussion

p53 acts as a tumour suppressor and plays a crucial role in protecting cells against cancer and genetic instability caused by DNA damage [32]. The loss of p53 function is common in many cancer cells, highlighting its importance for medicine. Since there are thousands of reported gene interactions with p53, we automatically extracted all genes interacting with p53 from the STRING database. This led to a model with more than 2000 nodes that included several layers of direct and indirect p53 interactants. This model was simplified by eliminating indirect interactants, and further manual curation resulted in the generation of the present PKT206 model. The distinction between direct and indirect interactions is important for this type of model. Databases such as STRING contain both direct (physical) and indirect (functional) interactions, which is why manual curation was essential. It is worth noting that the meaning of “direct” in this context can be broader than a direct physical contact between proteins; for example a transcription factor binding to a promoter and inducing the transcription of another gene can be treated as a direct activation, since the processes of transcription/translation are not explicitly represented in the logical model. Finally, the effect of environmental signals such as DNA damage and the outputs, apoptosis and senescence, were added.

The use of Boolean networks in cancer research has been reported in a few other studies. For example, Ghaffari et al. designed a Boolean model of gastrointestinal cancer comprising 17 genes [39]. Chaves et al. constructed a Boolean network with 20 nodes to investigate the dynamics role of the NF-κB (nuclear factor kappa-light-chain-enhancer of activated B cells) pathway in controlling apoptosis [40]. Calzolari et al. designed a Boolean network with 47 genes that regulate apoptosis and investigated the relationship between genes and selective control of cell populations [41]. Zhang et al. constructed a Boolean network for T cell large granular lymphocyte (T-LGL) survival [42], which consisted of 58 nodes and 123 edges and provided an insight into the long-term survival of cytotoxic T lymphocyte (CTL) in T-LGL leukaemia. Mai et al. constructed a Boolean network including 40 nodes involved in apoptotic pathways and demonstrated that apoptosis is an irreversible process [43]. Ge and Qian constructed Boolean networks to investigate the dynamics of negative feedback loops of p53 pathways. They focused on the p53-MDM2 feedback loop and the cyclin G-MDM2 feedback loop [44]. They compared the stochastic Boolean dynamics using a Markov chain model with the synchronized deterministic Boolean dynamics and showed that the deterministic model was able to predict the dominant process in the system. Oscillations and stationary states in these feedback loops were found to be robust to noise. This report focused on two small-scale feedback loops, but these models were not integrated with other p53-interacting proteins or incoming biological signals.

The majority of above described models only cover parts of apoptotic pathways and p53 feedback loops, whereas our model has more extensive coverage of p53 pathways and includes the most complete p53 interactome to date. In addition, we provide evidence of its potential use in individualized cancer therapy through assessment of apoptotic potential of chemotherapy in osteosarcoma and colon cancer. Other authors reported pathways that interact with p53 at the cellular level. A p53-like protein in *Drosophila melanogaster* (Dmp53) was utilized by Lunardi et al. to identify potential interactors which may link to p53 family members in human [45]. cDNAs were screened from the Drosophila Gene Collection (DGC), among which 91 that were not previously reported were found to interact with Dmp53 by a genome-scale *in vitro* expression cloning approach. 41 mammalian orthologs were tested, 37 were found to interact with p53 family members and 24 interacted with p53 directly. However, further validation of these targets only confirmed five relevant interactions. The GTPBP4 protein was found to regulate p53 negatively and the accumulation of GTPBP4 correlated with reduced breast cancer patients survival. Results of this study revealed new interactions with p53 family members and important differences between Dmp53 and human p53 interactors.

The interactome of p53 and its family member p63 in cisplatin chemoresistant cells was investigated by Huang et al. by protein array chip analysis [46]. They found that p53 and one isoform of p63, ΔNp63α, were involved in various protein-protein interactions in tumour cells upon cisplatin exposure: p53 was found to bind 383 proteins and p63 was involved in interactions with 301 proteins. Candidate interactors which bound to p53 and p63 were also assessed by iTRAQ in squamous cell carcinoma (SCC) cells: 444 proteins were found to bind to p53 and 310 to ΔNp63α. Those proteins were classified into p53 specific, ΔNp63αspecific, and p53/ΔNp63α common groups and clustered by Gene Ontology annotations. Phosphorylated and non-phosphorylated ΔNp63α were found to interact with different target proteins and have differential functions in RNA splicing and cell death upon cisplatin exposure. Numerous proteins involved in cell cycle arrest, apoptosis and autophagy were identified in the p53 and p63 interactome in tumour cells exposed to cisplatin treatment. Comparing specific p53 interactors with our PKT206 model, 21 proteins were found in our model, whereas 25 proteins were found in the group of common p53/p63 interacting proteins. Those proteins may be considered for incorporation into our PKT206 model in the future; nevertheless, evidence of binding is not sufficient in itself to justify inclusion into a logical model, since evidence is needed that the interaction results in a measurable effect on the activity of the target protein.

Knock-out simulations allowed us to mimic p53 mutants potentially found in cancer and generate predictions of the effects of DNA damage on cellular fate. The percentage of change for genes that control apoptosis and senescence is shown in Tables S9 and S10 in File S1. These distributions illustrate the probability of cell death and senescence as important mechanisms to be considered for cancer treatment. For instance, in cells with mutant p53 not treated with chemotherapy inducing DNA damage, 29 out of 58 pro-apoptotic genes were down-regulated, 22 pro-apoptotic genes do not change and only 5 pro-apoptotic genes were up-regulated. In addition, 38 out of 39 anti-apoptotic genes remained the same and only FGF2 was down-regulated. This finding illustrated that in tumour cells with p53 mutant, the probability of apoptosis was decreased and cells survived. When cells with the mutant p53 were treated by DNA damage, 2 pro-apoptotic genes, FAS and p53AIP1 became up-regulated, and lead to an increase in apoptosis probability to promote tumour cell death.

Interactions for mutant p53-specific interacting proteins were also explored by Coffill et al. with the use of stable isotope labelling by amino acids in cell culture, mass spectrometry and immunoprecipitation techniques [47]. The authors identified 15 proteins that were bound to mutant p53 specifically but not to p53 wild type. A specific interaction between p53R273H and NRD1 (nardilysin) was reported and validated to play an important role in cellular invasion. Results showed that there are specific protein interactions with mutated p53 in tumour cells that do not occur with wild-type p53. This finding indicates that changes in the p53 interactome resulting from mutations should be incorporated into future models to achieve better clinical predictions.

Analysis of the expression changes of genes that control apoptosis using steady state comparisons between different scenarios, *in silico* and in two different types of cancer cell types, produced several important predictions that may have direct therapeutic implications. First, FGF2 that can both inhibit and activate apoptosis is the only factor altered in DNA damage treated cells that do not have p53 status altered, indicating its important role in p53 mediated apoptosis and highlighting its therapeutic potential. Furthermore, this type of analysis identified seven anti-apoptotic genes that are upregulated in the p53 mutant scenario and potentially contribute to the proliferative and resistant phenotype of p53 minus tumours (Table S9 in File S1). Therefore these genes should be targeted with inhibitors to successfully treat cancer carrying p53 mutations. On the other hand, a large number of pro-apoptotic genes are downregulated in p53 mutant cells according to the model, identifying them as potential therapeutic targets for activation (Table S10 in File S1). Further analysis of the subset of genes relevant to the model that we found changed in microarray data identified that the loss of p53 upregulates the IGFR1 gene in both osteosarcoma and colon cancer cell lines. Remarkably, our data identify growth factors as major level of control of antiapoptotic activities in p53 negative cells irrespectively of DNA damage. IGF1R is upregulated *in silico* and in both SAOS2 and HCT116 p53 minus cells. In addition, PDGFRB, IGFR1R and TGFA are all upregulated in HCT116 p53−/− cell lines when compared to the HCT116 p53+/+ plus cell lines. Our analysis highlighted one factor (IGF1R) that is found upregulated in p53 negative cells in the model and at least two different cancer cell lines, and in addition indicated that different cell lines may have additional growth factor combinations and dependencies, as colon cancer cells not exposed to DNA damage had upregulated PDGFR whereas SAOS2 cell did not, when compared to their p53 positive counterparts. This, together with the mentioned role of FGF2 highlights the crucial role of growth factors and their receptors as therapeutic targets in p53 negative cancer.

The DDIT4 and DKK1 anti-senescence genes were upregulated in SAOS2 cells in the presence of DNA damage when compared to p53 positive U2OS cells (Table S11 in File S1), marking them as potential drug development targets for chemotherapy of p53 negative tumours. Interestingly, DDIT4 was downregulated in U2OS cells exposed to DNA damage, but upregulated in SAOS2 cells exposed to DNA damage, suggesting a crucial role of p53 in regulation of this gene and senescence control. Comparison of colon cancer cells to osteosarcoma cells showed differential regulation of anti-senescence genes (Table S11 in File S1), indicating a certain degree of cell specificity of senescence control. CDKN1A (p21), which is a major regulator of cell senescence among pro-senescence genes and was altered in most cases in addition to growth factors and DNA repair genes, was downregulated in both p53 negative colon cancer and osteosarcoma cells, suggesting that this regulator may be involved in control of senescence in multiple cell types. Several other pro-senescence proteins are altered including CDKN2A and CDKN1B, emphasising the control of cell cycle progression as a major mechanism of senescence control. In addition, IL6 was also detected as a gene with altered expression, providing a link between senescence and inflammation.

The extent of the knock-out effect depends on the connectivity and position of the protein in the network. For example, ATM is upstream of p53 with a connectivity of ten, and it does not involve feedback loops. Therefore, the knock-out of ATM resulted in few changes in the dependency matrix. However, predictions of p53 knock-out tests identified genes which have a significant effect on the whole p53 network. Some of them have been used as cancer drug targets, for example, ERBB2 (v-erb-b2, erythroblastic leukemia viral oncogene homolog 2, neuro/glioblastoma derived oncogene homolog (avian)), and EGFR (epidermal growth factor receptor) are targets in breast cancer treatment [48]; other affected nodes may be potential drug target for cancer treatment.

Logical steady state analysis in the p53 knock-out indicated that negative feedback loops are crucial for the robustness of the p53 system to external perturbations. It is worth noting that there are a large number of possible steady states under a given input condition: these multiple states are represented by “undetermined” values in the logical steady state analysis, which means that these nodes can take different values in different steady states. The results of logical steady state analysis indicated that state changes between different DNA damage input conditions and different p53 status could be predicted with significantly better precision than random. The correct prediction percentage ranged between 52% and 71% depending on the cancer type which substantially exceeds the expected probability of 33.3% (Table 4). Given the qualitative nature of our model, these are very promising values. Some negative errors are unavoidable due to the fact that a Boolean network is an approximation of the real system. It does not take into account continuous changes in gene expression levels and time delays caused by feedback loops. Nevertheless the advantage of the Boolean network approach is its completeness, since it would be unrealistic to model the exact dynamics of so many proteins using differential equations. The limitations of microarray technology may affect the results as well; for example, discrepancies between model predictions and microarray data may be caused by the fact that only one probe is available for CKM (creatine kinase, muscle) when the microarray platform indicated in material and methods was used, thus suggesting the need for a more reliable estimate of the expression of this gene. Finally, our model uses an interaction graph where only two genes are involved in each interaction, but some interactions may require the action of more than two genes. In future, these factors should be considered to refine and develop enhanced versions of the PKT206 model that are based on hypergraphs and are specific for p53 post translational modification isoforms, different cell or cancer types, and other types of input and output signals.

A technique to extend Boolean network analysis was presented by Choi et al., who constructed a Boolean model of the p53 network comprising 16 nodes and 50 links. 160 negative and 218 positive feedback loops were included in this model [49]. The attractor landscape of this network was analyzed in the presence or absence of DNA damage, whereby five interactions were found to play a critical role determining the cellular response. This model was also analyzed using a probabilistic Boolean method and applied to the MCF7 breast cancer cell line to identify potential drug targets that enhance p53-mediated apoptosis. These results indicate that the applications of logical models can be further enhanced through the use of landscape analysis and the incorporation of state transition probabilities. However such approaches may be limited by sharp increases in computational time when applied to large interactome networks.

Several important predictions were obtained from our model, which will help us to get deeper insight into the mechanisms of p53 pathways. Our findings highlighted the possibility of using CHEK1 modulators as a novel cancer therapy. Since there are defects in p53 pathways of most tumour cells, the CHEK1 kinase plays an important role to mediate cell cycle arrest in those tumour cells that lost p53 function. It was found that tumour cells are deficient in G1 checkpoint, and arrest in S and G2 check points to repair DNA damage. The S and G2 checkpoint is mediated by CDC25A (cell division cycle 25 homolog A (*S. pombe*)), which is a target of CHEK1; siRNA (small interfering RNA) targeting CHEK1 was able to prevent the degradation of CDC25A and led to abrogation of the checkpoint [50]. Our model suggested that upon DNA damage, ATM, ATR and CHEK1 were all up-regulated in the absence of p53, and that CHEK1 inhibits CDC25A. Those predictions from our model can better explain why CHEK1 is regarded as a potential chemotherapeutics target for cancer treatments [51]. Furthermore, these predictions indicate that any potential treatment should take into account whether the tumour is p53 positive or negative.

An alternative form of validation for this model could consist in collecting random statements from the literature about indirect effects of some genes on others, then determine what fraction of these effects are predicted by the model. However, due to the large number of genes, the number of possible pairings is substantial and the probability that an effect between two random genes would have been experimentally observed is low. We searched for literature evidence about 100 random pairs of genes but could only find one exploitable example, indicating that “Induction of DUSP5 is dependent on activation of MAPK1” [52]. Our prediction for this pair is that MAPK1 is an ambivalent factor for DUSP5, which is compatible with the reported effect, but not conclusive. To carry out this sort of assessment in a systematic way and detect a statistically viable number of effects in the literature, a different approach would be required using automated text mining. Text mining tools are currently able to detect gene and protein names in scientific articles with good precision, however the automated detection of effects remains an unsolved problem.

The construction and validation of the PKT206 model provides a proof of principle that better understanding of the p53 system can be achieved through a systematic compilation of biological knowledge into large-scale logical models. Predictions from knock-out tests and logical steady state analysis can facilitate the future design of new drug targets and strategies for cancer treatments. This versatile and powerful technology could be adapted for different drug dosage by extending the range of protein activation values. Different models could be created for different p53 isoforms using antibodies specific for p53 isoforms in ChIP-seq (Chromatin Immunoprecipitation sequencing) analysis and superimposing data onto existing model. Furthermore, the model could be improved by testing more predictions *in vitro* and expanding it to include more interactions. For example, recent findings have suggested that p53 interacts with microRNAs at multiple levels [53]. The p53 protein is also involved in controlling processing and maturation of several miR families through Drosha and p68 modulation. On the other side, numerous miRs have been shown to modulate p53 function both positively and negatively, directly and indirectly. This approach can be used in the future to study other proteins and, in conjunction with individual genomic profiling, could be applied to predict how individual patients' pathways compare to standard ones and how they are affected during treatment, providing a step towards personalized medicine.

## Materials and Methods

### Extraction of data from STRING

We selected STRING as the main source of data for model construction. STRING is a protein-protein interaction database which encompasses protein interactions from four sources: genomic context, high throughput experiments, conserved co-expression and previous knowledge by natural language processing [54]. STRING established a confidence score scheme to measure the quality of interaction predictions. The confidence score is a value between 0 and 1; a confidence score of more than 0.7 is regarded as a high confidence level. Using these criteria, we extracted all high confidence human protein interactions using a custom designed Java interface. All interaction records were subsequently manually curated by surveying associated literature references and searching for additional evidence wherever necessary. There were 677 interactions included in the PKT206 model: (1) all direct interactions with p53, (2) all interactions between genes/proteins that interact with p53. These interaction records were listed in a text file, which was further processed into a node transcript and a reaction transcript readable by CellNetAnalyzer. The node transcript includes gene names and the reaction transcript includes interaction types (activation or inhibition) and the names of the two genes participating in the interaction.

### Analysis by CellNetAnalyzer

CellNetAnalyzer is a powerful analysis tool for signal flow models. It uses logical interaction hypergraphs to represent connections and define actions between nodes which can be of two types, activation and inhibition [14]. When several arcs are connected to a node, a Boolean function known as “sum of products” is used to define their combined effects: when several arcs end up in the same node 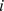 their actions are combined by a logical OR function, and when a hyperarc connects several input nodes to 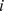 their actions are combined by an AND function. We used two techniques provided by CellNetAnalyzer (v. 9.8) to analyze our model. The first technique is the calculation of the dependency matrix, which represents the effects between all pairs of nodes in the model. CellNetAnalyzer calculates positive and negative paths between two nodes 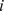 and 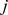 and identifies six types of effects in the dependency matrix: no effect, ambivalent factor, weak inhibitor, weak activator, strong inhibitor, and strong activator as defined below:

1. If there is neither a positive or negative path from node 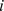 to node 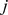, node 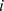 has no effect on node 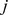;
2. If there is both a positive and negative path from node 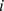 to node 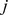, node 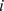 is an ambivalent factor of node 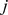;
3. If there are only negative paths from node 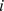 to node 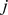 and negative feedback loops are present in these negative paths, node 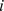 is a weak inhibitor of node 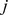;
4. If there are only positive paths from node 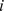 to node 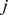 and negative feedback loops are present in these positive paths, node 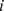 is a weak activator of node 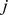;
5. If there are only negative paths from node 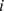 to node 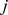 and negative feedback loops are absent in these negative paths, node 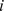 is an strong inhibitor of node 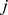;
6. If there are only positive paths from node 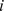 to node 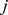 and negative feedback loops are absent in these positive paths, node 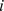 is a strong activator of node 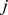.

The second approach used was logical steady state (LSS) analysis. An LSS is a distribution of values over the whole network where the state of each node is fully consistent with the state of incoming interactions applied to the node. Therefore, once a Boolean network has moved into an LSS, it stops to switch and retains this state. In general, there are multiple LSSs in networks that contain feedback loops, and a full enumeration of LSSs can become intractable. Given a set of initial values, in particular for input nodes, CellNetAnalyzer identifies all nodes whose value is uniquely defined across all possible steady states; these nodes are labelled as inactivated (“0”) or activated (“1”). The remaining nodes, whose value is not fully determined by the input conditions and may differ across different LSSs, are labelled as undetermined (“NaN”) [33].

### Cell culture

The p53 wild-type human osteosarcoma cell line U2OS and the p53 null cell line SAOS2 were cultured in Dulbecco's Modified Eagle's Medium (Sigma Aldrich, UK) supplemented with 10% v/v heat inactivated fetal calf serum (Gibco, UK) and 1% of penicillin and streptomycin 10,000 U/ml (Lonza, USA) at 37°C in a humidified atmosphere containing 5% CO_2_. Cells were treated with 10 µM etoposide for 16 hours.

### Immunoblotting and antibodies

Cells were harvested in TNN buffer (50 mM Tris-HCl pH 7.4, 240 mM NaCl, 5 mM EDTA and 0.5% NP-40) and equal amounts of protein were loaded and resolved by SDS-PAGE and Western blotting [55]. After incubating with primary and secondary antibodies, the blots were developed with ECL substrate according to manufacturer's instructions (Pierce, Thermo Scientific, USA). The following antibodies were used for western blotting: β-Actin (Abcam, UK), Chk1 (DCS-300, sc56290, Santa Cruz Biotechnology, Santa Cruz, CA, USA), Phospho Chk1 (Ser 345, sc17922, Santa Cruz Biotechnology, Santa Cruz, CA, USA), ATM (ATM 11g12, Monoclonal antibody, sc53173, Santa Cruz Biotechnology, Santa Cruz, CA, USA ), ATR( 2790 S, NEW ENGLAND BioLabs).

### Microarray preparation and processing

Total RNA was extracted from U2OS and SAOS2 cells treated with vehicle or 10 µM etoposide for 16 hours and RNA extracted using RNeasy plus mini columns (Qiagen, UK) according to the manufacturer's recommendations [56]. For each hybridization, 100 ng of total RNA was used in the Affymetrix GeneChip Two-Cycle Target Labeling kit and in the Ambion MEGAscript T7 kit before hybridizing to the GeneChip human genome U133 Plus 2.0 array (Affymetrix) according to manufacturer's instructions. The heat maps of microarray data were created using the Genesis software, which allows hierarchal clustering and other functional analysis [57]. Our microarray datasets are provided in File S2.

### Model evaluation from microarray data

We compared our predictions obtained by *in silico* deletion of p53 to *in vitro* generated microarray data from p53 positive and p53 negative cell lines treated by the DNA damaging compound etoposide. Logical steady state analysis produces a steady state in each scenario, and changes of gene states can be compared between model predictions and experimental data [38]. For a node 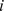, the predicted state of 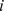 in the p53 wild-type was defined as 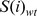, which could take values of 0, 1 or NaN. In the p53 mutant, the state of node 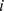 was defined as 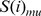, which could take the same values. The value of 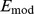
was defined to represent the predicted change of gene state from p53 wild-type to mutant in all 9 types of possible situations as indicated below:


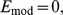
 if 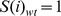 and
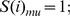



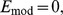
 if 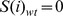 and
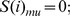



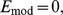
 if 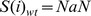
and
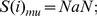



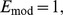
 if 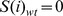 and
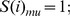



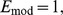
 if 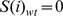 and
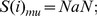



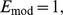
 if 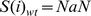 and
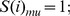



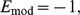
 if 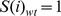 and
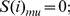



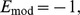
 if 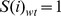 and
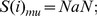



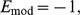
 if 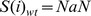
and
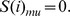


Another parameter 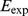 was defined to represent the change trend of expression levels from experimental validation. Those validations were from experimental results of literature survey or microarray analysis result. For experimental results from literature survey:

If the expression level of gene 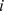 *w*as considered as up-regulated, 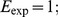


If the expression level of gene 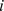 *was* considered as down-regulated, 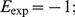


If the expression level of gene 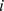 was considered as unchanged, 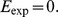


For the microarray data, the gene fold change 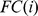 was determined by the following equation:
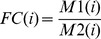
Where 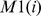
is the median of expression values in the target scenario and 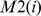
is the median of expression values in the source scenario.

In order to normalize the distributions of expression profiles for different types of cells, the 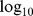
value of all fold changes 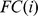 were calculated and two thresholds (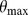
and 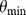) were chosen to determine whether each gene was considered up-regulated, down-regulated or unchanged [58]. The thresholds were determined using the mean value (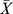) and the standard deviation (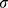) of the distribution of 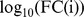 as follows:
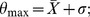


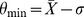



Next, we determined whether the gene was considered up-regulated, down-regulated or unchanged as follows:

If 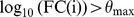, gene 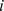 was considered as up-regulated, 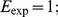


If 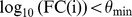, gene 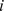 was considered as down-regulated, 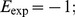


If 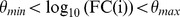, gene 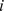 was considered as unchanged, 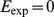.

The difference between 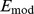 and 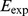 was evaluated by the expression 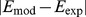. This difference can take three possible values: 0, 1 or 2. Here, a value of 0 meant that the simulation prediction matched the experimental result; 1 meant that there was a small error between the simulation prediction and the experimental result; 2 meant that there was a large error between the simulation prediction and the experimental result, the model predicting an opposite direction of change than experimental results.

## Supporting Information

File S1 **Combined supporting information file containing Figures S1–S5 and Tables S1–S11.**
(DOCX)

File S2 **Gene expression data.** The median values of gene expression levels in microarray experimental data are listed: the data for U2OS and SAOS2 cells are on Sheet 1 and the data for HCT116 cells on Sheet 2. Fold changes for different comparisons and their log_10_ are given.
(XLSX)
