# Supplementary material for: Dynamics of DNA Damage Induced Pathways to Cancer
Source: PLoS One. 2013 Sep 4;8(9):e72303. doi: 10.1371/journal.pone.0072303 (PMC3762865; doi:10.1371/journal.pone.0072303)
Supplement: File S1 — Combined supporting information file containing Figures S1–S5 and Tables S1–S11. (DOCX) [file pone.0072303.s001.docx]

**Figure S1: Incorrect recognition of gene names.**

In the abstract shown below, the sentence “HNE increased p53, p73, p63, p21 and bax expression at different time points” is interpreted as “ELA2 activates p53” (STRING version 9.0, accessed on Feb 8^th^ 2012) ([55](#_ENREF_55)). However, ELA2 is in fact ELANE (elastase, neutrophil expressed) whereas HNE (4-Hydroxynonenal) is IL8 (interleukin 8). These are different genes in PubMed.

**Figure S2: Incorrect recognition of gene name and incorrect capture of key words.**

In the abstract shown below, the sentence ”RITA activates the transcription of proapoptotic p53 targets Noxa, PUMA and BAX” is interpreted as “RITA (ZNF331) activates p53” (STRING version 9.0, accessed on Feb 8^th^ 2012). However, the statement applies to p53 targets and not to p53 itself, but the word “target” was ignored.

**Figure S3: Incorrect recognition of negation and negative words.**

In the abstract shown below, the sentence “Furthermore, small interference RNA (siRNA)-mediated silencing of p53 induced CDC20 expression in normal human dermal fibroblast cells” is interpreted as “p53 activates CDC20” (STRING version 9.0, accessed on Feb 8^th^ 2012). However, the statement indicates that CDC20 in activated by silencing of p53, but the negation was ignored.

**Figure S4:** **Incorrect** **target recognition and incorrect identification of combined protein names.**

In the abstract shown below, the sentence ”These data suggest that the p400 complex inhibits p53--p21 transcription and the development of premature senescence.” is interpreted as “EP400 (p400) inhibits p53” (STRING version 9.0, accessed on Feb 8^th^ 2012). However, the abstract indicates that p21 is regulated by a p53-dependent pathway, not that p53 itself is regulated by p400.

**Figure S5: Example of sub network from the PKT206 model.**

(a) Positive and negative pathways between ATM and CHEK2. Blue arrows represent activations and red arrows represent inhibitions. (b) The interaction matrix for pathways shown in (a). Each row in the interaction matrix represents a node in these pathways and each column represents an interaction. The green cells in the interaction matrix represent the activating input, while the red cells represent the inhibiting input for the interaction displayed in the column. The blue cells in the interaction matrix represent the interaction output, and black cells indicate that the node does not participate in this interaction. (c) The dependency matrix for pathways shown in (a). Each row and each column represent a node in these pathways. Each row of the dependency matrix shows how the corresponding node influences the nodes shown in the columns. The color of each dependency matrix cell *e_AB_* indicates one of the six possible types of dependencies between gene A and gene B: 1) black means that A has no influence on B; 2) yellow means that A has ambivalent (activating and inhibiting) effect on B; 3) light red means that A is a weak inhibitor of B; 4) light green means that A is a weak activator of B; 5) dark red means that A is a strong inhibitor of B; 6) dark green means that A is a strong activator of B.

**Table S1: List of interaction records included in the PKT206 model.**

Column 1 shows the source node of the interaction; column 2 shows the target node of the interaction; column 3 shows the interaction type where -1 means inhibition and +1 means activation; column 4 shows the PubMed identifier of the references used to justify the interaction.

**Table S2: Incorrect interactions not retained in the PKT206 model.**

Column 1 shows the source node of the interaction; column 2 shows the target node of the interaction; column 3 shows the interaction type where -1 means inhibition and +1 means activation; column 4 shows the error type and reason for not retaining the interaction; column 5 shows the PubMed identifier of the references used.

**Table S3: List of links between DNA damage and nodes in the PKT206 model.**

Column 1 shows the source node of the interaction; column 2 shows the target node of the interaction; column 3 shows the interaction type where -1 means inhibition and +1 means activation; column 4 shows the PubMed identifier of references used; column 5 shows the associated GO (Gene Ontology) term.

**Table S4: List of links between nodes in the PKT206 model and apoptosis.**

Column 1 shows the source node of the interaction; column 2 shows the target node of the interaction; column 3 shows the interaction type where -1 means anti-apoptotic and +1 means pro-apoptotic; column 4 shows the PubMed identifier of the references used; column 5 shows the GO (Gene Ontology) term.

**Table S5: List of links between nodes in the PKT206 model and senescence.**

Column 1 shows the source node of the interaction; column 2 shows the target node of the interaction; column 3 shows the interaction type where -1 means repressor of senescence and +1 means inducer of senescence; column 4 shows the PubMed identifier of the references used; column 5 shows the GO (Gene Ontology) term.

**Table S6: Alterations in dependency matrix upon gene deletions.**

A single gene was removed from the PKT206 model for each knock-out test shown below. The numbers of six types of effect elements in the dependency matrix were calculated and listed. The value “Null” in the selected gene column indicates p53 wild type.

**Table S7: Major changes observed in *in silico* gene deletions.**

This table lists all elements from the dependency matrix of the p53 wild-type that were changed to strong inhibitor or strong activator in the dependency matrix of the selected gene deletion.

**Table S8: Logical steady state analysis results of the PKT206 model.**

This table lists the results of logical steady state analysis for four scenarios.

**Table S9: Number of anti-apoptotic genes and genes that prevent cellular senescence with altered expression depending on p53 and DNA damage.**

(a) The numbers of anti-apoptotic genes in the PKT206 model that change expression between four different scenarios were calculated by comparing the steady state of the source and the target scenario. (b) The numbers of genes that prevent cellular senescence in the PKT206 model and change expression between four different scenarios were calculated by comparing the steady state of the source and the target scenario.

**Table S10: Number of pro-apoptotic genes and genes that promote cellular senescence with altered expression depending on p53 and DNA damage**

(a) The numbers of pro-apoptotic genes in the PKT206 model that change expression between four different scenarios were calculated by comparing the steady state of the source and the target scenario. (b) The numbers of genes that promote cellular senescence in the PKT206 model and change expression between four different scenarios were calculated by comparing the steady state of the source and the target scenario.

**Table S11: Number of pro- and anti-apoptotic genes with altered expression depending on p53 and DNA damage in human osteosarcoma and colon cancer cell lines.**

(a) All genes from the PKT206 model were identified in microarray analysis of U2OS (p53 positive), SAOS2 (p53 negative) human osteosarcome (200 genes from the model were analysed) and HCT116 (p53 positive and negative) human colon cancer cell lines (169 genes from the model were analysed). (b) Pro-apoptotic genes from the PKT206 model identified in microarray analysis. (c) Anti-apoptotic genes from the PKT206 model identified in microarray analysis. Results were obtained by comparing the steady state of the source and target scenario. (d) Genes that prevent cellular senescence from the PKT206 model identified in microarray analysis. (e) Genes that promote cellular senescence from the PKT206 model identified in microarray analysis. Results were obtained by comparing the steady state of the source and target scenario.

**Figure S1: Incorrect recognition of gene names.**

In the abstract shown below, the sentence “HNE increased p53, p73, p63, p21 and bax expression at different time points” is interpreted as “ELA2 activates p53” (STRING version 9.0, accessed on Feb 8^th^ 2012) ([55](#_ENREF_55)). However, ELA2 is in fact ELANE (elastase, neutrophil expressed) whereas HNE (4-Hydroxynonenal) is IL8 (interleukin 8). These are different genes in PubMed.

**
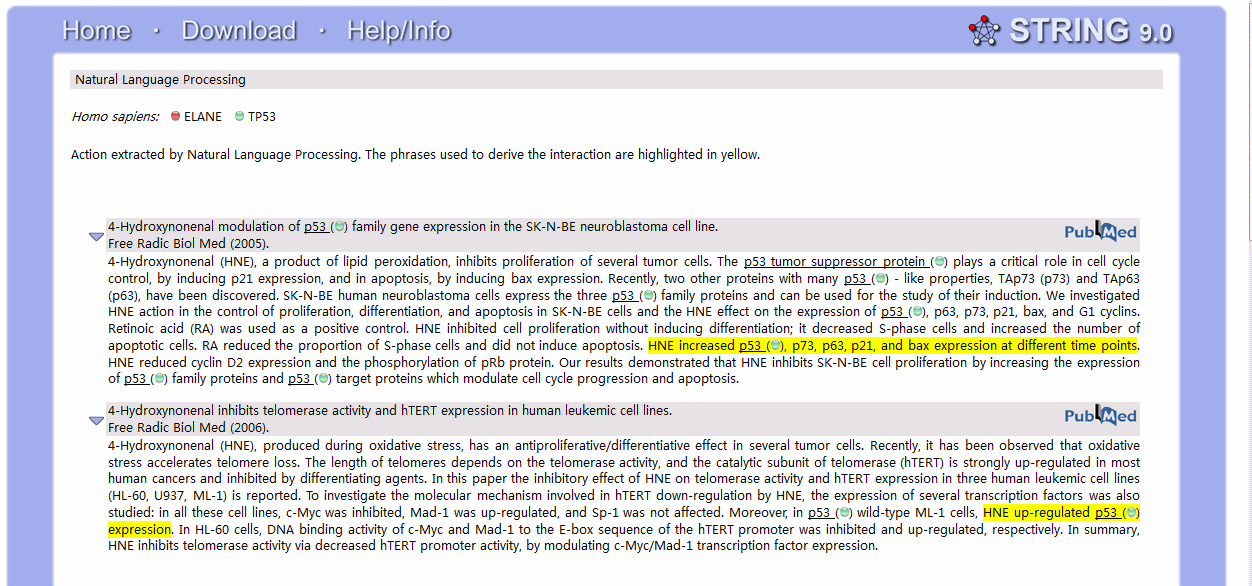
**

**Figure S2: Incorrect recognition of gene name and incorrect capture of key words.**

In the abstract shown below, the sentence ”RITA activates the transcription of proapoptotic p53 targets Noxa, PUMA and BAX” is interpreted as “RITA (ZNF331) activates p53” (STRING version 9.0, accessed on Feb 8^th^ 2012). However, the statement applies to p53 targets and not to p53 itself, but the word “target” was ignored.


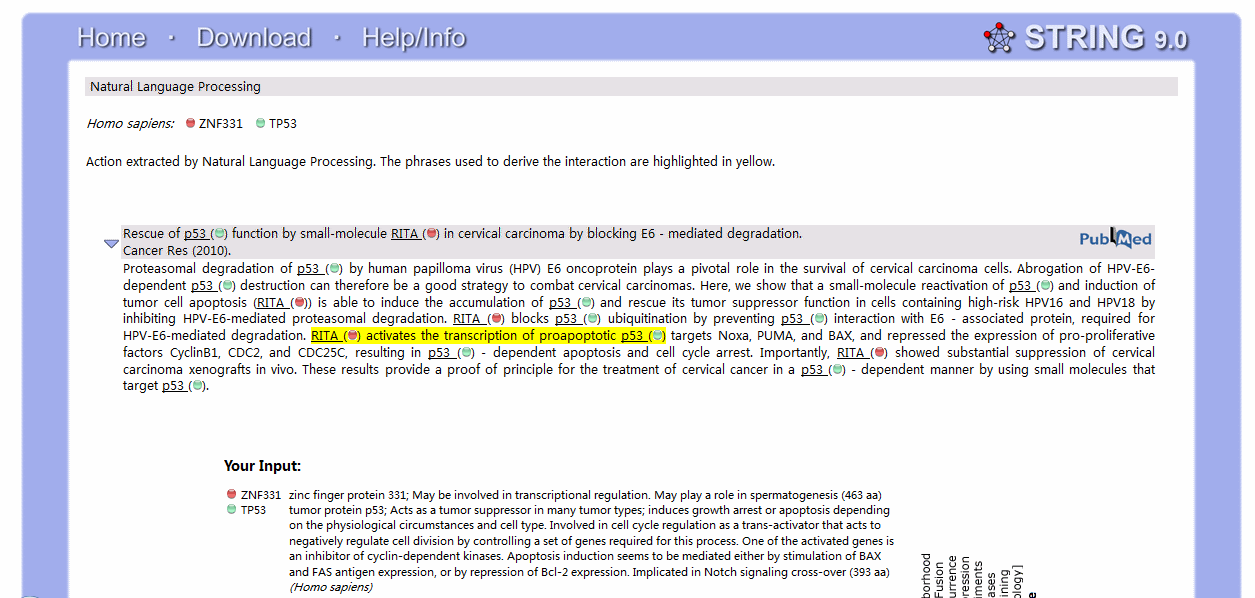


**Figure S3: Incorrect recognition of negation and negative words.**

In the abstract shown below, the sentence “Furthermore, small interference RNA (siRNA)-mediated silencing of p53 induced CDC20 expression in normal human dermal fibroblast cells” is interpreted as “p53 activates CDC20” (STRING version 9.0, accessed on Feb 8^th^ 2012). However, the statement indicates that CDC20 in activated by silencing of p53, but the negation was ignored.


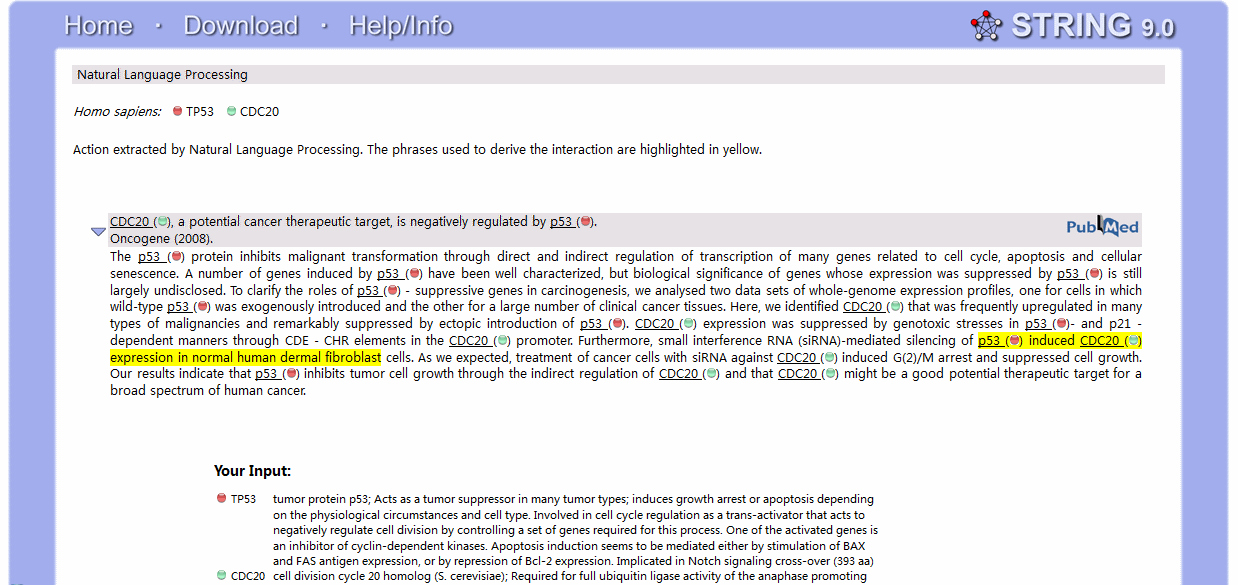


**Figure S4:** **Incorrect** **target recognition and incorrect identification of combined protein names.**

In the abstract shown below, the sentence ”These data suggest that the p400 complex inhibits p53--p21 transcription and the development of premature senescence.” is interpreted as “EP400 (p400) inhibits p53” (STRING version 9.0, accessed on Feb 8^th^ 2012). However, the abstract indicates that p21 is regulated by a p53-dependent pathway, not that p53 itself is regulated by p400.


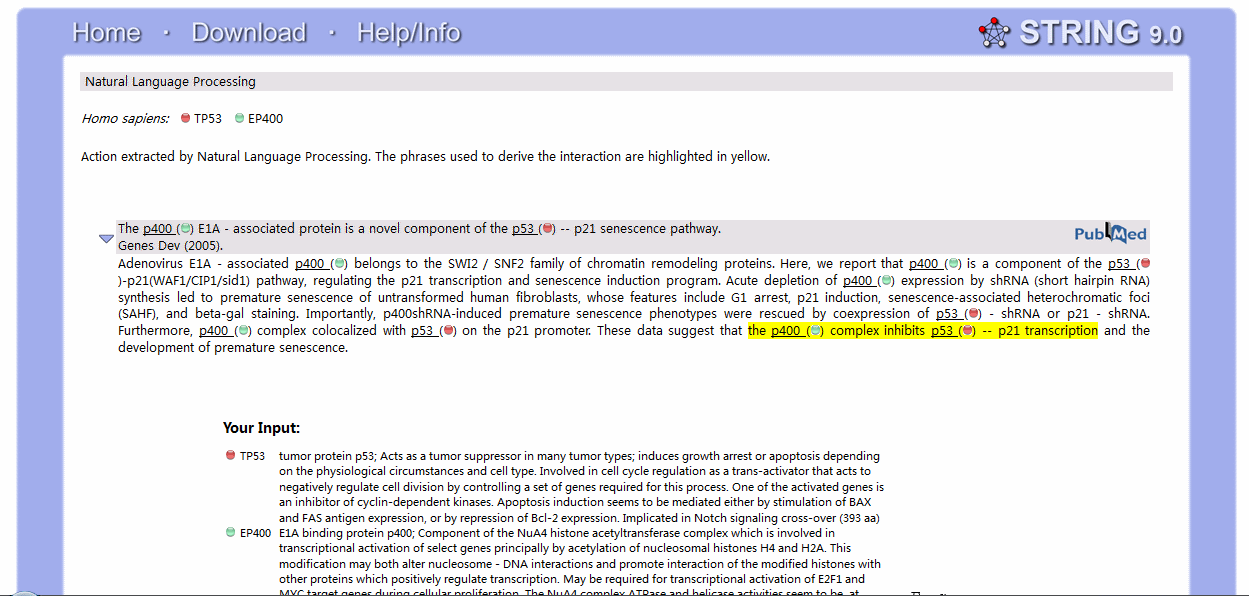


**Figure S5: Example of sub network from the PKT206 model.**

(a) Positive and negative pathways between ATM and CHEK2. Blue arrows represent activations and red arrows represent inhibitions. (b) The interaction matrix for pathways shown in (a). Each row in the interaction matrix represents a node in these pathways and each column represents an interaction. The green cells in the interaction matrix represent the activating input, while the red cells represent the inhibiting input for the interaction displayed in the column. The blue cells in the interaction matrix represent the interaction output, and black cells indicate that the node does not participate in this interaction. (c) The dependency matrix for pathways shown in (a). Each row and each column represent a node in these pathways. Each row of the dependency matrix shows how the corresponding node influences the nodes shown in the columns. The colour of each dependency matrix cell *e_AB_* indicates one of the six possible types of dependencies between gene A and gene B: 1) black means that A has no influence on B; 2) yellow means that A has ambivalent (activating and inhibiting) effect on B; 3) light red means that A is a weak inhibitor of B; 4) light green means that A is a weak activator of B; 5) dark red means that A is a strong inhibitor of B; 6) dark green means that A is a strong activator of B.

**(a)**


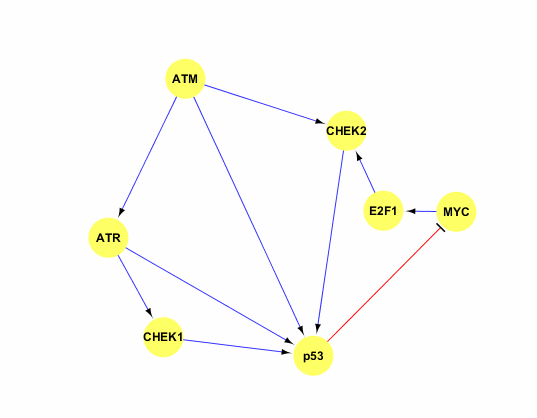


**(b) (c)**


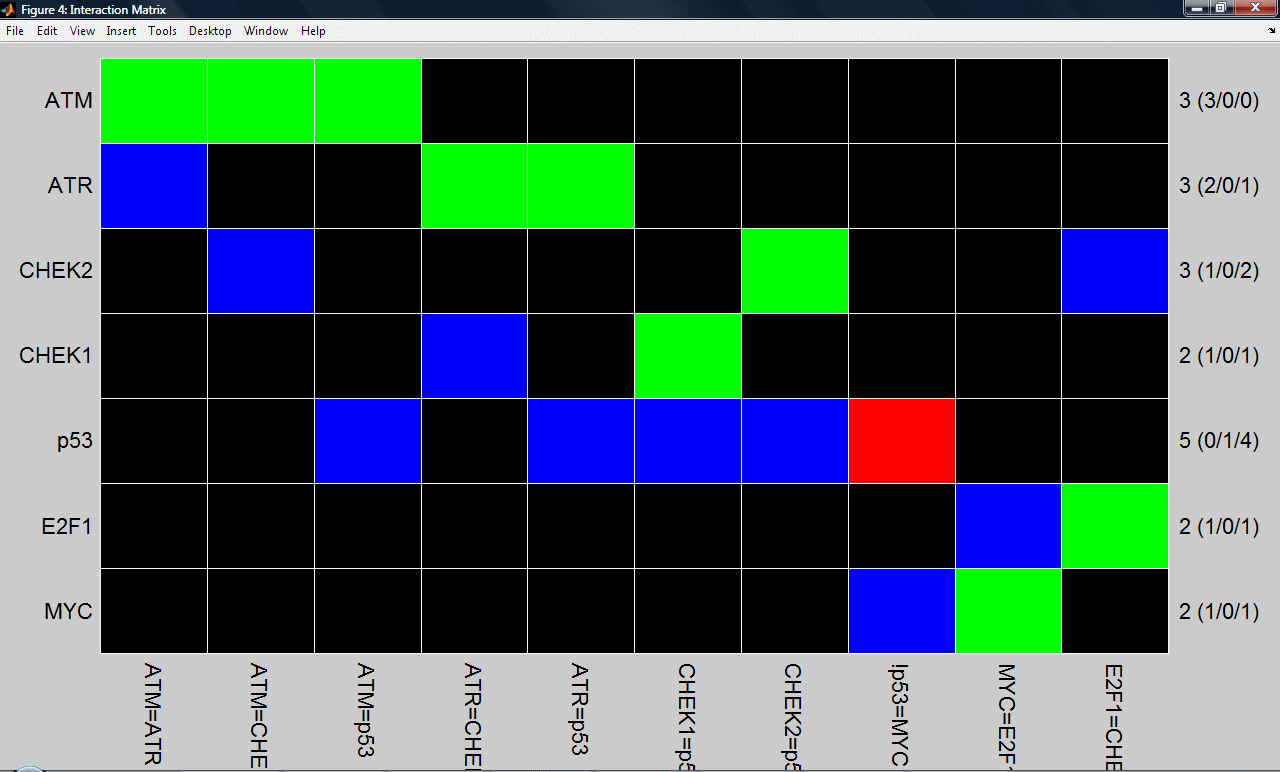

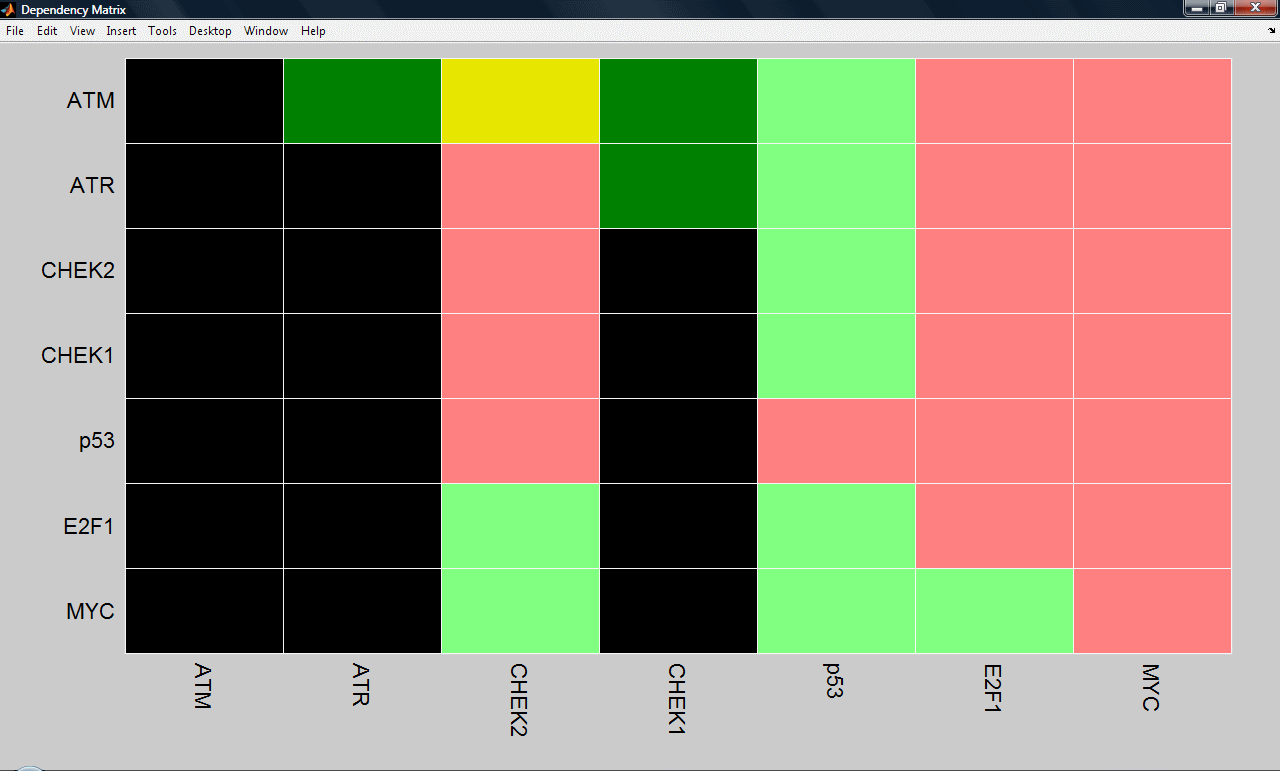


**Table S1: List of interaction records included in the PKT206 model.**

Column 1 shows the source node of the interaction; column 2 shows the target node of the interaction; column 3 shows the interaction type where -1 means inhibition and +1 means activation; column 4 shows the PubMed identifier of the references used to justify the interaction.

| Node A | Node B | Interaction type | PMID |
| --- | --- | --- | --- |
| HOXA11 | P53 | -1 | PMID:19372592 |
| NTN1 | P53 | -1 | PMID:18922894 |
| PSMD10 | P53 | -1 | PMID:19287195 |
| TGFB1 | P53 | +1 | PMID:11741524 |
| AATF | P53 | +1 | PMID:20708154 |
| BCL6 | P53 | +1 | PMID:18524763 |
| BCL6 | P53 | -1 | PMID:15577913 |
| SOX4 | P53 | +1 | PMID:19234109 |
| SERPINF1 | P53 | +1 | PMID:17651710 |
| MDM2 | P53 | -1 | PMID:21423613 |
| DDX5 | P53 | +1 | PMID:15660129 |
| ELAVL1 | P53 | +1 | PMID:12821781 |
| IGF1R | P53 | +1 | PMID:8710868 |
| p53 | ABCC1 | +1 | PMID:12647018 |
| p53 | BCL3 | -1 | PMID:12808109 |
| p53 | GSTP1 | +1 | PMID:18505928 |
| p53 | PDRG1 | -1 | PMID:20453924 |
| p53 | ESR1 | +1 | PMID:15012604 |
| p53 | BDKRB1 | -1 | PMID:11400161 |
| p53 | MMP2 | +1 | PMID:9343394 |
| p53 | CKM | +1 | PMID:7488858 |
| p53 | CD82 | +1 | PMID:11212267 |
| p53 | CCND1 | -1 | PMID:12808109 |
| p53 | GAPDH | +1 | PMID:18552833 |
| p53 | ECT2 | -1 | PMID:16778203 |
| p53 | MSH2 | +1 | PMID:10984493 |
| p53 | CD58 | +1 | PMID:11812190 |
| p53 | DUSP4 | +1 | PMID:16778175 |
| p53 | CXCR4 | -1 | PMID:17130833 |
| p53 | RRM2B | +1 | PMID:19010910 |
| p53 | EDA2R | +1 | PMID:20434500 |
| p53 | SLC6A6 | -1 | PMID:16734743 |
| p53 | XAF1 | -1 | PMID:20198350 |
| p53 | DDB2 | +1 | PMID:12509284 |
| p53 | CDK4 | -1 | PMID:7851794 |
| p53 | MDM2 | +1 | PMID:11313969 |
| p53 | IL6 | -1 | PMID:11830554 |
| p53 | NOV | +1 | PMID:18418052 |
| p53 | THBS1 | +1 | PMID:12609716 |
| p53 | THBS1 | -1 | PMID:9849855 |
| p53 | PDGFRB | -1 | PMID:18697203 |
| p53 | ARID3A | +1 | PMID:12136662 |
| p53 | KAT2B | +1 | PMID:15153330 |
| p53 | FGF2 | -1 | PMID:11313915 |
| p53 | ICAM1 | +1 | PMID:15247038 |
| p53 | WWP1 | -1 | PMID:16924229 |
| p53 | ABCB1 | +1 | PMID:17666793 |
| p53 | ABCB1 | -1 | PMID:11920581 |
| p53 | IGF1R | -1 | PMID:10023442 |
| p53 | NR2C1 | -1 | PMID:8663350 |
| p53 | EGFR | +1 | PMID:19597475 |
| p53 | EGFR | -1 | PMID:18391986 |
| p53 | IGFBP1 | +1 | PMID:18056423 |
| p53 | CD44 | -1 | PMID:18614011 |
| p53 | CDKN1A | +1 | PMID:17585201 |
| p53 | PRKCA | -1 | PMID:15563462 |
| p53 | DUSP2 | +1 | PMID:16474395 |
| p53 | FDXR | +1 | PMID:12370809 |
| p53 | BAX | +1 | PMID:18949380 |
| p53 | KRT8 | +1 | PMID:8615594 |
| p53 | TGFA | +1 | PMID:7651386 |
| p53 | IFI16 | +1 | PMID:18974396 |
| p53 | TLR3 | +1 | PMID:18779317 |
| p53 | CKB | -1 | PMID:7969181 |
| p53 | TP53I13 | +1 | PMID:14767535 |
| p53 | RECQL4 | -1 | PMID:15674334 |
| P53 | MGMT | -1 | PMID:19846904 |
| P53 | SP7 | -1 | PMID:16380437 |
| P53 | HSPA4 | -1 | PMID:8418500 |
| P53 | HSPA4 | +1 | PMID:20180806 |
| HSPA4 | P53 | +1 | PMID:17278883 |
| P53 | CDC25A | -1 | PMID:17001315 |
| P53 | FEN1 | +1 | PMID:16103874 |
| P53 | FOS | -1 | PMID:1946467 |
| P53 | DDIT4 | +1 | PMID:19210572 |
| P53 | CDC20 | -1 | PMID:17873905 |
| P53 | IQCB1 | -1 | PMID:16322217 |
| P53 | MAP4K4 | +1 | PMID:15958553 |
| P53 | HIC1 | +1 | PMID:7585125 |
| P53 | SEMA3B | +1 | PMID:11922394 |
| P53 | RPRM | +1 | PMID:11313928 |
| P53 | EZH2 | -1 | PMID:15208672 |
| P53 | MMP1 | -1 | PMID:11850838 |
| P53 | PSEN1 | -1 | PMID:18374905 |
| P53 | PEG3 | +1 | PMID:11679586 |
| P53 | TSP50 | -1 | PMID:17283160 |
| P53 | IFITM2 | -1 | PMID:19544527 |
| P53 | SIVA1 | +1 | PMID:19240372 |
| P53 | HSP90AB1 | -1 | PMID:15284248 |
| P53 | BCL2 | -1 | PMID:11313951 |
| P53 | PCBP4 | +1 | PMID:11313928 |
| P53 | HIF1A | -1 | PMID:18815219 |
| P53 | DFNA5 | +1 | PMID:16897187 |
| P53 | MMP13 | -1 | PMID:11850838 |
| P53 | CD59 | +1 | PMID:11812190 |
| P53 | FOXM1 | -1 | PMID:19806025 |
| P53 | HNF4A | -1 | PMID:16895524 |
| P53 | TP53INP1 | +1 | PMID:12438758 |
| P53 | FHL2 | +1 | PMID:17352216 |
| P53 | CCNG1 | +1 | PMID:21447558 |
| P53 | TCF7L2 | -1 | PMID:14990988 |
| P53 | BRCA1 | -1 | PMID:12802282 |
| P53 | EPHB4 | -1 | PMID:16205642 |
| P53 | CASP8 | +1 | PMID:12376477 |
| P53 | HMMR | -1 | PMID:18971636 |
| P53 | CDKN1B | +1 | PMID:12376477 |
| P53 | COL18A1 | +1 | PMID:15958553 |
| P53 | BBC3 | +1 | PMID:18657356 |
| P53 | APAF1 | +1 | PMID:11559530 |
| P53 | MAP4 | -1 | PMID:10521394 |
| P53 | BAK1 | +1 | PMID:15105295 |
| P53 | NLRC4 | +1 | PMID:15580302 |
| P53 | PRC1 | -1 | PMID:15531928 |
| P53 | GTSE1 | +1 | PMID:11313928 |
| P53 | CALD1 | +1 | PMID:19349302 |
| P53 | KRT19 | -1 | PMID:7515894 |
| P53 | ATF3 | +1 | PMID:17108111 |
| P53 | PTGS2 | +1 | PMID:15608668 |
| P53 | PTGS2 | -1 | PMID:15921850 |
| P53 | RGS16 | +1 | PMID:16405749 |
| P53 | SGK | +1 | PMID:16619268 |
| P53 | S100A2 | +1 | PMID:18388131 |
| P53 | S100A6 | -1 | PMID:18714402 |
| P53 | MCL1 | -1 | PMID:18208354 |
| P53 | DUSP5 | +1 | PMID:12944906 |
| P53 | IER3 | +1 | PMID:9781666 |
| P53 | GADD45A | +1 | PMID:18350249 |
| P53 | NOTCH1 | +1 | PMID:17534448 |
| P53 | FAS | +1 | PMID:9841917 |
| P53 | PTEN | +1 | PMID:11729185 |
| P53 | VEGFA | -1 | PMID:11559575 |
| P53 | SLC2A1 | -1 | PMID:21862591 |
| P53 | AIFM2 | +1 | PMID:15273740 |
| P53 | CDKN1A | +1 | PMID:17585201 |
| P53 | DKK1 | +1 | PMID:15668788 |
| P53 | PRKG1 | +1 | PMID:19955367 |
| P53 | AR | -1 | PMID:18084622 |
| P53 | TFDP1 | -1 | PMID:9556576 |
| P53 | CKS2 | -1 | PMID:17336302 |
| P53 | NME1 | +1 | PMID:12669312 |
| P53 | NME1 | -1 | PMID:12669312 |
| P53 | MYC | -1 | PMID:8479742 |
| P53 | PCNA | +1 | PMID:11682006 |
| P53 | PCNA | -1 | PMID:8570655 |
| P53 | C13orf15 | +1 | PMID:17146433 |
| P53 | ISG15 | +1 | PMID:11462054 |
| P53 | BNIP3L | +1 | PMID:15607964 |
| P53 | SLC2A4 | -1 | PMID:20729871 |
| P53 | CCNB1 | -1 | PMID:11162602 |
| P53 | TNFRSF10A | +1 | PMID:15289308 |
| P53 | TNFRSF10B | +1 | PMID:10942251 |
| P53 | IGFBP7 | +1 | PMID:19638426 |
| P53 | LATS2 | +1 | PMID:19855428 |
| LATS2 | P53 | +1 | PMID:17015431 |
| P53 | RAD51 | -1 | PMID:19942681 |
| P53 | SERPINB5 | +1 | PMID:15578720 |
| P53 | RAS | +1 | PMID:11574421 |
| ERBB2 | P53 | -1 | PMID:8700512 |
| MYCN | P53 | +1 | PMID:20145147 |
| HSPA4 | P53 | +1 | PMID:17278883 |
| ZMAT3 | P53 | +1 | PMID:12196512 |
| NCL | P53 | -1 | PMID:16213212 |
| NCL | MDM2 | -1 | PMID:16751805 |
| KLF4 | P53 | +1 | PMID:19696146 |
| KLF4 | P53 | -1 | PMID:16244670 |
| PPM1A | P53 | +1 | PMID:12514180 |
| PLAUR | P53 | -1 | PMID:17110957 |
| HTATIP2 | P53 | +1 | PMID:18519672 |
| PTTG1 | P53 | +1 | PMID:15242522 |
| PTTG1 | P53 | -1 | PMID:19477929 |
| E2F1 | P53 | +1 | PMID:12625370 |
| TIAF1 | P53 | +1 | PMID:14965474 |
| CIAPIN1 | P53 | -1 | PMID:16410721 |
| BRCA1 | P53 | +1 | PMID:11371136 |
| PARK2 | P53 | -1 | PMID:19801972 |
| BTG2 | P53 | +1 | PMID:11814693 |
| SGK | P53 | -1 | PMID:19756449 |
| MUC1 | P53 | -1 | PMID:15710329 |
| MCTS1 | P53 | -1 | PMID:17416211 |
| FAS | P53 | +1 | PMID:9358752 |
| YBX1 | P53 | -1 | PMID:12835324 |
| YBX1 | P53 | +1 | PMID:11175333 |
| HDAC1 | P53 | -1 | PMID:12426395 |
| ID3 | P53 | -1 | PMID:19618124 |
| PADI4 | P53 | -1 | PMID:18499678 |
| POU4F1 | P53 | +1 | PMID:10329733 |
| MYC | P53 | +1 | PMID:9839551 |
| RREB1 | P53 | +1 | PMID:19558368 |
| IFNA1 | P53 | +1 | PMID:15254403 |
| CHEK2 | P53 | +1 | PMID:10673500 |
| AXIN1 | P53 | +1 | PMID:19513548 |
| P53 | CCNA | -1 | PMID:8270002 |
| H2AFZ | P53 | -1 | PMID:17671089 |
| TGFB1 | IL6 | +1 | PMID:2265243 |
| TGFB1 | IL6 | -1 | PMID:2265243 |
| TGFB1 | THBS1 | +1 | PMID:11955611 |
| THBS1 | TGFB1 | +1 | PMID:11021838 |
| TGFB1 | ICAM1 | -1 | PMID:14500551 |
| TGFB1 | PRKCA | +1 | PMID:14749204 |
| TGFB1 | MMP1 | -1 | PMID:16911716 |
| TGFB1 | MMP13 | -1 | PMID:9009143 |
| TGFB1 | CCNG1 | +1 | PMID:9696022 |
| TGFB1 | KRT19 | -1 | PMID:14732924 |
| TGFB1 | PTGS2 | +1 | PMID:10935498 |
| TGFB1 | PTEN | -1 | PMID:19940030 |
| TGFB1 | VEGFA | +1 | PMID:12615726 |
| VEGFA | TGFB1 | -1 | PMID:19180561 |
| TGFB1 | CDKN1A | +1 | PMID:7696178 |
| DKK1 | TGFB1 | +1 | PMID:20019166 |
| TGFB1 | MYC | -1 | PMID:12628347 |
| MAPK8 | TGFB1 | +1 | PMID:12760970 |
| AATF | CDK5 | +1 | PMID:18388733 |
| ESR1 | LTF | +1 | PMID:15525592 |
| LTF | CCND1 | -1 | PMID:18697201 |
| SERPINF1 | VEGFA | -1 | PMID:16901919 |
| VEGFA | SERPINF1 | +1 | PMID:12670505 |
| VEGFA | SERPINF1 | -1 | PMID:16901919 |
| IGF1R | MDM2 | +1 | PMID:17846171 |
| MDM2 | ABCB1 | +1 | PMID:8883415 |
| MDM2 | HIF1A | +1 | PMID:15024078 |
| MDM2 | VEGFA | -1 | PMID:18199551 |
| E2F1 | MDM2 | -1 | PMID:20837136 |
| IFNA1 | MDM2 | +1 | PMID:15580300 |
| HIPK2 | MDM2 | -1 | PMID:16212962 |
| ELAVL1 | CDKN1B | -1 | PMID:18354415 |
| ELAVL1 | PTGS2 | +1 | PMID:14633672 |
| ELAVL1 | MYC | -1 | PMID:19574298 |
| MYCN | ABCC1 | +1 | PMID:14737110 |
| BCL3 | EGFR | +1 | PMID:17881446 |
| RAS | GSTP1 | +1 | PMID:11606500 |
| ESR1 | CCND1 | +1 | PMID:15544931 |
| ESR1 | IL6 | -1 | PMID:16043358 |
| ESR1 | EGFR | -1 | PMID:9269899 |
| ESR1 | CKB | +1 | PMID:11746525 |
| NOTCH1 | ESR1 | +1 | PMID:19838210 |
| ESR1 | MYC | +1 | PMID:19661132 |
| IL6 | MMP2 | +1 | PMID:16934628 |
| FGF2 | MMP2 | +1 | PMID:19107653 |
| MMP2 | BAX | +1 | PMID:16857167 |
| BCL2 | MMP2 | +1 | PMID:19258038 |
| FOXM1 | MMP2 | +1 | PMID:17804744 |
| ATF3 | MMP2 | -1 | PMID:11792711 |
| VEGFA | MMP2 | +1 | PMID:16584583 |
| SLC2A1 | MMP2 | +1 | PMID:12122099 |
| MYC | MMP2 | +1 | PMID:19258038 |
| HTATIP2 | MMP2 | -1 | PMID:19349353 |
| PTTG1 | MMP2 | +1 | PMID:19433493 |
| YBX1 | MMP2 | +1 | PMID:9278454 |
| RAS | MAPK14 | +1 | PMID:15677464 |
| MAPK14 | MMP2 | +1 | PMID:15677464 |
| MAPK8 | MMP2 | +1 | PMID:16672691 |
| RAS | CKM | -1 | PMID:3600660 |
| CCNA | CKM | -1 | PMID:8995365 |
| CDK4 | CCND1 | +1 | PMID:9099745 |
| CCND1 | THBS1 | -1 | PMID:17020778 |
| PDGFRB | CCND1 | +1 | PMID:10688905 |
| EGFR | CCND1 | +1 | PMID:19935697 |
| CD44 | CCND1 | -1 | PMID:17296798 |
| PRKCA | CCND1 | -1 | PMID:20141613 |
| TGFA | CCND1 | +1 | PMID:9407106 |
| CCND1 | HSPA4 | +1 | PMID:9121772 |
| FOS | CCND1 | +1 | PMID:9710644 |
| BCL2 | CCND1 | +1 | PMID:11313702 |
| BRCA1 | CCND1 | +1 | PMID:17278098 |
| ATF3 | CCND1 | +1 | PMID:11375399 |
| PTEN | CCND1 | -1 | PMID:16849370 |
| MYC | CCND1 | +1 | PMID:7526316 |
| RAS | CCND1 | +1 | PMID:10201372 |
| ERBB2 | CCND1 | +1 | PMID:17483350 |
| CCND1 | E2F1 | +1 | PMID:10504464 |
| CDKN2A | CCND1 | -1 | PMID:15205322 |
| BTG2 | CCND1 | -1 | PMID:15378000 |
| MUC1 | CCND1 | +1 | PMID:14688481 |
| MCTS1 | CCND1 | +1 | PMID:11709712 |
| AXIN1 | CCND1 | -1 | PMID:11739413 |
| RAS | CCND1 | -1 | PMID:12081197 |
| MAPK1 | CCND1 | +1 | PMID:9618377 |
| MAPK8 | CCND1 | +1 | PMID:21135252 |
| MYC | MSH2 | +1 | PMID:15814658 |
| IL6 | CXCR4 | +1 | PMID:9933168 |
| TLR3 | CXCR4 | -1 | PMID:19652552 |
| HIF1A | CXCR4 | +1 | PMID:19212630 |
| PTGS2 | CXCR4 | +1 | PMID:20110411 |
| CXCR4 | VEGFA | +1 | PMID:17559806 |
| VEGFA | CXCR4 | +1 | PMID:19391039 |
| CXCR4 | TNFRSF10B | +1 | PMID:15990565 |
| ERBB2 | CXCR4 | +1 | PMID:15542424 |
| IFNA1 | CXCR4 | +1 | PMID:18202009 |
| FGF2 | CDK4 | +1 | PMID:11889462 |
| CDK4 | BRCA1 | +1 | PMID:10660629 |
| CDK4 | BRCA1 | -1 | PMID:17334399 |
| IL6 | EGFR | +1 | PMID:9459124 |
| PRKCA | IL6 | +1 | PMID:9523575 |
| IL6 | BAX | -1 | PMID:12101271 |
| IL6 | TGFA | +1 | PMID:11892999 |
| IL6 | FOS | +1 | PMID:11713282 |
| IL6 | MMP1 | +1 | PMID:16934628 |
| HIF1A | IL6 | +1 | PMID:20600219 |
| IL6 | MMP13 | +1 | PMID:15601621 |
| ATF3 | IL6 | -1 | PMID:16688168 |
| IL6 | PTGS2 | +1 | PMID:16837651 |
| IL6 | MYC | +1 | PMID:20974848 |
| IFNA1 | IL6 | +1 | PMID:12434062 |
| NOV | MMP1 | +1 | PMID:15611078 |
| FGF2 | THBS1 | +1 | PMID:15927970 |
| FGF2 | ABCB1 | +1 | PMID:17620438 |
| FGF2 | FOS | +1 | PMID:8783257 |
| FGF2 | MMP1 | +1 | PMID:19107653 |
| FGF2 | BCL2 | +1 | PMID:11380405 |
| FGF2 | PTGS2 | +1 | PMID:9920767 |
| FGF2 | VEGFA | +1 | PMID:15485645 |
| FGF2 | PCNA | +1 | PMID:17003443 |
| PTTG1 | FGF2 | +1 | PMID:21858218 |
| IFNA1 | FGF2 | -1 | PMID:9485039 |
| ICAM1 | FOS | +1 | PMID:12097408 |
| IFNA1 | ICAM1 | -1 | PMID:11593644 |
| MAPK8 | ICAM1 | +1 | PMID:15389584 |
| PRKCA | ABCB1 | -1 | PMID:9823967 |
| PTGS2 | ABCB1 | +1 | PMID:17510421 |
| MYCN | ABCB1 | +1 | PMID:12819037 |
| YBX1 | ABCB1 | +1 | PMID:17038319 |
| IFNA1 | ABCB1 | -1 | PMID:17331344 |
| EGFR | PTGS2 | +1 | PMID:15781636 |
| PTGS2 | EGFR | -1 | PMID:19671676 |
| EGFR | NOTCH1 | -1 | PMID:18604200 |
| NOTCH1 | EGFR | +1 | PMID:18359760 |
| EGFR | VEGFA | +1 | PMID:21074412 |
| MUC1 | EGFR | +1 | PMID:16082192 |
| YBX1 | EGFR | +1 | PMID:1967130 |
| IFNA1 | EGFR | +1 | PMID:1718587 |
| PTGS2 | CD44 | +1 | PMID:12393872 |
| VEGFA | CD44 | +1 | PMID:9242547 |
| RAS | CD44 | +1 | PMID:8453616 |
| MYCN | CD44 | -1 | PMID:11035936 |
| PRKCA | HSPA4 | +1 | PMID:17208995 |
| PRKCA | MMP1 | +1 | PMID:16368506 |
| PRKCA | VEGFA | +1 | PMID:1511446 |
| PRKCA | MYC | -1 | PMID:20141613 |
| PRKCA | ERBB2 | +1 | PMID:17545611 |
| PTTG1 | BAX | +1 | PMID:15242522 |
| CIAPIN1 | BAX | -1 | PMID:16410721 |
| TGFA | FOS | +1 | PMID:8783257 |
| TGFA | PTGS2 | +1 | PMID:12930301 |
| TGFA | MYC | +1 | PMID:10839631 |
| IFNA1 | TLR3 | +1 | PMID:16087162 |
| HSPA4 | FOS | -1 | PMID:11189444 |
| PTGS2 | HSPA4 | +1 | PMID:14717913 |
| MYC | HSPA4 | +1 | PMID:1459202 |
| MYC | CDC25A | +1 | PMID:10205150 |
| FOS | MMP1 | +1 | PMID:21344389 |
| FOS | BCL2 | +1 | PMID:19079363 |
| BCL2 | FOS | +1 | PMID:15326476 |
| PTGS2 | FOS | +1 | PMID:16685273 |
| VEGFA | FOS | +1 | PMID:14741347 |
| RAS | FOS | +1 | PMID:7917786 |
| IFNA1 | FOS | +1 | PMID:15970516 |
| CSNK2 | FOS | +1 | PMID:1915270 |
| MAPK8 | FOS | +1 | PMID:17085440 |
| E2F1 | HIC1 | +1 | PMID:19491197 |
| EZH2 | RAD51 | -1 | PMID:16331887 |
| HIF1A | MMP1 | +1 | PMID:17335808 |
| AR | MMP1 | -1 | PMID:8798622 |
| MAPK8 | PSEN1 | +1 | PMID:18374905 |
| E2F1 | SIVA1 | +1 | PMID:15105421 |
| HSP90AB1 | MMP13 | -1 | PMID:18593760 |
| BCL2 | VEGFA | +1 | PMID:12205045 |
| VEGFA | BCL2 | +1 | PMID:11895790 |
| BCL2 | SLC2A1 | +1 | PMID:15120582 |
| MYC | BCL2 | -1 | PMID:11438662 |
| BCL2 | RAS | +1 | PMID:15326476 |
| NCL | BCL2 | +1 | PMID:21048921 |
| CIAPIN1 | BCL2 | -1 | PMID:18059532 |
| POU4F1 | BCL2 | +1 | PMID:9722627 |
| IFNA1 | BCL2 | -1 | PMID:12881711 |
| PTEN | HIF1A | -1 | PMID:18158893 |
| HIF1A | VEGFA | +1 | PMID:17919812 |
| HIF1A | SLC2A1 | +1 | PMID:17387384 |
| HIF1A | PLAUR | +1 | PMID:17335808 |
| HIPK2 | HIF1A | -1 | PMID:19046997 |
| YBX1 | MMP13 | -1 | PMID:17822788 |
| FOXM1 | VEGFA | +1 | PMID:17804744 |
| FOXM1 | CCNB1 | +1 | PMID:11682060 |
| TCF7L2 | MYC | +1 | PMID:9727977 |
| AXIN1 | TCF7L2 | -1 | PMID:17768662 |
| MYC | BRCA1 | +1 | PMID:21668996 |
| POU4F1 | BRCA1 | +1 | PMID:11470235 |
| PTEN | EPHB4 | -1 | PMID:15930280 |
| ERBB2 | CDKN1B | -1 | PMID:16951165 |
| E2F1 | APAF1 | +1 | PMID:18056406 |
| MAPK8 | ATF3 | +1 | PMID:18377912 |
| S100A2 | PTGS2 | -1 | PMID:16908593 |
| PTGS2 | MCL1 | +1 | PMID:16000874 |
| NOTCH1 | PTGS2 | +1 | PMID:19290049 |
| PTGS2 | VEGFA | +1 | PMID:18533784 |
| VEGFA | PTGS2 | +1 | PMID:21273371 |
| NME1 | PTGS2 | +1 | PMID:16415009 |
| IGFBP7 | PTGS2 | -1 | PMID:19374835 |
| ERBB2 | PTGS2 | +1 | PMID:11901151 |
| IFNA1 | PTGS2 | +1 | PMID:16685393 |
| RAS | PTGS2 | +1 | PMID:19903783 |
| PRKD1 | PTGS2 | +1 | PMID:19794144 |
| MAPK9 | PTGS2 | +1 | PMID:9786861 |
| MAPK8 | PTGS2 | +1 | PMID:15546960 |
| E2F1 | MCL1 | -1 | PMID:11857079 |
| VEGFA | DUSP5 | +1 | PMID:19741200 |
| MYC | GADD45A | -1 | PMID:15021909 |
| VEGFA | NOTCH1 | +1 | PMID:12482957 |
| NOTCH1 | CCNA | -1 | PMID:14678992 |
| IFNA1 | FAS | +1 | PMID:14616354 |
| PTEN | VEGFA | -1 | PMID:16527906 |
| PTEN | PCNA | -1 | PMID:17826033 |
| RAS | PTEN | -1 | PMID:19000654 |
| MYC | VEGFA | +1 | PMID:15580293 |
| IGFBP7 | VEGFA | -1 | PMID:19374835 |
| SERPINB5 | VEGFA | -1 | PMID:19374835 |
| HTATIP2 | VEGFA | -1 | PMID:19349353 |
| VEGFA | ID3 | +1 | PMID:15494533 |
| RAS | VEGFA | +1 | PMID:11507052 |
| PRKG1 | CDKN1A | +1 | PMID:18593937 |
| MYC | CDKN1A | -1 | PMID:12408820 |
| RAS | CDKN1A | +1 | PMID:19440234 |
| E2F1 | AR | -1 | PMID:17178887 |
| NME1 | MYC | +1 | PMID:19170058 |
| MYC | NME1 | +1 | PMID:11960382 |
| MYC | TNFRSF10A | +1 | PMID:17881904 |
| MYCN | MYC | -1 | PMID:7529553 |
| HTATIP2 | MYC | -1 | PMID:15073177 |
| PTTG1 | MYC | +1 | PMID:11115508 |
| MYC | E2F1 | +1 | PMID:17784791 |
| IFNA1 | MYC | -1 | PMID:11798827 |
| IFNA1 | MYC | +1 | PMID:10068671 |
| CHEK2 | MYC | +1 | PMID:19812253 |
| MYC | CDK2 | +1 | PMID:8157956 |
| E2F1 | PCNA | +1 | PMID:12468739 |
| KLF4 | CCNB1 | -1 | PMID:14627709 |
| IFNA1 | TNFRSF10B | +1 | PMID:12642868 |
| E2F1 | MYCN | +1 | PMID:14645238 |
| IFNA1 | E2F1 | -1 | PMID:10208422 |
| E2F1 | CHEK2 | +1 | PMID:15024084 |
| HIPK2 | POU4F1 | -1 | PMID:15492043 |
| IFNA1 | EIF2AK2 | +1 | PMID:15254208 |
| RAS | CCNA | +1 | PMID:15737994 |
| MAPK1 | P53 | +1 | PMID:11409876 |
| VRK1 | P53 | +1 | PMID:15542844 |
| RAF1 | P53 | +1 | PMID:10732786 |
| CSNK2 | P53 | +1 | PMID:9244359 |
| HIPK2 | P53 | +1 | PMID:16601678 |
| CDK2 | P53 | +1 | PMID:11078726 |
| CHEK1 | P53 | +1 | PMID:10673501 |
| HIPK4 | P53 | +1 | PMID:18022393 |
| CDK5 | P53 | +1 | PMID:17591690 |
| PPM1D | P53 | -1 | PMID:15870257 |
| AURKA | P53 | -1 | PMID:14702041 |
| PRKD1 | P53 | +1 | PMID:12628923 |
| DYRK2 | P53 | +1 | PMID:17349958 |
| MAPK9 | P53 | +1 | PMID:17525747 |
| CDK9 | P53 | +1 | PMID:16741955 |
| MAPK8 | P53 | +1 | PMID:11057897 |
| EIF2AK2 | P53 | +1 | PMID:19210572 |
| ATM | P53 | +1 | PMID:10608806 |
| CSNK2 | MDM2 | +1 | PMID:16335531 |
| CSNK2 | MDM2 | -1 | PMID:21769452 |
| EGFR | ESR1 | +1 | PMID:19470835 |
| PRKCA | ABCB1 | +1 | PMID:15563462 |
| MAPK1 | NR2C1 | +1 | PMID:18682553 |
| CDC25A | EGFR | -1 | PMID:11912208 |
| CHEK1 | CDC25A | -1 | PMID:12759351 |
| PPM1D | CHEK1 | -1 | PMID:15870257 |
| IFNA1 | PRKCA | +1 | PMID:17988665 |
| PRKCA | RAF1 | +1 | PMID:8321321 |
| MAPK8 | BAX | +1 | PMID:16709574 |
| CHEK2 | CDC25A | -1 | PMID:12759351 |
| CCNA | CDC25A | +1 | PMID:10926775 |
| CDK2 | CDC25A | +1 | PMID:10926775 |
| CCNA | FEN1 | -1 | PMID:12853968 |
| MAPK8 | BCL2 | +1 | PMID:11323415 |
| CSNK2 | TCF7L2 | +1 | PMID:11711551 |
| CHEK2 | BRCA1 | +1 | PMID:14701743 |
| CDK2 | CDKN1B | -1 | PMID:8622855 |
| DUSP5 | MAPK1 | -1 | PMID:8221888 |
| CSNK2 | PTEN | +1 | PMID:11035045 |
| CSNK2 | MYC | +1 | PMID:2663470 |
| CDK5 | MYC | +1 | PMID:18408012 |
| CDK5 | ERBB2 | +1 | PMID:16203963 |
| CSNK2 | MYCN | +1 | PMID:1425701 |
| CSNK2 | NCL | +1 | PMID:3190709 |
| CHEK2 | E2F1 | +1 | PMID:12717439 |
| CCNA | E2F1 | -1 | PMID:7838523 |
| CDK2 | E2F1 | +1 | PMID:7969176 |
| MAPK8 | E2F1 | -1 | PMID:10075927 |
| MAPK1 | YBX1 | +1 | PMID:16198352 |
| CSNK2 | HDAC1 | +1 | PMID:11602581 |
| ATM | CHEK2 | +1 | PMID:16936775 |
| CCNA | AXIN1 | +1 | PMID:15063782 |
| CDK2 | AXIN1 | +1 | PMID:15063782 |
| CDK2 | CCNA | +1 | PMID:10652300 |
| FHL2 | MAPK1 | -1 | PMID:14729955 |
| MAPK1 | RAF1 | +1 | PMID:1730637 |
| BCL6 | CDKN1B | -1 | PMID:10981963 |
| CDKN1B | BCL2 | -1 | PMID:14676836 |
| HF1A | CDKN1B | +1 | PMID:19342889 |
| PTGS2 | CDKN1B | -1 | PMID:14587561 |
| PRKG1 | CDKN1B | +1 | PMID:18593937 |
| MYCN | CDKN1B | -1 | PMID:18198336 |
| CDK5 | CDKN1B | +1 | PMID:16341208 |
| CDKN1B | CCNA | -1 | PMID:8547220 |
| RAS | CDKN1B | +1 | PMID:14504289 |
| ATR | P53 | +1 | PMID:16557269 |
| ATR | CHEK1 | +1 | PMID:16557269 |
| PRKDC | P53 | +1 | PMID:11042698 |
| MDM4 | MDM2 | +1 | PMID:16557269 |
| MDM4 | P53 | -1 | PMID:16557269 |
| MDM2 | MDM4 | -1 | PMID:16557269 |
| P53 | C12orf5 | +1 | PMID:16557269 |
| P53 | PERP | +1 | PMID:16557269 |
| P53 | SIAH1 | +1 | PMID:16557269 |
| P53 | LRDD | +1 | PMID:16557269 |
| P53 | P53AIP1 | +1 | PMID:16557269 |
| P53 | SESN2 | +1 | PMID:16557269 |
| P53 | SFN | +1 | PMID:16557269 |
| CDKN1B | CDK2 | -1 | PMID:18354415 |
| CDKN1A | CDK2 | -1 | PMID:8756624 |
| CDKN2A | MDM2 | -1 | PMID:20523835 |
| P53 | CDKN2A | -1 | PMID:9774662 |
| CDK2 | MDM2 | -1 | PMID:21278451 |
| GAPDH | SIAH1 | +1 | PMID:15951807 |
| CHEK1 | MDM4 | -1 | PMID:16511572 |
| SFN | MMP1 | +1 | PMID:19533306 |
| SIAH1 | HIF1A | +1 | PMID:15210114 |
| SFN | CCNB1 | -1 | PMID:17573669 |
| E2F1 | SIAH1 | +1 | PMID:20187294 |
| CDKN2A | MDM4 | +1 | PMID:15907800 |
| SFN | CDK2 | -1 | PMID:10767298 |
| PPM1D | MDM4 | +1 | PMID:19808970 |
| DYRK2 | P53AIP1 | +1 | PMID:17349958 |
| ATM | ATR | +1 | PMID:17088261 |
| SFN | CDKN1B | +1 | PMID:20642839 |
| RAS | RAF1 | +1 | PMID:9020159 |
| RAS | MAPK1 | +1 | PMID:9020159 |
| E2F1 | EZH2 | +1 | PMID:19893569 |
| TFDP1 | EZH2 | +1 | PMID:20565746 |
| ATM | AATF | +1 | PMID:17157788 |
| ATR | AATF | +1 | PMID:17157788 |
| CHEK2 | AATF | +1 | PMID:17157788 |
| ATM | BCL6 | -1 | PMID:18346918 |
| P53 | ZMAT3 | +1 | PMID:19805223 |
| P53 | KLF4 | +1 | PMID:19826046 |
| ATM | BRCA1 | +1 | PMID:12082091 |
| ATR | BRCA1 | +1 | PMID:12082091 |
| MAPK14 | SGK | +1 | PMID:12488318 |
| P53 | PPM1D | +1 | PMID:18265945 |
| P53 | PPM1D | -1 | PMID:20093361 |
| PPM1D | MAPK14 | -1 | PMID:18265945 |
| MAPK14 | P53 | +1 | PMID:18265945 |
| ATM | DYRK2 | +1 | PMID:19965871 |
| MDM2 | DYRK2 | -1 | PMID:19965871 |
| ATM | MDM2 | -1 | PMID:16943424 |
| ATR | MDM2 | -1 | PMID:16943424 |
| ATM | MDM4 | -1 | PMID:16943424 |
| CHEK2 | MDM4 | -1 | PMID:16943424 |
| IGF1R | RAS | +1 | PMID:19174523 |
| PRKG1 | MAPK14 | -1 | PMID:16990590 |

**Table S2: Incorrect interactions not retained in the PKT206 model.**

Column 1 shows the source node of the interaction; column 2 shows the target node of the interaction; column 3 shows the interaction type where -1 means inhibition and +1 means activation; column 4 shows the error type and reason for not retaining the interaction; column 5 shows the PubMed identifier of the references used.

| Node A | Node B | Interaction type | Reason | References |
| --- | --- | --- | --- | --- |
| NFKBIA | P53 | +1 | Only binding interaction found in STRING v9.0 |  |
| IL4 | P53 | +1 | Wrong target recognition and a general term appears in complex context (B cell activation by DC40L and IL-4 induced p53 expression without any adverse effect on cell cycle progression). | PMID:10211969 |
| ATIC | P53 | +1 | Mistake in STRING v8.3, ATIC is a compound and cell specific in the human epithelial cerical cancer cell line CaSki. No interaction found in STRING v9.0 |  |
| ENSG00000173342 | P53 | +1 | ENSG00000173372 was not found in STRING v9.0 |  |
| CAPNS1 | P53 | +1 | No interaction found in STRING v9.0. |  |
| ZNF331 | P53 | +1 | Wrong target recognition and a general term appears in complex context (RITA activates the transcription of proapoptotic p53 targets, Noxa, PUMA, and BAX.) | PMID:20395210 |
| RBMX2 | P53 | +1 | Wrong recognition of target genes, Hur was not RBMX2 in the abstract shown in STRING v8.3; ELAVL1 activates p53 in the model. |  |
| MDM2 | P53 | +1 | Wrong interaction type recognition in STRING v8.3 and this activation was removed in STRING v9.0. |  |
| TOPBP1 | P53 | +1 | Negation and negative words not recognized by text mining in STRING v8.3 (depletion of TOPBP1 upregulates p53 target genes) and no interaction found in STRING v9.0. | PMID:19289498 |
| LRP6 | P53 | +1 | No interaction found in STRING v9.0 |  |
| ELA2 | P53 | +1 | Wrong gene name recognition, ELA2 is ELANE in PubMed. ELA2 and 4-Hydroxynonenal in evidence are not the same gene (HNE increased p53) | PMID:16632118 |
| POLD3 | P53 | +1 | Wrong gene name recognition in STRING v8.3, the correct interaction was that DDX5 activates p53. Low confidence score for inhibition in STRING v9.0. |  |
| P53 | CD9 | -1 | Wrong gene name recognition, (in the evidence shown in STRING v9.0, it was MRP1 but not CD9, MRP1 in PubMed was ABCC1) (in addition, transfection with antisense -53 oligonucleotides greatly suppressed MRP1 expression and reversed DOX resistance in p53 - R175H cells) | PMID:12647018 |
| P53 | HAMP | +1 | No literature evidence shown in STRING v9.0 and no evidence found in PubMed. |  |
| P53 | HDAC5 | +1 | Negation and negative words not recognized by text mining (in the abstract in STRING v8.3: “nor was the expression of HDAC5 mRNA induced by p53-activating agents”). No interaction link found in STRING v9.0. | PMID:12019172 |
| P53 | NANOG | -1 | Wrong target recognition and a general term appears in complex context in STRING v8.3: "p53 binds to the promoter of Nanog, a gene required for ESC self-renewal, and supresses Nanog expression after DNA damage" | PMID:15619621 |
| P53 | CXCR4 | +1 | Negation and negative words not recognized by text mining ("Short-interfering RNA-mediated depletion of p53 increased endogenous CXCR4 expression in MCF-7 breast cancer cells that contains wild-type p53.") | PMID:17130833 |
| P53 | GDF15 | +1 | Speculations or questions rather than a conclusion (The findings reported here indicate that gamma radiation activates p53 and then increases NAG1, but no clear relationship between p53 and NAG1). | PMID:16435911 |
| P53 | CDK2 | +1 | Negation and negative words not recognized by text mining  (our results establish that the I3C induced G1 arrest of human prostate cancer cells requires the induced production of the activated phosphorylated forms of p53, which stimulate transcription of the CDK2 inhibitor p21.) | PMID:16970927 |
| P53 | TMEFF2 | -1 | Wrong gene name recognition in STRING v8.3; p53 inhibits NR2C1 in the model; no interaction found in STRING v9.0 |  |
| P53 | BAX | -1 | Wrong target recognition and a general term appears in complex context (p38 siRNA also decreases the binding of p53 to the bax promoter and interferes with the association of p53 and STAT1). | PMID:20567883 |
| P53 | SHH | +1 | Wrong gene name recognition and a general term appears in complex context; negation and negative words not recognized by text mining in STRING v8.3 (“We showed that p63 and p73 but not p53 overexpression induces Shh expression”). No interaction found in STRING v9.0. | PMID:17050669 |
| P53 | NUDT6 | -1 | Wrong gene name recognition in STRING v8.3 (NUDT6 was duplicate with FGF2) and no interaction found in STRING v9.0. |  |
| P53 | TGFBI | +1 | No interaction found in STRING v9.0. |  |
| P53 | CDC20 | +1 | Negation and negative words not recognized by text mining  (“small interference RNA (siRNA)–mediated silencing of p53 induce CDC20 expression in normal human dermal fibroblast cells”). | PMID:17873905 |
| P53 | BCL2 | +1 | Wrong target recognition and a general term appears in complex context (decreased expression of pro-apoptotic proteins, p53 and Bax, enhanced expression of cytoprotective protein, Bcl-2, and activation of the cell survival kinase, Akt). | PMID:12071510 |
| P53 | SOD2 | -1 | No literature evidence shown in STRING v9.0 |  |
| P53 | TP53I3 | +1 | Activation at low confidence score in STRING v9.0 and no literature evidence shown for this inhibition in STRING v9.0 |  |
| P53 | ANXA2 | +1 | No literature evidence shown for this inhibition in STRING v9.0 |  |
| P53 | TOP3B | -1 | No interaction shown for this inhibition in STRING v9.0 |  |
| P53 | IFI27 | +1 | Wrong gene name recognition; p53 inhibits CDKN1B in the model. |  |
| P53 | BAK1 | -1 | Negation and negative words not recognized and a general term appears in complex context (Blocking p53 inhibited HCMV-stimulated Bax and Bak expression as well as caspase-3 activation and blocking the ATM pathway inhibited HCMV-stimulated p53 activation). | PMID:15105295 |
| P53 | CDKN2A | +1 | No literature evidence found for this activation in STRING v9.0. |  |
| P53 | LEFTY2 | +1 | Wrong target recognition and a general term appears in complex context (transduction of p53 induces LEFTY2). |  |
| P53 | BNIP3 | +1 | Negation and negative words not recognized by text mining  (Overexpression of HIF1alpha, but not p53, induces the expression of BNIP3). | PMID:11550088 |
| P53 | STMN3 | -1 | A general term appears in complex context and the inhibition was at low confidence score in STRING v9.0 (we demonstrated that p53 negatively regulates stathmin expression through Egr1 pathway). | PMID:19786090 |
| P53 | LPPR4 | +1 | Wrong gene name recognition (LPPR4 replaced with IER3). | PMID:9627114 |
| P53 | NCOA4 | -1 | Wrong gene name recognition (NCOA4 are 4 different genes in mice). |  |
| P53 | LPPR1 | +1 | Wrong gene name recognition in STRING v8.3 (replace LPPR1 with AIFM2). |  |
| P53 | PLEKHG5 | +1 | No interaction found in STRING v9.0. |  |
| P53 | CKAP2 | +1 | No interaction found in STRING v9.0. |  |
| P53 | KCNQ1 | +1 | No interaction found in STRING v9.0. |  |
| P53 | POMC | +1 | No interaction found in STRING v9.0. |  |
| P53 | ALB | -1 | No interaction found in STRING v9.0. |  |
| P53 | LITAF | +1 | No activation evidence shown in STRING v9.0. |  |
| P53 | CHEK2 | -1 | A general term appears in complex context (These results suggest that p53 negatively regulates Chk2 gene transcription through modulation of NF-Y function). | PMID:15044452 |
| ERBB2 | P53 | +1 | Wrong target recognition and a general term appears in complex context (We showed that NDF/HRG and antibodies to erbB-2 receptors up-regulate expression of p53 by stabilizing the protein). | PMID:8700512 |
| ACE | P53 | -1 | No interaction found in STRING v9.0. |  |
| CCL16 | P53 | +1 | No literature evidence shown in STRING v9.0. |  |
| NPM1 | P53 | +1 | Wrong target recognition and a general term appears in complex context (NPM1 activates p53 target genes p21 and Bax). |  |
| CKAP4 | P53 | +1 | No interaction found in STRING v9.0. |  |
| BCKDHB | P53 | -1 | Wrong target recognition and a general term appears in complex context (Here we show that E1B strongly suppresses the expression of p53 target gene such as p21 and Puma-alpha in normal growth conditions). | PMID:17209038 |
| TCHP | P53 | +1 | No interaction found in STRING v9.0. |  |
| MAF | P53 | +1 | No interaction found in STRING v9.0. |  |
| EP400 | P53 | -1 | Wrong target recognition and a general term appears in complex context (These data suggest that the p400 complex inhibits p53--p21 transcription). | PMID:15655109 |
| HEPH | P53 | +1 | Wrong gene name recognition in STRING v 8.3, CFT and CPL increased the expression of p53 in the evidence, and CPL (cephalothin) is not HEPH (hephaestin) in PubMed. |  |
| BCKDHA | P53 | +1 | No interaction found in STRING v9.0. |  |
| NTRK1 | P53 | +1 | Wrong target recognition and a general term appears in complex context (TrKA (NTRK1) increased the expression of p53 target proteins). | PMID:15961390 |
| PARK2 | P53 | +1 | In STRING v9.0, PARK2 and p53 only co-mentioned in abstract. |  |
| PTGS2 | P53 | +1 | Speculations or questions rather than a conclusion (It was concluded that COX-2 can increase the expression of p53 protein). | PMID:19513627 |
| CD40LG | P53 | +1 | Wrong target recognition and a general term appears in complex context (B cell activation by CD40L and IL-4 induced p53 expression without any adverse effect on cell cycle progression). | PMID:10211969 |
| DST | P53 | -1 | No interaction found in STRING 9.0. |  |
| HOOK1 | P53 | +1 | No interaction found in STRING 9.0. |  |
| ANKRD1 | P53 | +1 | No interaction found in STRING 9.0. |  |
| DAXX | P53 | -1 | No interaction found in STRING 9.0. |  |
| WAS | P53 | +1 | No interaction found in STRING 9.0. |  |
| MYC | P53 | -1 | Negation and negative words not recognized by text mining (not mutant p53 reduced markedly transcription from the c-myc promoter). | PMID:8479742 |
| PGA4 | P53 | -1 | Wrong gene name recognition (p53 expression was abolished by pepsin predigestion and PGA4 is pepsinogen 5). | PMID:8008749 |
| CD68 | P53 | +1 | Only co-mentioned in PubMed abstracts in STRING v9.0. |  |
| AXIN1 | P53 | -1 | Negation and negative words not recognized by text mining (Silencing of Axin reduces p53 expression). | PMID:19513548 |
| ENSG00000206206 | P53 | -1 | Interaction record change to binding at low confidence level. |  |
| ENSG00000206279 | P53 | -1 | No functional link found in STRING v9.0. |  |
| ENSG00000206439 | P53 | +1 | No functional link found in STRING v9.0. |  |
| CDK7 | P53 | +1 | Wrong target recognition and a general term appears in complex context (p53 is phosphorylated by CDK7-cyclin H in a p36MAT1-dependent manner). | PMID:9372954 |
| JUN | P53 | +1 | Incorrect evidence according to the abstract in STRING v8.3. | PMID:12930303 |
| P53 | MAPK8 | +1 | Incorrect evidence according to the abstract in STRING v8.3. | PMID:17406061 |
| PPM1D | P53 | +1 | Incorrect (PPM1D dephosphorylates Chk1 and p53). | PMID:15870257 |
| H2AFX | P53 | +1 | Incorrect evidence according to the abstract in STRING v8.3. | PMID:19328784 |
| TNK2 | P53 | +1 | No functional link found in STRING v9.0. |  |
| TGFB1 | MMP2 | +1 | Correct, but cell specific, (TGFB1 upregulate MMP2 in some cell lines). | PMID:7616276 |
| MMP2 | TGFB1 | +1 | Cell specific (TGFB1 upregulate MMP2 in some cell lines (cell type specific)). | PMID:7616276 |
| TGFB1 | CCND1 | -1 | Cell specific (TGFB1 inhibits cyclin D1 expression in intestinal epithelial cells). | PMID:7824270 |
| TGFB1 | CCND1 | +1 | Correct, but cell specific (TGFB1 inhibits cyclin D1 expression in intestinal epithelial cells). | PMID:7824270 |
| IL6 | TGFB1 | +1 | In STRING v9.0, only co-mentioned in PubMed abstract. | PMID:8528940 |
| TGFA | TGFB1 | +1 | In STRING v9.0, only co-mentioned in PubMed abstract. | PMID:1639863 |
| TGFB1 | PTGS2 | -1 | Wrong interaction type recognition (TGFB1 induce PTGS2). | PMID:10935498 |
| E2F1 | TGFB1 | +1 | In STRING v9.0, only co-mentioned in PubMed abstract and at low confidence level. |  |
| ATIC | SP7 | -1 | No functional link found in STRING v9.0. |  |
| ATIC | VEGFA | +1 | Wrong gene name recognition, ATIC is a compound. | PMID:16516166 |
| ATIC | MYC | +1 | Wrong gene name recognition, ATIC is a compound and no functional link found in STRING v9.0. |  |
| ATIC | SLC2A4 | +1 | Wrong gene name recognition, ATIC is a compound. | PMID:11509501 |
| ATIC | KLF4 | -1 | Wrong gene name recognition, ATIC is a compound and no functional link found in STRING v9.0. |  |
| MDM2 | ABCB1 | -1 | No functional link found between MDM2 and ABCB1 in STRING v9.0. |  |
| MDM2 | CCNA1 | +1 | Incorrect, in STRING v9.0, only co-mentioned in PubMed abstract. | PMID:11739724 |
| MDM2 | CCNA1 | -1 | Incorrect, in STRING v9.0, only co-mentioned in PubMed abstract. | PMID:16901994 |
| MDM2 | CCNA2 | +1 | In STRING v9.0, only co-mentioned in PubMed abstract. | PMID:16901994 |
| MDM2 | CCNA2 | -1 | In STRING v9.0, only co-mentioned in PubMed abstract. | PMID:19808975 |
| ELAVL1 | BCL2 | +1 | No evidence found in STRING v8.3. |  |
| ELAVL1 | IFI27 | -1 | Wrong gene name and interaction type recognition, replace IFI27 with CDKN1B and ELAVL1 inhibits CDKN1B. | PMID:12464637 |
| CD9 | MMP2 | +1 | Correct but indirectly, (CD9 induces MMP-2 by activating p38MAPK, JNK and c-Jun pathways). | PMID:16000878 |
| MYCN | CD9 | +1 | Wrong gene name recognition. CD9 replaced with ABCC1. | PMID:14737110 |
| ESR1 | CCND1 | -1 | Negation and negative words not recognized by text mining (ESR1 downregulators decrease CCND1 expression). | PMID:15544931 |
| ESR1 | EGFR | +1 | Negation and negative words not recognized by text mining (antisense ESR1 increases EGFR). | PMID:9269899 |
| ESR1 | VEGFA | +1 | Wrong interaction type recognition and the effect is not clear (ESR1 bind to VEGF promoter). | PMID:17272396 |
| AR | ESR1 | +1 | Negation and negative words not recognized by text mining (reduced AR expression and./or increased ESR1 expression in specific brain areas) | PMID:19073212 |
| ESR1 | MYC | -1 | Wrong interaction recognition (the expression of ER-regulated genes c-myc and tff1, which was blocked by ESR1) | PMID:19661132 |
| HIF1A | MMP2 | +1 | Negation and negative words not recognized by text mining (HIF1A over expression could increase expression of uPAR and MMP1, but not MMP2). | PMID:17335808 |
| FOXM1 | MMP2 | -1 | Negation and negative words not recognized by text mining (blockade of FoxM1 expression suppressed MMP-2 expression). | PMID:17404569 |
| PTGS2 | MMP2 | -1 | In STRING v9.0, only co-mentioned in PubMed abstract. | PMID:12898179 |
| MYC | MMP2 | -1 | Negation and negative words not recognized by text mining (Silenced c-Myc led to reduce MMP-2 gene expression). | PMID:19258038 |
| RASD1 | MMP2 | -1 | Wrong recognition of gene name, RASD1 should be RAS, and no functional link between RASD1 and MMP2 in STRING v9.0. HRAS activates MMP2 indirectly. | PMID:15677464 |
| RASD1 | CKM | -1 | Wrong recognition of gene name, RASD1 should be RAS, and no functional link between RASD1 and CKM in STRING v9.0 |  |
| CCND1 | BRCA1 | +1 | Wrong target recognition and a general term appears in complex context (BRCA1-IRIS activates Cyclin D1 expression). | PMID:17278098 |
| IFI27 | CCND1 | -1 | Wrong gene name recognition, IFI27 is not p27, | PMID:11940657 |
| MAPK1 | CCND1 | -1 | Incorrect interaction type (GL331’s inhibition of cyclinD1 promoter was attenuated by ectopic Erk-2 expression). | PMID:11562439 |
| IL6 | ICAM1 | +1 | A general term appears in complex context (IL-6 induces polarized expression of ICAM-1) | PMID:16428777 |
| IL6 | ICAM1 | -1 | A general term appears in complex context (TGF-beta mediated inhibition of IL-6 induced ICAM-1 expression) | PMID:14500551 |
| IL6 | MMP1 | -1 | Negation and negative words not recognized by text mining and a general term appears in complex context (The specific inhibition of IL-6 using a monoclonal antibody against IL-6 greatly reduced the expression of MMP-1). | PMID:15610507 |
| IL6 | BCL3 | -1 | A general term appears in complex context (BCL3 is induced by IL-6 via Stat3 binding to intronic enhancer HS4). | PMID:16732314 |
| PTGS2 | IL6 | +1 | Wrong targets recognition, a general term appears in complex context, (COX-2 derived PGs upregulate COX-2 and IL-6 expression). | PMID:8647962 |
| IL6 | MCL1 | +1 | Wrong targets recognition and a general term appears in complex context (culture of MM.1S cell in IL-6 did induce Mcl-1 expression). | PMID:12660820 |
| IL6 | VEGFA | -1 | Wrong targets recognition and a general term appears in complex context (macrophage-associated cytokines (interleukin-1 beta, interleukin-6 and tumor necrosis factor-alpha) inhibited VEGF mRNA expression). | PMID:11423572 |
| PRKG1 | IL6 | +1 | Wrong targets recognition and a general term appears in complex context (Insulin-induced IL-6 gene expression is mediated by cGMP/cyclic GMP-dependent protein kinase/cAMP response element binding protein). | PMID:18617614 |
| FGF2 | NOTCH1 | +1 | Negation and negative words not recognized by text mining (vascular endothelial growth factor (VEGF) but not basic fibroblast growth factor can induce gene expression of Notch1). | PMID:12482957 |
| FOS | ICAM1 | -1 | Wrong relationship (The inhibitory effect of quercetin on ICAM-1 expression was mediated by sequential attenuation of the c-fos and c-jun mRNA expressions). | PMID:18982426 |
| CDK2 | ICAM1 | -1 | No evidence shown in STRING v9.0. |  |
| YBX1 | ABCB1 | -1 | Negation and negative words not recognized by text mining (Transfection of YB-1 antisense oligonucleotides inhibited P-glycoprotein expression induced by fragmented hyaluronan). | PMID:17038319 |
| RASD1 | ABCB1 | -1 | No functional link shown in STRING v9.0. |  |
| EGFR | BCL2 | +1 | Wrong targets recognition and a general term appears in complex context (Immunostained cells treated with oligos directed against mRNA encoding TGF-alpha (MR-1) either alone or in combination with that directed against EGFR (MR-2) had increased bcl-2 expression (+3 to +5). | PMID:9232607 |
| EGFR | VEGFA | -1 | Negation and negative words not recognized by text mining (EGFR tyrosine kinase inhibitors decrease VEGF expression). | PMID:16540671 |
| PRKCA | MMP1 | -1 | No interaction found in STRING v9.0. |  |
| BAX | BCL2 | +1 | Wrong targets recognition and a general term appears in complex context (DAPT treatment can ameliorate the severity of tubular damage after renal IRI, lower the expression of NF-κB2,MCP-1 and bax protein, increase the expression of bcl-2 protein). | PMID:21332343 |
| BAX | BCL2 | -1 | A general term appears in complex context (but the content of MDA, myocardial AI and the expression of Bax protein were decreased obviously and the expression of Bcl-2 protein was up-regulated in EP group(P<0.05)). | PMID:18563323 |
| BCL2 | BAX | -1 | A general term appears in complex context (the expression of bcl-2 mRNA decreased gradually and the expression of bax increased gradually). | PMID:16129036 |
| BAX | BAK1 | -1 | Wrong targets recognition and a general term appears in complex context (“we show that human papillomavirus E7-loaded dendritic cells transfected with BAK/BAX siRNA downregulate Bak and Bax protein expression”). | PMID:15916483 |
| BAK1 | BAX | -1 | Wrong targets recognition and a general term appears in complex context (“we show that human papillomavirus E7-loaded dendritic cells transfected with BAK/BAX siRNA downregulate Bak and Bax protein expression”). | PMID:15916483 |
| TGFA | PTGS2 | -1 | Wrong entity recognition (Although a neutralizing antibody to transforming growth factor-alpha (TGF-alpha) suppressed COX-2 expression induced by TGF-alpha). | PMID:12930301 |
| BCL2 | FOS | +1 | Wrong targets recognition and a general term appears in complex context (Here we report that the immediate early gene (IEG) c-Fos can stimulate the expression of Bcl-2 depending on a specific AP-1 – binding site in the Bcl-2 promoter). | PMID:19079363 |
| FOS | VEGFA | -1 | A general term appears in complex context (These findings show for the first time that (VEGF and PLGF induce mRNA expression of the transcription factors FosB and C-Fos). | PMID:14741347 |
| IFNA1 | FOS | -1 | Negation and negative words not recognized by text mining (neither the IL-3 – induced DNA binding of STAT5 nor the transcription of the STAT5 – dependent genes oncostain-M, pim-1 and c-fos were suppressed by IFN-alpha). | PMID:10574332 |
| RASD1 | FOS | +1 | No interaction link found in STRING v9.0. |  |
| MAPK8 | FOS | -1 | A general term appears in complex context (Inhibiting low substratum rigidity-induced JNK activation prevented aberrant c-Fos expression). | PMID:17085440 |
| VEGFA | MMP1 | +1 | A general term appears in complex context (VEGF stimulated the expression of transcription factor Ets-1 as well as matrix metalloproteinase-1(MMP-1) and Flt-1 in HUVECs). | PMID:10865839 |
| VEGFA | MMP1 | -1 | A general term appears in complex context (Ribozyme mediated suppression of vascular endothelial growth factor gene expression enhances matrix metalloproteinase-1 expression in a human hepatocellular carcinoma cell line). | PMID:12063553 |
| MAPK1 | PSEN1 | -1 | No interaction found in STRING v9.0. |  |
| BAK1 | BCL2 | +1 | A general term appears in complex context (The results demonstrated a tendency for stronger and more frequent expressions of c-myc, Bak and Bax despite a rather weaker expression of Bcl-2 in cancer tissues from the elderly compared with those from the younger patients). | PMID:12559416 |
| PTGS2 | BCL2 | +1 | Negation and negative words not recognized by text mining (we did not observe any changes in Bcl-2, Bcl-XL, or Bax expression induced by COX-2 or PGE2). | PMID:16127422 |
| BCL2 | PTGS2 | +1 | No literature evidence found for this inhibition in STRING v9.0 |  |
| MYC | BCL2 | +1 | A general term appears in complex context (The results demonstrated a tendency for stronger and more frequent expressions of c-myc, Bak and Bax despite a rather weaker expression of Bcl-2 in cancer tissues from the elderly compared with those from the younger patients). | PMID:12559416 |
| RAF1 | BCL2 | -1 | Only binding reaction found in STRING v9.0 at low confidence score 0.620. |  |
| BCL2 | RAF1 | -1 | Only binding reaction found in STRING v9.0 at low confidence score 0.620. |  |
| HIF1A | VEGFA | -1 | No literature evidence found for this inhibition in STRING v9.0. |  |
| FOXM1 | ERBB2 | +1 | Only expression interaction found in STRING v9.0 (“Our results indicate that the HER2 receptor regulates the expression of FoxM1 transcription factor”). | PMID:19513552 |
| CCNG1 | CCNB1 | +1 | Wrong relationship recognized (cyclin G1 regulated transcription of cyclin B1 in a p53 – independent manner). | PMID:16322753 |
| BRCA1 | GADD45A | +1 | Only functional link found in STRING v9.0. |  |
| BRCA1 | GADD45A | -1 | Only functional link found in STRING v9.0. |  |
| POU4F1 | BRCA1 | -1 | Speculations or questions rather than a conclusion. (Consistent with the possibility that Brn-3a may regulate expression of BRCA-1 in the testis). | PMID:11470235 |
| MAPK8 | ATF3 | -1 | Wrong target recognition and a general term appears in complex context (only ECs pretreated with a specific inhibitor to JNK suppressed the hypoxia-induced ATF3 expression). | PMID:18377912 |
| PTGS2 | TNFRSF10B | +1 | Wrong target recognition and a general term appears in complex context (Celecoxib and a novel COX-2 inhibitor ON09310 upregulate death receptor 5 expression via GADD153/CHOP). | PMID:17968315 |
| RASD1 | PTGS2 | -1 | No interactions found in STRING v9.0. |  |
| RAF1 | PTGS2 | -1 | Wrong interaction recognition and a general term appears in complex context (YC-1 – induced COX-2 expression was attenuated by a Raf-1 inhibitor (GW 5074) in a concentration-dependent manner). | PMID:19717011 |
| MAPK9 | PTGS2 | -1 | Negation and negative words not recognized by text mining (The current study demonstrates that overexpression of the dominant negative form of JNK1 or p54JNK2/SAPKbeta reduces Cox-2 expression). | PMID:9786861 |
| MAPK8 | PTGS2 | +1 | Wrong target recognition and a general term appears in complex context (Inhibition of arsenite – induced ERK or JNK signaling using a pharmacologic inhibitor of ERK or JNK substantially blocked COX-2 expression). | PMID:19808956 |
| RASD1 | FAS | -1 | No literature evidence found for this inhibition in STRING v9.0. |  |
| SLC2A4 | SLC2A1 | +1 | No interaction found in STRING v9.0. |  |
| MYC | PCNA | -1 | No interaction found in STRING v9.0. |  |
| PCNA | MYC | -1 | No interaction found in STRING v9.0. |  |
| MYCN | MYC | +1 | No interaction found in STRING v9.0. |  |
| MYC | E2F1 | -1 | Wrong target recognition and a general term appears in complex context (The induction of the miR – 17 cluster by Myc attenuates E2F1 protein expression). | PMID:19806017 |
| IFNA1 | MYC | +1 | Wrong target recognition and a general term appears in complex context (Treatment of T lymphocytes with IFN-alpha, IL-2,IL-12, and IL-15 upregulated IL-2Ralpha, c-myc, and pim-1 gene expression). | PMID:10068671 |
| CHEK2 | MYC | -1 | Negation and negative words not recognized by text mining (Hur silencing or Chk2 silencing reduced c-Myc translation and c-Myc expression levels). | PMID:19812253 |
| MYC | CDK2 | +1 | Negation and negative words not recognized by text mining (The presence of antisense c-myc oligonucleotide inhibited the expression of cdc2 and cdk2 without affecting the expression of IL-2R alpha and blocked the activated T cells in the G1 phase). | PMID:8157956 |
| RAF1 | CDK4 | +1 | No interactions found in STRING v9.0 |  |
| CDK4 | CDK2 | +1 | Wrong interaction recognition and a general term appears in complex context (Although TCDD treatment leads to a strong increase in cyclin D2 / cdk4 and cyclin A / cdk2 complex formation). | PMID:12387751 |
| TGFA | EGFR | +1 | Wrong interaction recognition, only binding link found and no literature evidence for Homo sapiens. |  |
| CDC25A | EGFR | +1 | Wrong interaction recognition (Both purified GST – Cdc25A protein and endogenous Hep38 cellular Cdc25A dephosphorylated tyrosine-phosphorylated EGFR). | PMID:11912208 |
| PPM1D | CHEK1 | +1 | Wrong interaction recognition (PPM1D dephosphorylates Chk1 and p53 and abrogates cell cycle checkpoints). | PMID:15870257 |
| BAX | MAPK9 | +1 | Only functional link found in STRING v9.0 and those two genes were co-mentioned in abstracts. |  |
| BAX | MAPK8 | +1 | Wrong target recognition and a general term appears in complex context (Obesity increased the glomerular area and was associated with activation of the UPR in renal cells with a greater abundance of glucose-related protein 78, C/EBP homologous protein, Bax, phosphorylated c-Jun amino-terminal kinase). | PMID:19414648 |
| CCNA2 | FEN1 | +1 | Speculations or questions rather than a conclusion (Cyclin-dependent kinase (Cdk) Cdk1-Cyclin A can phosphorylate Flap endonuclease 1 (Fen1)). | PMID:12853968 |
| VEGFA | BCL2 | +1 | Wrong target recognition and a general term appears in complex context (matrix metalloproteinase 9 (MMP9), vascular endothelial growth factor (VEGF), phosphorylated retinoblastoma protein (pRB), Bcl-2 and c-Abl). | PMID:16619307 |
| MAPK8 | HIF1A | +1 | Negation and negative words not recognized by text mining  (The activated p38MAPK, but not JNK1, phosphorylated HIF-1alpha). | PMID:16278378 |
| MAPK1 | FHL2 | +1 | Wrong direction recognition and Negation and negative words not recognized by text mining (The interaction between ERK2 and FHL2 did not influence ERK1/2 activation, nor was FHL2 directly phosphorylated by ERK2). | PMID:14729955 |
| DUSP5 | VEGFA | +1 | Wrong target recognition and a general term appears in complex context (whereas DUSP5 dephosphorylates VEGF – phosphorylated ERK1/2 inhibiting proliferation of endothelial cells). | PMID:19741200 |
| MAPK1 | FHL2 | +1 | Negation and negative words not recognized by text mining (The interaction between ERK2 and FHL2 did not influence ERK1/2 activation, nor was FHL2 directly phosphorylated by ERK2). | PMID:14729955 |
| CSNK2A2 | MAPK1 | +1 | No interaction found in STRING v9.0. |  |
| CSNK2A1 | MAPK1 | +1 | No interaction found in STRING v9.0. |  |
| CSNK2A2 | CDK5 | +1 | No interaction found in STRING v9.0. |  |
| CSNK2A1 | CDK5 | +1 | No interaction found in STRING v9.0. |  |

**Table S3: List of links between DNA damage and nodes in the PKT206 model.**

Column 1 shows the source node of the interaction; column 2 shows the target node of the interaction; column 3 shows the interaction type where -1 means inhibition and +1 means activation; column 4 shows the PubMed identifier of references used; column 5 shows the associated GO (Gene Ontology) term.

| Node A | Node B | Interaction type | PMID | GO |
| --- | --- | --- | --- | --- |
| DNA damage | TGFB1 | +1 | PMID:15652459 | GO:0001666: response to hypoxia |
| DNA damage | SOX4 | +1 | PMID:19234109 | GO:0042769: DNA damage response, detection of DNA damage |
| DNA damage | MDM2 | -1 | PMID:11960904  PMID:16227609 | GO:0006977: positive regulation of cell cycle arrest by p53-mediated DNA damage response. |
| DNA damage | ERBB2 | +1 | PMID:19406993 |  |
| DNA damage | NCL | +1 | PMID:12000845 |  |
| DNA damage | HTATIP2 | +1 | PMID:21376742 |  |
| DNA damage | PTTG1 | -1 | PMID:18047793 |  |
| DNA damage | BTG2 | +1 | PMID:8944033 | GO:0006974: response to DNA damage stimulus |
| DNA damage | MCTS1 | +1 | PMID:17016429 | GO:0006974: response to DNA damage stimulus |
| DNA damage | FAS | +1 | PMID:9660938 |  |
| DNA damage | YBX1 | +1 | PMID:12080043 | GO:0006355: regulation of transcription, DNA-dependent |
| DNA damage | MYC | +1 | PMID:2687769 | GO:0034644: cellular response to UV  GO:0006974: response to DNA damage stimulus |
| DNA damage | MYC | -1 | PMID:12761495 | GO:0034644: cellular response to UV  GO:0006974: response to DNA damage stimulus |
| DNA damage | CDK5 | +1 | PMID:19151707 |  |
| DNA damage | AURKA | -1 | PMID:21099343 |  |
| DNA damage | PRKD1 | +1 | PMID:16911582 |  |
| DNA damage | MAPK9 | +1 | PMID:17306896 |  |
| DNA damage | MAPK8 | +1 | PMID:15696159 | GO:0008624: induction of apoptosis by extracellular signals  GO:0008629: induction of apoptosis by intracellular signals  GO:0009411: response to UV |
| DNA damage | ATM | +1 | PMID:15279774  PMID:15322239 | GO:0008630: DNA damage response, signal transduction resulting in induction of apoptosis  GO: 0010212: response to ionizing radiation |
| DNA damage | ATR | +1 | PMID:15322239 | GO:0034644: cellular response to UV  GO:0006974: response to DNA damage stimulus |
| DNA damage | PRKDC | +1 | PMID:16908529 |  |

**Table S4: List of links between nodes in the PKT206 model and apoptosis.**

Column 1 shows the source node of the interaction; column 2 shows the target node of the interaction; column 3 shows the interaction type where -1 means anti-apoptotic and +1 means pro-apoptotic; column 4 shows the PubMed identifier of the references used; column 5 shows the GO (Gene Ontology) term.

| Node A | Node B | Interaction type | PMID | GO |
| --- | --- | --- | --- | --- |
| BCL3 | apoptosis | -1 | PMID:20800578 | GO:0043066: negative regulation of apoptotic process |
| GSTP1 | apoptosis | -1 | PMID:21637416 |  |
| ESR1 | apoptosis | -1 | PMID:9118519 | GO:0042981: regulation of apoptotic process |
| ESR1 | apoptosis | +1 | PMID:17615152 | GO:0042981: regulation of apoptotic process |
| ECT2 | apoptosis | +1 | PMID:12787561 |  |
| MSH2 | apoptosis | +1 | PMID:10097137 | GO:0043524: negative regulation of neuron apoptosis |
| DUSP4 | apoptosis | +1 | PMID:20124482 |  |
| DUSP4 | apoptosis | -1 | PMID:20860659 |  |
| CXCR4 | apoptosis | +1 | PMID:15705741 | GO:0006915: apoptotic process |
| XAF1 | apoptosis | +1 | PMID:21788101 | GO:0006915: apoptotic process |
| IL6 | apoptosis | -1 | PMID:11751424 | GO:0043154: negative regulation of cysteine-type endopeptidase activity involved in apoptotic process  GO:0001781: neutrophil apoptosis  GO:0042981: regulation of apoptotic process |
| IL6 | apoptosis | +1 | PMID:12714376 | GO:0043154: negative regulation of cysteine-type endopeptidase activity involved in apoptotic process  GO:0001781: neutrophil apoptosis  GO:0042981: regulation of apoptotic process |
| PDGFRB | apoptosis | -1 | PMID:21954875 |  |
| FGF2 | apoptosis | -1 | PMID:15856005 | GO:0006915: apoptotic process |
| FGF2 | apoptosis | +1 | PMID:20081577 | GO:0006915: apoptotic process |
| WWP1 | apoptosis | -1 | PMID:18806757 |  |
| IGF1R | apoptosis | -1 | PMID:14726697 | GO:0006916: anti-apoptosis  GO:0043066: negative regulation of apoptotic process  GO:0045768: positive regulation of anti-apoptosis |
| EGFR | apoptosis | +1 | PMID:11226409 |  |
| EGFR | apoptosis | -1 | PMID:15277479 | GO:0043066: negative regulation of apoptotic process |
| CD44 | apoptosis | +1 | PMID:16208414 |  |
| CD44 | apoptosis | -1 | PMID:17045821 |  |
| PRKCA | apoptosis | +1 | PMID:10825394 | GO:0006915: apoptotic process |
| PRKCA | apoptosis | -1 | DOI:10.1007/978-1-59745-199-4_2 | GO:0006915: apoptotic process  GO:0034351: negative regulation of glial cell apoptosis |
| DUSP2 | apoptosis | +1 | PMID:12673251  PMID:9501207 | GO:0042981: regulation of apoptotic process |
| DUSP2 | apoptosis | -1 | PMID:12673251  PMID:9501207 | GO:0042981: regulation of apoptotic process |
| FDXR | apoptosis | +1 | PMID:12370809 |  |
| BAX | apoptosis | +1 | PMID:19672311 | GO:0042981: regulation of apoptotic process |
| TGFA | apoptosis | -1 | PMID:16079309 | GO:0006916: anti-apoptosis  GO:0043524: negative regulation of neuron apoptosis |
| IFI16 | apoptosis | +1 | PMID:14990579 | GO:0042771: DNA damage response, signal transduction by p53 class mediator resulting in induction of apoptosis |
| TLR3 | apoptosis | +1 | PMID:16585585 | GO:0043065: positive regulation of apoptotic process |
| CDC25A | apoptosis | +1 | PMID:15822194 ;  PMID:20368335 |  |
| CDC25A | apoptosis | -1 | PMID:11416155 |  |
| FOS | apoptosis | -1 | PMID:19255142 |  |
| FOS | apoptosis | +1 | PMID:8524298 |  |
| DDIT4 | apoptosis | -1 | PMID:19221489 | GO:0006915: apoptotic process |
| DDIT4 | apoptosis | +1 | PMID:18796435 | GO:0006915: apoptotic process |
| MAP4K4 | apoptosis | -1 | PMID:21196414 |  |
| MAP4K4 | apoptosis | +1 | PMID:15958553 |  |
| SEMA3B | apoptosis | +1 | PMID:15273288 |  |
| EZH2 | apoptosis | -1 | PMID:19893569;  PMID:19079346 |  |
| PSEN1 | apoptosis | -1 | PMID:10805794 | GO:0006916: anti-apoptosis  GO:0006915: apoptotic process  GO:0043524: negative regulation of neuron apoptosis |
| PEG3 | apoptosis | +1 | PMID:11050235;  PMID:10681424 | GO:0006915: apoptotic process |
| PRSS50 | apoptosis | +1 | PMID:21086474 |  |
| IFITM2 | apoptosis | +1 | PMID:19544527 |  |
| SIVA1 | apoptosis | +1 | PMID:20727854 | GO:0006915: apoptotic process  GO:0006917: induction of apoptosis |
| BCL2 | apoptosis | -1 | PMID:8617294 | GO:0006916: anti-apoptosis  GO:0043066: negative regulation of apoptotic process  GO:0042981: regulation of apoptotic process |
| PCBP4 | apoptosis | +1 | PMID : 20817677 | GO:0008630: DNA damage response, signal transduction resulting in induction of apoptosis |
| DFNA5 | apoptosis | +1 | PMID:21522185 |  |
| HNF4A | apoptosis | +1 | PMID:19835622 |  |
| TP53INP1 | apoptosis | +1 | PMID:21219856 | GO:0006917: induction of apoptosis |
| FHL2 | apoptosis | -1 | PMID:21377781 |  |
| CCNG1 | apoptosis | -1 | PMID:18497347 | GO:0043066: negative regulation of apoptotic process |
| CCNG1 | apoptosis | +1 | PMID:10467405 |  |
| TCF7L2 | apoptosis | -1 | PMID:21357677  PMID:21965303 |  |
| EPHB4 | apoptosis | -1 | PMID:20133814 |  |
| CASP8 | apoptosis | +1 | PMID:15029256 | GO:0042981: regulation of apoptotic process |
| CDKN1B | apoptosis | -1 | PMID:10050878 |  |
| CDKN1B | apoptosis | +1 | PMID:10208428 |  |
| COL18A1 | apoptosis | +1 | PMID:11158588 |  |
| BBC3 | apoptosis | +1 | PMID:11572983 | GO:0008633: activation of pro-apoptotic gene products  GO:0006915: apoptotic process  GO:0006917: induction of apoptosis  GO:0070245: positive regulation of thymocyte apoptosis |
| APAF1 | apoptosis | +1 | PMID:10791976 | GO:0006915: apoptotic process  GO:0042981: regulation of apoptotic process |
| BAK1 | apoptosis | +1 | PMID:20460378 | GO:0042981: regulation of apoptotic process |
| NLRC4 | apoptosis | +1 | PMID:11374873 | GO:0043065: positive regulation of apoptotic process |
| ATF3 | apoptosis | -1 | PMID:12392999 |  |
| ATF3 | apoptosis | +1 | PMID:18755691 |  |
| PTGS2 | apoptosis | +1 | PMID:16544098 |  |
| PTGS2 | apoptosis | -1 | PMID:11046152 |  |
| SGK | apoptosis | -1 | PMID:12488318 |  |
| MCL1 | apoptosis | -1 | PMID:18550749 | GO:0042981: regulation of apoptotic process |
| IER3 | apoptosis | -1 | PMID:14688131 | GO:0006916: anti-apoptosis |
| GADD45A | apoptosis | +1 | PMID:17474084 | GO:0006915: apoptotic process |
| NOTCH1 | apoptosis | -1 | PMID:10227380 | GO:0060548: negative regulation of cell death |
| NOTCH1 | apoptosis | +1 | PMID:12815466 | GO:0043065: positive regulation of apoptotic process |
| FAS | apoptosis | +1 | PMID:7536620 | GO:0006915: apoptotic process |
| PTEN | apoptosis | +1 | PMID:11159942 | GO:0006917: induction of apoptosis |
| VEGFA | apoptosis | -1 | PMID:11891765 | GO:0006916: anti-apoptosis  GO:0043066: negative regulation of apoptotic process |
| AIFM2 | apoptosis | +1 | PMID:21943319 | GO:0006917: induction of apoptosis |
| CDKN1A | apoptosis | -1 | PMID:21815189 | GO:0008629: induction of apoptosis by intracellular signals  GO:0043066: negative regulation of apoptotic process |
| DKK1 | apoptosis | +1 | PMID:17026960; PMID:20549706 |  |
| AR | apoptosis | -1 | PMID:16479009 | GO:0043066: negative regulation of apoptotic process |
| AR | apoptosis | +1 | PMID:16479009 |  |
| CKS2 | apoptosis | -1 | PMID:18498131 |  |
| BNIP3L | apoptosis | +1 | PMID:10381623 | GO:0043065: positive regulation of apoptotic process |
| TNFRSF10A | apoptosis | +1 | PMID:21785270 | GO:0006917: induction of apoptosis  GO:0008625: induction of apoptosis via death domain receptors |
| TNFRSF10B | apoptosis | +1 | PMID:22046379 | GO:0008633: activation of pro-apoptotic gene products  GO:0006915: apoptotic process  GO:0008625: induction of apoptosis via death domain receptors  GO:0042981: regulation of apoptotic process |
| IGFBP7 | apoptosis | +1 | PMID:18267069 |  |
| LATS2 | apoptosis | +1 | PMID:15265683 |  |
| SERPINB5 | apoptosis | +1 | PMID:15713631 |  |
| C12orf5 | apoptosis | -1 | PMID:16839880 |  |
| PERP | apoptosis | +1 | PMID:10733530 | GO:0006915: apoptotic process |
| SIAH1 | apoptosis | +1 | PMID:9403064 | GO:0006915: apoptotic process  GO:0051402: neuron apoptosis  GO:00043065: positive regulation of apoptotic process |
| LRDD | apoptosis | +1 | PMID:16183742 | GO:0043066: negative regulation of apoptotic process |
| P53AIP1 | apoptosis | +1 | PMID:16467208 | GO:0006915: apoptotic process |
| SFN | apoptosis | -1 | PMID:15857577 ;  PMID:10654934 | GO:0006915: apoptotic process  GO:0008630: DNA damage response, signal transduction resulting in induction of apoptosis |
| CDKN2A | apoptosis | +1 | PMID:12660818 | GO:0006917: induction of apoptosis |

**Table S5: List of links between nodes in the PKT206 model and cellular senescence.**

Column 1 shows the source node of the interaction; column 2 shows the target node of the interaction; column 3 shows the interaction type where -1 means repressor of senescence and +1 means inducer of senescence; column 4 shows the PubMed identifier of the references used; column 5 shows the GO (Gene Ontology) term.

| Node A | Node B | Interaction type | PMID | GO |
| --- | --- | --- | --- | --- |
| GAPDH | Cellular senescence | -1 | PMID:21749859 |  |
| GAPDH | Cellular senescence | +1 | PMID:22847419 |  |
| MSH2 | Cellular senescence | -1 | PMID:23213348 |  |
| MSH2 | Cellular senescence | +1 | PMID:18986375 |  |
| RRM2B | Cellular senescence | -1 | PMID:23139867 |  |
| DDB2 | Cellular senescence | +1 | PMID:23109835 |  |
| CDK4 | Cellular senescence | -1 | PMID:12435633 |  |
| IL6 | Cellular senescence | +1 | PMID:22374671 |  |
| ARID3A | Cellular senescence | -1 | PMID:22010578 |  |
| FGF2 | Cellular senescence | -1 | PMID:17532297  PMID:21990129 |  |
| WWP1 | Cellular senescence | -1 | PMID:22051607  PMID:21795702 |  |
| IGF1R | Cellular senescence | +1 | PMID:18216278 |  |
| EGFR | Cellular senescence | -1 | PMID:21852385 |  |
| CDKN1A | Cellular senescence | +1 | PMID:15149599 | GO:0090398: cellular senescence |
| PRKCA | Cellular senescence | +1 | PMID:18162471 |  |
| TGFA | Cellular senescence | +1 | PMID:12593448 |  |
| IFI16 | Cellular senescence | +1 | PMID:15208661 |  |
| CKB | Cellular senescence | -1 | PMID:21980054 |  |
| DDIT4 | Cellular senescence | -1 | PMID:22629318 |  |
| EZH2 | Cellular senescence | -1 | PMID:15208672  PMID:21383005 |  |
| PSEN1 | Cellular senescence | +1 | PMID:19181896 |  |
| IFITM2 | Cellular senescence | +1 | PMID:19071156 |  |
| BCL2 | Cellular senescence | -1 | PMID:19855432 |  |
| BCL2 | Cellular senescence | +1 | PMID:12670482 |  |
| PCBP4 | Cellular senescence | -1 | PMID:20817677 |  |
| HIF1A | Cellular senescence | -1 | PMID:18645006 |  |
| CD59 | Cellular senescence | -1 | PMID:17188915 |  |
| CD59 | Cellular senescence | +1 | PMID:22918646 |  |
| FOXM1 | Cellular senescence | -1 | PMID:23262037 |  |
| HNF4A | Cellular senescence | -1 | PMID:21385945 |  |
| FHL2 | Cellular senescence | -1 | PMID:19018287 |  |
| BRCA1 | Cellular senescence | -1 | PMID:12533509 |  |
| CDKN1B | Cellular senescence | +1 | PMID:21795702 |  |
| APAF1 | Cellular senescence | -1 | PMID:17652622 |  |
| BAK1 | Cellular senescence | +1 | PMID:19747230 |  |
| BAK1 | Cellular senescence | -1 | PMID:11557285 |  |
| PTGS2 | Cellular senescence | +1 | PMID:23328527 |  |
| SGK | Cellular senescence | -1 | PMID:15068796 |  |
| S100A6 | Cellular senescence | -1 | PMID:23095053 |  |
| MCL1 | Cellular senescence | -1 | PMID:22451485 |  |
| GADD45A | Cellular senescence | +1 | PMID:16951155 |  |
| GADD45A | Cellular senescence | -1 | PMID:21986581 |  |
| NOTCH1 | Cellular senescence | +1 | PMID:23078884 |  |
| PTEN | Cellular senescence | -1 | PMID:21072054 |  |
| PTEN | Cellular senescence | +1 | PMID:23314408 |  |
| VEGFA | Cellular senescence | -1 | PMID:21618508 |  |
| DKK1 | Cellular senescence | +1 | PMID:22927647 |  |
| DKK1 | Cellular senescence | -1 | PMID:21712954 |  |
| AR | Cellular senescence | +1 | PMID:22403609 |  |
| TFDP1 | Cellular senescence | -1 | PMID:15716376 |  |
| NME1 | Cellular senescence | -1 | PMID:20713695 |  |
| MYC | Cellular senescence | +1 | PMID:20027199 |  |
| MYC | Cellular senescence | -1 | PMID:17664422 |  |
| IGFBP7 | Cellular senescence | +1 | PMID:21997538 |  |
| LATS2 | Cellular senescence | +1 | PMID:21498571 |  |
| RAS | Cellular senescence | +1 | PMID:15489886 | GO:0090398: cellular senescence |
| C12orf5 | Cellular senescence | +1 | PMID:19710698 |  |
| SESN2 | Cellular senescence | -1 | PMID:20606249 |  |
| SFN | Cellular senescence | +1 | PMID:19642975 |  |
| CDKN2A | Cellular senescence | +1 | PMID:14966292 | GO:0090398: cellular senescence  GO:2000774: positive regulation of cellular senescence |
| PPM1D | Cellular senescence | -1 | PMID:22201816 |  |

**Table S6: Alterations in dependency matrix upon gene deletions**

A single gene was removed from the PKT206 model for each knock-out test shown below. The numbers of six types of effect elements in the dependency matrix were calculated and listed. The value “Null” in the selected gene column indicates p53 wild type.

| Selected gene | Total number of elements | No effect | Ambivalent factor | Weak inhibitor | Weak activator | Strong inhibitor | Strong activator |
| --- | --- | --- | --- | --- | --- | --- | --- |
| Null | 42436 | 23468 | 16540 | 1100 | 1240 | 33 | 55 |
| P53 | 42025 | 35009 | 6772 | 44 | 84 | 43 | 73 |
| MYC | 42025 | 23505 | 16009 | 1133 | 1289 | 34 | 55 |
| VEGFA | 42025 | 24214 | 15269 | 1137 | 1307 | 36 | 62 |
| PTGS2 | 42025 | 23506 | 15919 | 1161 | 1151 | 33 | 55 |
| CCND1 | 42025 | 23673 | 15666 | 1216 | 1381 | 34 | 55 |
| TGFB1 | 42025 | 23780 | 15658 | 1181 | 1316 | 33 | 57 |
| IL6 | 42025 | 23337 | 16232 | 1111 | 1257 | 33 | 55 |
| MDM2 | 42025 | 23445 | 16039 | 1166 | 1285 | 33 | 57 |
| E2F1 | 42025 | 23765 | 15637 | 1187 | 1336 | 33 | 67 |
| IFNA1 | 42025 | 23228 | 16370 | 1100 | 1240 | 33 | 54 |
| EGFR | 42025 | 23673 | 15925 | 1099 | 1239 | 34 | 55 |
| FOS | 42025 | 23506 | 16092 | 1100 | 1239 | 33 | 55 |
| BCL2 | 42025 | 23337 | 16173 | 1155 | 1272 | 33 | 55 |
| CDKN1B | 42025 | 23337 | 16259 | 1101 | 1240 | 33 | 55 |
| RAS | 42025 | 23337 | 16233 | 1111 | 1256 | 33 | 55 |
| FGF2 | 42025 | 23337 | 16287 | 1084 | 1229 | 33 | 55 |
| MMP2 | 42025 | 23173 | 16429 | 1100 | 1240 | 33 | 50 |
| PRKCA | 42025 | 23337 | 16256 | 1100 | 1244 | 33 | 55 |
| MAPK8 | 42025 | 23337 | 16209 | 1127 | 1264 | 33 | 55 |
| HIF1A | 42025 | 23779 | 15821 | 1100 | 1234 | 33 | 58 |
| ESR1 | 42025 | 23447 | 16124 | 1116 | 1250 | 33 | 55 |
| CXCR4 | 42025 | 23505 | 16069 | 1108 | 1254 | 33 | 56 |
| CDK2 | 42025 | 23504 | 16090 | 1099 | 1242 | 34 | 56 |
| ABCB1 | 42025 | 23168 | 16429 | 1100 | 1240 | 33 | 55 |
| MMP1 | 42025 | 23170 | 16429 | 1100 | 1240 | 32 | 54 |
| BRCA1 | 42025 | 23337 | 16259 | 1100 | 1241 | 33 | 55 |
| PTEN | 42025 | 23337 | 16233 | 1116 | 1251 | 33 | 55 |
| CCNA | 42025 | 23337 | 16231 | 1112 | 1257 | 33 | 55 |
| CSNK2 | 42025 | 23229 | 16370 | 1100 | 1240 | 33 | 53 |
| ATM | 42025 | 23231 | 16370 | 1100 | 1240 | 31 | 53 |
| NOTCH1 | 42025 | 23337 | 16260 | 1100 | 1240 | 33 | 55 |

**Table S7: Major changes observed in *in silico* gene deletions.**

This table lists all elements from the dependency matrix of the p53 wild-type that were changed to strong inhibitor or strong activator in the dependency matrix of the selected gene deletion.

| Serial No | Name of gene deleted | Protein A | Protein B | Effect in p53 wild-type | Effect in knock-out |
| --- | --- | --- | --- | --- | --- |
| 1 | P53 | SGK | Apoptosis | Ambivalent Factor | Strong inhibitor |
| 2 | P53 | KLF4 | CCNB1 | Ambivalent factor | Strong inhibitor |
| 3 | P53 | IFNA1 | CDK4 | Ambivalent factor | Strong inhibitor |
| 4 | P53 | IFNA1 | FGF2 | Ambivalent factor | Strong inhibitor |
| 5 | P53 | PPM1D | CHEK1 | Weak inhibitor | Strong inhibitor |
| 6 | P53 | SFN | CCNB1 | Ambivalent factor | Strong inhibitor |
| 7 | P53 | DNA damage | CDK4 | Ambivalent factor | Strong inhibitor |
| 8 | P53 | DNA damage | FGF2 | Ambivalent factor | Strong inhibitor |
| 9 | P53 | FGF2 | CDK4 | Ambivalent factor | Strong activator |
| 10 | P53 | FOXM1 | CCNB1 | Ambivalent factor | Strong activator |
| 11 | P53 | FAS | Apoptosis | Ambivalent factor | Strong activator |
| 12 | P53 | LATS2 | Apoptosis | Ambivalent factor | Strong activator |
| 13 | P53 | PTTG1 | CDK4 | Ambivalent factor | Strong activator |
| 14 | P53 | PTTG1 | FGF2 | Ambivalent factor | Strong activator |
| 15 | P53 | IFNA1 | TLR3 | Ambivalent factor | Strong activator |
| 16 | P53 | IFNA1 | FAS | Ambivalent factor | Strong activator |
| 17 | P53 | DYRK2 | P53AIP1 | Weak activator | Strong activator |
| 18 | P53 | DYRK2 | Apoptosis | Ambivalent factor | Strong activator |
| 19 | P53 | ATM | CHEK1 | Ambivalent factor | Strong activator |
| 20 | P53 | ATR | CHEK1 | Ambivalent factor | Strong activator |
| 21 | P53 | MAPK14 | MMP2 | Ambivalent factor | Strong activator |
| 22 | P53 | MAPK14 | BAX | Ambivalent factor | Strong activator |
| 23 | P53 | MAPK14 | SGK | Ambivalent factor | Strong activator |
| 24 | P53 | DNA damage | CHEK1 | Ambivalent factor | Strong activator |
| 25 | P53 | DNA damage | FAS | Ambivalent factor | Strong activator |
| 26 | MYC | TCF7L2 | Apoptosis | Ambivalent factor | Strong inhibitor |
| 27 | VEGFA | TLR3 | CXCR4 | Weak inhibitor | Strong inhibitor |
| 28 | VEGFA | TLR3 | TNFRSF10B | Ambivalent factor | Strong inhibitor |
| 29 | VEGFA | CXCR4 | TNFRSF10B | Ambivalent factor | Strong activator |
| 30 | VEGFA | CXCR4 | Apoptosis | Ambivalent factor | Strong activator |
| 31 | VEGFA | FOXM1 | MMP2 | Ambivalent factor | Strong activator |
| 32 | VEGFA | FOXM1 | BAX | Ambivalent factor | Strong activator |
| 33 | VEGFA | FOXM1 | CCNB1 | Ambivalent factor | Strong activator |
| 34 | VEGFA | FOXM1 | Apoptosis | Ambivalent factor | Strong activator |
| 35 | VEGFA | SERPINB5 | Apoptosis | Ambivalent factor | Strong activator |
| 36 | CCND1 | PDGFRB | Apoptosis | Ambivalent factor | Strong inhibitor |
| 37 | TGFB1 | DKK1 | Apoptosis | Ambivalent factor | Strong activator |
| 38 | TGFB1 | DNA damage | MAPK8 | Ambivalent factor | Strong activator |
| 39 | MDM2 | ATM | DYRK2 | Ambivalent factor | Strong activator |
| 40 | MDM2 | DNA damage | DYRK2 | Ambivalent factor | Strong activator |
| 41 | E2F1 | AATF | CDK5 | Weak activator | Strong activator |
| 42 | E2F1 | CHEK2 | AATF | Weak activator | Strong activator |
| 43 | E2F1 | CHEK2 | CDK5 | Weak activator | Strong activator |
| 44 | E2F1 | CSNK2 | MYCN | Ambivalent factor | Strong activator |
| 45 | E2F1 | ATM | AATF | Ambivalent factor | Strong activator |
| 46 | E2F1 | ATM | CHEK2 | Ambivalent factor | Strong activator |
| 47 | E2F1 | ATM | CDK5 | Ambivalent factor | Strong activator |
| 48 | E2F1 | ATR | AATF | Ambivalent factor | Strong activator |
| 49 | E2F1 | ATR | CDK5 | Ambivalent factor | Strong activator |
| 50 | E2F1 | DNA damage | AATF | Ambivalent factor | Strong activator |
| 51 | E2F1 | DNA damage | CHEK2 | Ambivalent factor | Strong activator |
| 52 | E2F1 | DNA damage | CDK5 | Ambivalent factor | Strong activator |
| 53 | EGFR | BCL3 | Apoptosis | Ambivalent factor | Strong inhibitor |
| 54 | HIF1A | GAPDH | SIAH1 | Weak activator | Strong activator |
| 55 | HIF1A | GAPDH | Apoptosis | Ambivalent factor | Strong activator |
| 56 | HIF1A | SIAH1 | Apoptosis | Ambivalent factor | Strong activator |
| 57 | CXCR4 | TLR3 | Apoptosis | Ambivalent factor | Strong activator |
| 58 | CDK2 | CDKN1A | Apoptosis | Ambivalent factor | Strong inhibitor |
| 59 | P53 | SGK | Cellular senescence | Ambivalent factor | Strong inhibitor |
| 60 | P53 | MAPK14 | Cellular senescence | Ambivalent factor | Strong inhibitor |
| 61 | P53 | LATS2 | Cellular senescence | Ambivalent factor | Strong activator |
| 62 | VEGFA | FOXM1 | Cellular senescence | Ambivalent factor | Strong inhibitor |
| 63 | CDK2 | CDKN1A | Cellular senescence | Ambivalent factor | Strong activator |

**Table S8: Logical steady state analysis results of the PKT206 model.**

This table lists the results of logical steady state analysis for four scenarios. In each scenario, a value of 1 means that the node is active (ON), a value of 0 means that it is inactivate (OFF) and NaN means that its state is undetermined. Undetermined nodes can take either value creating multiple possible steady states in each scenario.

| Node name | Scenario 1: p53 wild-type when DNA damage is ON | Scenario 2: p53 wild-type when DNA damage is OFF | Scenario 3: p53 mutant when DNA damage is ON | Scenario 4: p53 mutant when DNA damage is OFF |
| --- | --- | --- | --- | --- |
| HOXA11 | NaN | NaN | NaN | NaN |
| P53 | 1 | 1 |  |  |
| NTN1 | NaN | NaN | NaN | NaN |
| PSMD10 | NaN | NaN | NaN | NaN |
| TGFB1 | 1 | 1 | 1 | 1 |
| AATF | 1 | 1 | 1 | 1 |
| LTF | 1 | 1 | 1 | 1 |
| BCL6 | 0 | 1 | 0 | 1 |
| SOX4 | 1 | 0 | 1 | 0 |
| SERPINF1 | 1 | 1 | 1 | 1 |
| MDM2 | 1 | 1 | NaN | 1 |
| DDX5 | NaN | NaN | NaN | NaN |
| ELAVL1 | NaN | NaN | NaN | NaN |
| IGF1R | 0 | 0 | NaN | NaN |
| ABCC1 | 1 | 1 | 1 | 1 |
| BCL3 | 0 | 0 | NaN | NaN |
| GSTP1 | 1 | 1 | 1 | 1 |
| PDRG1 | 0 | 0 | NaN | NaN |
| ESR1 | 1 | 1 | 1 | 1 |
| BDKRB1 | 0 | 0 | NaN | NaN |
| MMP2 | 1 | 1 | 1 | 1 |
| CKM | 1 | 1 | 0 | 0 |
| CD82 | 1 | 1 | NaN | NaN |
| CCND1 | 1 | 1 | 1 | 1 |
| GAPDH | 1 | 1 | NaN | NaN |
| ECT2 | 0 | 0 | NaN | NaN |
| MSH2 | 1 | 1 | 1 | 1 |
| CD58 | 1 | 1 | NaN | NaN |
| DUSP4 | 1 | 1 | NaN | NaN |
| CXCR4 | 1 | 1 | 1 | 1 |
| RRM2B | 1 | 1 | NaN | NaN |
| EDA2R | 1 | 1 | NaN | NaN |
| SLC6A6 | 0 | 0 | NaN | NaN |
| XAF1 | 0 | 0 | NaN | NaN |
| DDB2 | 1 | 1 | NaN | NaN |
| CDK4 | NaN | 1 | NaN | 1 |
| IL6 | 1 | 1 | 1 | 1 |
| NOV | 1 | 1 | NaN | NaN |
| THBS1 | 1 | 1 | 1 | 1 |
| PDGFRB | 0 | 0 | NaN | NaN |
| ARID3A | 1 | 1 | NaN | NaN |
| KAT2B | 1 | 1 | NaN | NaN |
| FGF2 | NaN | 1 | NaN | 1 |
| ICAM1 | 1 | 1 | 1 | 1 |
| WWP1 | 0 | 0 | NaN | NaN |
| ABCB1 | 1 | 1 | 1 | 1 |
| NR2C1 | 1 | 1 | 1 | 1 |
| EGFR | 1 | 1 | 1 | 1 |
| IGFBP1 | 1 | 1 | NaN | NaN |
| CD44 | 1 | 1 | 1 | 1 |
| CHEK1 | 1 | 0 | 1 | NaN |
| PRKCA | 1 | 1 | 1 | 1 |
| DUSP2 | 1 | 1 | NaN | NaN |
| FDXR | 1 | 1 | NaN | NaN |
| BAX | 1 | 1 | 1 | 1 |
| KRT8 | 1 | 1 | NaN | NaN |
| TGFA | 1 | 1 | 1 | 1 |
| IFI16 | 1 | 1 | NaN | NaN |
| TLR3 | 1 | 1 | NaN | NaN |
| CKB | 1 | 1 | 1 | 1 |
| TP53I13 | 1 | 1 | NaN | NaN |
| RECQL4 | 0 | 0 | NaN | NaN |
| MGMT | 0 | 0 | NaN | NaN |
| SP7 | 0 | 0 | NaN | NaN |
| HSPA4 | 1 | 1 | 1 | 1 |
| CDC25A | 1 | 1 | 1 | 1 |
| FEN1 | 1 | 1 | 0 | 0 |
| FOS | 1 | 1 | 1 | 1 |
| DDIT4 | 1 | 1 | NaN | NaN |
| CDC20 | 0 | 0 | NaN | NaN |
| IQCB1 | 0 | 0 | NaN | NaN |
| MAP4K4 | 1 | 1 | NaN | NaN |
| HIC1 | 1 | 1 | 1 | 1 |
| SEMA3B | 1 | 1 | NaN | NaN |
| RPRM | 1 | 1 | NaN | NaN |
| EZH2 | 1 | 1 | 1 | 1 |
| MMP1 | 1 | 1 | 1 | 1 |
| PSEN1 | 1 | 1 | 1 | 1 |
| PEG3 | 1 | 1 | NaN | NaN |
| PRSS50 | 0 | 0 | NaN | NaN |
| IFITM2 | 0 | 0 | NaN | NaN |
| SIVA1 | 1 | 1 | 1 | 1 |
| HSP90AB1 | 0 | 0 | NaN | NaN |
| BCL2 | 1 | 1 | 1 | 1 |
| PCBP4 | 1 | 1 | NaN | NaN |
| HIF1A | 1 | 1 | 1 | 1 |
| DFNA5 | 1 | 1 | NaN | NaN |
| MMP13 | 1 | 1 | 1 | 1 |
| CD59 | 1 | 1 | NaN | NaN |
| FOXM1 | 0 | 0 | NaN | NaN |
| HNF4A | 0 | 0 | NaN | NaN |
| TP53INP1 | 1 | 1 | NaN | NaN |
| FHL2 | 1 | 1 | NaN | NaN |
| CCNG1 | 1 | 1 | 1 | 1 |
| TCF7L2 | NaN | NaN | NaN | NaN |
| BRCA1 | 1 | 1 | 1 | 1 |
| EPHB4 | 0 | 0 | NaN | NaN |
| CASP8 | 1 | 1 | NaN | NaN |
| HMMR | 0 | 0 | NaN | NaN |
| CDKN1B | 1 | 1 | 1 | 1 |
| COL18A1 | 1 | 1 | NaN | NaN |
| BBC3 | 1 | 1 | NaN | NaN |
| APAF1 | 1 | 1 | 1 | 1 |
| MAP4 | 0 | 0 | NaN | NaN |
| BAK1 | 1 | 1 | NaN | NaN |
| NLRC4 | 1 | 1 | NaN | NaN |
| PRC1 | 0 | 0 | NaN | NaN |
| GTSE1 | 1 | 1 | NaN | NaN |
| CALD1 | 1 | 1 | NaN | NaN |
| KRT19 | 0 | 0 | 0 | 0 |
| ATF3 | 1 | 1 | 1 | 1 |
| PTGS2 | 1 | 1 | 1 | 1 |
| RGS16 | 1 | 1 | NaN | NaN |
| SGK | 1 | 1 | 1 | 1 |
| S100A2 | 1 | 1 | NaN | NaN |
| S100A6 | 0 | 0 | NaN | NaN |
| MCL1 | 1 | 1 | 1 | 1 |
| DUSP5 | 1 | 1 | 1 | 1 |
| IER3 | 1 | 1 | NaN | NaN |
| GADD45A | 1 | 1 | 0 | 0 |
| NOTCH1 | 1 | 1 | 1 | 1 |
| FAS | 1 | 1 | 1 | NaN |
| PTEN | 1 | 1 | NaN | NaN |
| VEGFA | 1 | 1 | 1 | 1 |
| SLC2A1 | 1 | 1 | 1 | 1 |
| AIFM2 | 1 | 1 | NaN | NaN |
| CDKN1A | 1 | 1 | 1 | 1 |
| DKK1 | 1 | 1 | NaN | NaN |
| PRKG1 | 1 | 1 | NaN | NaN |
| AR | 0 | 0 | 0 | 0 |
| TFDP1 | 0 | 0 | NaN | NaN |
| CKS2 | 0 | 0 | NaN | NaN |
| NME1 | 1 | 1 | 1 | 1 |
| MYC | 1 | 1 | 1 | 1 |
| PCNA | 1 | 1 | 1 | 1 |
| C13orf15 | 1 | 1 | NaN | NaN |
| ISG15 | 1 | 1 | NaN | NaN |
| BNIP3L | 1 | 1 | NaN | NaN |
| SLC2A4 | 0 | 0 | NaN | NaN |
| CCNB1 | 0 | 0 | NaN | NaN |
| TNFRSF10A | 1 | 1 | 1 | 1 |
| TNFRSF10B | 1 | 1 | 1 | 1 |
| IGFBP7 | 1 | 1 | NaN | NaN |
| LATS2 | 1 | 1 | NaN | NaN |
| RAD51 | 0 | 0 | 0 | 0 |
| SERPINB5 | 1 | 1 | NaN | NaN |
| RAS | 1 | 1 | 1 | 1 |
| ERBB2 | 1 | 1 | 1 | 1 |
| MYCN | 1 | 1 | 1 | 1 |
| ZMAT3 | 1 | 1 | NaN | NaN |
| NCL | 1 | NaN | 1 | NaN |
| KLF4 | 1 | 1 | NaN | NaN |
| PPM1A | NaN | NaN | NaN | NaN |
| PLAUR | 1 | 1 | 1 | 1 |
| HTATIP2 | 1 | 0 | 1 | 0 |
| PTTG1 | 0 | 1 | 0 | 1 |
| E2F1 | 1 | 1 | 1 | 1 |
| TIAF1 | NaN | NaN | NaN | NaN |
| CIAPIN1 | NaN | NaN | NaN | NaN |
| PARK2 | NaN | NaN | NaN | NaN |
| BTG2 | 1 | 0 | 1 | 0 |
| MUC1 | NaN | NaN | NaN | NaN |
| MCTS1 | 1 | 0 | 1 | 0 |
| YBX1 | 1 | 1 | 1 | 1 |
| HDAC1 | NaN | NaN | NaN | NaN |
| ID3 | 1 | 1 | 1 | 1 |
| PADI4 | NaN | NaN | NaN | NaN |
| POU4F1 | NaN | NaN | NaN | NaN |
| RREB1 | NaN | NaN | NaN | NaN |
| IFNA1 | NaN | NaN | NaN | NaN |
| CHEK2 | 1 | 1 | 1 | 1 |
| AXIN1 | 1 | 1 | 1 | 1 |
| CCNA | 1 | 1 | 1 | 1 |
| H2AFZ | NaN | NaN | NaN | NaN |
| MAPK8 | 1 | 1 | 1 | 1 |
| CDK5 | 1 | 1 | 1 | 1 |
| HIPK2 | NaN | NaN | NaN | NaN |
| CDKN2A | 0 | 0 | NaN | NaN |
| MAPK1 | 1 | 1 | 1 | 1 |
| CSNK2 | NaN | NaN | NaN | NaN |
| PRKD1 | 1 | 0 | 1 | 0 |
| MAPK9 | 1 | 0 | 1 | 0 |
| CDK2 | 1 | 1 | 1 | 1 |
| EIF2AK2 | NaN | NaN | NaN | NaN |
| VRK1 | NaN | NaN | NaN | NaN |
| RAF1 | 1 | 1 | 1 | 1 |
| HIPK4 | NaN | NaN | NaN | NaN |
| PPM1D | 1 | 1 | NaN | NaN |
| AURKA | 0 | 1 | 0 | 1 |
| DYRK2 | 1 | 0 | 1 | 0 |
| CDK9 | NaN | NaN | NaN | NaN |
| ATM | 1 | 0 | 1 | 0 |
| ATR | 1 | 0 | 1 | 0 |
| PRKDC | 1 | 0 | 1 | 0 |
| MDM4 | 1 | 1 | NaN | 1 |
| C12orf5 | 1 | 1 | NaN | NaN |
| PERP | 1 | 1 | NaN | NaN |
| SIAH1 | 1 | 1 | 1 | 1 |
| LRDD | 1 | 1 | NaN | NaN |
| p53AIP1 | 1 | 1 | 1 | 0 |
| SESN2 | 1 | 1 | NaN | NaN |
| SFN | 1 | 1 | NaN | NaN |
| MAPK14 | 1 | 1 | 1 | 1 |
| DNAdamage | 1 | 0 | 1 | 0 |
| Apoptosis | 1 | 1 | 1 | 1 |
| Senescence | 1 | 1 | 1 | 1 |

**Table S9: Number of anti-apoptotic genes and genes that prevent cellular senescence with altered expression depending on p53 and DNA damage.**

(a) The numbers of anti-apoptotic genes in the PKT206 model that change expression between four different scenarios were calculated by comparing the steady state of the source and the target scenario. (b) The numbers of genes that prevent cellular senescence in the PKT206 model and change expression between four different scenarios were calculated by comparing the steady state of the source and the target scenario.

(a)

| Source scenario | Target scenario | Total number of genes | Number of genes up-regulated | Number of genes not changed | Number of genes down-regulated |
| --- | --- | --- | --- | --- | --- |
| P53 wild type with DNA damage | P53 mutant with DNA damage | 39 | 7 (18%)  (BCL3;PDGFRB; WWP1;IGF1R; PRSS50;EPHB4; CKS2) | 24 (62%)  (GSTP1;ESR1;  IL6;FGF2;EGFR;  CD44;PRKCA;  TGFA;CDC25A;  FOS;EZH2;PSEN1;  BCL2;CCNG1;  TCF7L2;CDKN1B;  ATF3;PTGS2;SGK;  MCL1;NOTCH1;  VEGFA;CDKN1A;  AR) | 8 (20%)  (DUSP4;DUSP2;  DDIT4;MAP4K4  FHL2;IER3;  C12orf5;SFN) |
| P53 wild type without DNA damage | P53 mutant without DNA damage | 39 | 7 (18%)  (BCL3;PDGFRB; WWP1;IGF1R; PRSS50;EPHB4; CKS2) | 24 (62%)  (GSTP1;ESR1;  IL6;FGF2;EGFR;  CD44;PRKCA;  TGFA;CDC25A;  FOS;EZH2;PSEN1;  BCL2;CCNG1;  TCF7L2;CDKN1B;  ATF3;PTGS2;SGK;  MCL1;NOTCH1;  VEGFA;CDKN1A;  AR) | 8 (20%)  (DUSP4;DUSP2;  DDIT4;MAP4K4  FHL2;IER3;  C12orf5;SFN) |
| P53 wild type without DNA damage | P53 wild type with DNA damage | 39 | 0 (0%) | 38 (97%)  (BCL3;GSTP1;  ESR1;DUSP4;  IL6;PDGFRB;  WWP1;IGF1R;  EGFR;CD44;  PRKCA;DUSP2;  TGFA;CDC25A;  FOS;DDIT4;  MAP4K4;EZH2;  PSEN1;PRSS50;  BCL2;FHL2;  CCNG1;TCF7L2;  EPHB4;CDKN1B;  ATF3;PTGS2;  SGK;MCL1;  IER3;NOTCH1;  VEGFA;CDKN1A;  AR;CKS2;  C12orf5;SFN) | 1 (3%)  (FGF2) |
| P53 mutant without DNA damage | P53 mutant with DNA damage | 39 | 0 (0%) | 38 (97%)  (BCL3;GSTP1;  ESR1;DUSP4;  IL6;PDGFRB;  WWP1;IGF1R;  EGFR;CD44;  PRKCA;DUSP2;  TGFA;CDC25A;  FOS;DDIT4;  MAP4K4;EZH2;  PSEN1;PRSS50;  BCL2;FHL2;  CCNG1;TCF7L2;  EPHB4;CDKN1B;  ATF3;PTGS2;  SGK;MCL1;  IER3;NOTCH1;  VEGFA;CDKN1A;  AR;CKS2;  C12orf5;SFN) | 1 (3%)  (FGF2) |

(b)

| Source scenario | Target scenario | Total number of genes | Number of genes up-regulated | Number of genes not changed | Number of genes down-regulated |
| --- | --- | --- | --- | --- | --- |
| P53 wild type with DNA damage | P53 mutant with DNA damage | 33 | 2  (CDK4;  FGF2) | 31  (GAPDH;  MSH2;  RRM2B;  ARID3A;  WWP1;  EGFR;  CKB;  DDIT4;  EZH2;  BCL2;  PCBP4;  HIF1A;  CD59;  FOXM1;  HNF4A;  FHL2;  BRCA1;  APAF1;  BAK1;  SGK;  S100A6;  MCL1;  GADD45A;  PTEN;  VEGFA;  DKK1;  TFDP1;  NME1;  MYC;  SESN2;  PPM1D) | 0 |
| P53 wild type without DNA damage | P53 mutant without DNA damage | 33 | 2  (CDK4;  FGF2) | 31  (GAPDH;  MSH2;  RRM2B;  ARID3A;  WWP1;  EGFR;  CKB;  DDIT4;  EZH2;  BCL2;  PCBP4;  HIF1A;  CD59;  FOXM1;  HNF4A;  FHL2;  BRCA1;  APAF1;  BAK1;  SGK;  S100A6;  MCL1;  GADD45A;  PTEN;  VEGFA;  DKK1;  TFDP1;  NME1;  MYC;  SESN2;  PPM1D) | 0 |
| P53 wild type without DNA damage | P53 wild type with DNA damage | 33 | 7  (CDK4;  FGF2;  WWP1;  FOXM1;  HNF4A;  S100A6;  TFDP1) | 14  (MSH2;  ARID3A; EGFR;  CKB;  EZH2;  BCL2;  HIF1A;  BRCA1;  APAF1;  SGK;  MCL1;  VEGFA;  NME1;  MYC) | 12  (GAPDH;  RRM2B;  DDIT4;  PCBP4;  CD59;  FHL2;  BAK1;  GADD45A;  PTEN;  DKK1;  SESN2;  PPM1D) |
| P53 mutant without DNA damage | P53 mutant with DNA damage | 33 | 7  (CDK4;  FGF2;  WWP1;  FOXM1;  HNF4A;  S100A6;  TFDP1) | 14  (MSH2;  ARID3A; EGFR;  CKB;  EZH2;  BCL2;  HIF1A;  BRCA1;  APAF1;  SGK;  MCL1;  VEGFA;  NME1;  MYC) | 12  (GAPDH;  RRM2B;  DDIT4;  PCBP4;  CD59;  FHL2;  BAK1;  GADD45A;  PTEN;  DKK1;  SESN2;  PPM1D) |

**Table S10: Number of pro-apoptotic genes and genes that promote cellular senescence with altered expression depending on p53 and DNA damage**

(a) The numbers of pro-apoptotic genes in the PKT206 model that change expression between four different scenarios were calculated by comparing the steady state of the source and the target scenario. (b) The numbers of genes that promote cellular senescence in the PKT206 model and change expression between four different scenarios were calculated by comparing the steady state of the source and the target scenario.

(a)

| Source scenario | Target scenario | Total number of genes | Number of genes up-regulated | Number of genes not changed | Number of genes down-regulated |
| --- | --- | --- | --- | --- | --- |
| P53 wild type with DNA damage | P53 mutant with DNA damage | 56 | 5 (9%)  (ECT2;XAF1;  IFITM2;HNF4A;  CDKN2A) | 24 (43%)  (ESR1;MSH2;  CXCR4;IL6;  FGF2;EGFR;  CD44;PRKCA;  BAX;CDC25A;  FOS;SIVA1;  CCNG1;CDKN1B;  APAF1;ATF3;  PTGS2;NOTCH1;  FAS;AR;  TNFRSF10A;  TNFRSF10B;  SIAH1;p53AIP1) | 27 (48%)  (DUSP4;DUSP2;  FDXR;IFI16;  TLR3;DDIT4;  MAP4K4;SEMA3B;  PEG3;PCBP4;  DFNA5;TP53INP1;  CASP8;COL18A1;  BBC3;BAK1;  NLRC4;GADD45A;  PTEN;AIFM2;  DKK1;BNIP3L;  IGFBP7;LATS2;  SERPINB5;  PERP;LRDD) |
| P53 wild type without DNA damage | P53 mutant without DNA damage | 56 | 5 (9%)  (ECT2;XAF1;  IFITM2;HNF4A;  CDKN2A) | 22 (39%)  (ESR1;MSH2;  CXCR4;IL6;  FGF2;EGFR;  CD44;PRKCA;  BAX;CDC25A;  FOS;SIVA1;  CCNG1;CDKN1B;  APAF1;ATF3;  PTGS2;NOTCH1;  AR;TNFRSF10A;  TNFRSF10B;  SIAH1) | 29 (52%)  (DUSP4;DUSP2;  FDXR;IFI16;  TLR3;DDIT4;  MAP4K4;SEMA3B;  PEG3;PCBP4;  DFNA5;TP53INP1;  CASP8;COL18A1;  BBC3;BAK1;  NLRC4;GADD45A;  FAS;PTEN;  AIFM2;DKK1;  BNIP3L;IGFBP7;  LATS2;SERPINB5;  PERP;LRDD;  p53AIP1) |
| P53 wild type without DNA damage | P53 wild type with DNA damage | 56 | 0 (0%) | 55 (98%)  (ESR1;ECT2;  MSH2;DUSP4;  CXCR4;XAF1;  IL6;EGFR;  CD44;PRKCA;  DUSP2;FDXR;  BAX;IFI16;  TLR3;CDC25A;  FOS;DDIT4;  MAP4K4;SEMA3B;  PEG3;IFITM2;  SIVA1;PCBP4;  DFNA5;HNF4A;  TP53INP1;CCNG1;  CASP8;CDKN1B;  COL18A1;BBC3;  APAF1;BAK1;  NLRC4;ATF3;  PTGS2;GADD45A;  NOTCH1;FAS;  PTEN;AIFM2;  DKK1;AR;  BNIP3L;  TNFRSF10A;  TNFRSF10B;  IGFBP7;  LATS2;SERPINB5;  PERP;SIAH1;  LRDD;p53AIP1;  CDKN2A) | 1 (2%)  (FGF2) |
| P53 mutant without DNA damage | P53 mutant with DNA damage | 56 | 2 (4%)  (FAS  p53AIP1) | 53 (94%)  (ESR1;ECT2;  MSH2;DUSP4;  CXCR4;XAF1;  IL6;EGFR;  CD44;PRKCA;  DUSP2;FDXR;  BAX;IFI16;  TLR3;CDC25A;  FOS;DDIT4;  MAP4K4;SEMA3B;  PEG3;IFITM2;  SIVA1;PCBP4;  DFNA5;HNF4A;  TP53INP1;CCNG1;  CASP8;CDKN1B;  COL18A1;BBC3;  APAF1;BAK1;  NLRC4;ATF3;  PTGS2;GADD45A;  NOTCH1;PTEN;  AIFM2;DKK1;  AR;BNIP3L;  TNFRSF10A;  TNFRSF10B;  IGFBP7;LATS2;  SERPINB5;PERP;  SIAH1;LRDD;  CDKN2A) | 1 (2%)  (FGF2) |

(b)

| Source scenario | Target scenario | Total number of genes | Number of genes up-regulated | Number of genes not changed | Number of genes down-regulated |
| --- | --- | --- | --- | --- | --- |
| P53 wild type with DNA damage | P53 mutant with DNA damage | 28 | 0 | 28  (GAPDH;  MSH2;  DDB2;  IL6;  IGF1R;  CDKN1A;  PRKCA;  TGFA;  IFI16;  PSEN1;  IFITM2;  BCL2;  CD59;  CDKN1B;  BAK1;  PTGS2;  GADD45A;  NOTCH1;  PTEN;  DKK1;  AR;  MYC;  IGFBP7;  LATS2;  RAS;  C12orf5;  SFN;  CDKN2A) | 0 |
| P53 wild type without DNA damage | P53 mutant without DNA damage | 28 | 0 | 28  (GAPDH;  MSH2;  DDB2;  IL6;  IGF1R;  CDKN1A;  PRKCA;  TGFA;  IFI16;  PSEN1;  IFITM2;  BCL2;  CD59;  CDKN1B;  BAK1;  PTGS2;  GADD45A;  NOTCH1;  PTEN;  DKK1;  AR;  MYC;  IGFBP7;  LATS2;  RAS;  C12orf5;  SFN;  CDKN2A) | 0 |
| P53 wild type without DNA damage | P53 wild type with DNA damage | 28 | 3  (IGF1R;  IFITM2;  CDKN2A) | 13  (MSH2;  IL6;  CDKN1A;  PRKCA;  TGFA;  PSEN1;  BCL2;  CDKN1B;  PTGS2;  NOTCH1  AR;  MYC;  RAS;  CDKN2A) | 12  (GAPDH;  DDB2;  IFI16;  CD59;  BAK1;  GADD45A;  PTEN;  DKK1;  IGFBP7;  LATS2;  C12orf5;  SFN) |
| P53 mutant without DNA damage | P53 mutant with DNA damage | 28 | 3  (IGF1R;  IFITM2;  CDKN2A) | 13  (MSH2;  IL6;  CDKN1A;  PRKCA;  TGFA;  PSEN1;  BCL2;  CDKN1B;  PTGS2;  NOTCH1  AR;  MYC;  RAS;  CDKN2A) | 12  (GAPDH;  DDB2;  IFI16;  CD59;  BAK1;  GADD45A;  PTEN;  DKK1;  IGFBP7;  LATS2;  C12orf5;  SFN) |

**Table S11: Number of pro- and anti-apoptotic genes with altered expression depending on p53 and DNA damage in human osteosarcoma and colon cancer cell lines.**

(a) All genes from the PKT206 model were identified in microarray analysis of U2OS (p53 positive), SAOS2 (p53 negative) human osteosarcome (200 genes from the model were analysed) and HCT116 (p53 positive and negative) human colon cancer cell lines (169 genes from the model were analysed). (b) Pro-apoptotic genes from the PKT206 model identified in microarray analysis. (c) Anti-apoptotic genes from the PKT206 model identified in microarray analysis. Results were obtained by comparing the steady state of the source and target scenario. (d) Genes that prevent cellular senescence from the PKT206 model identified in microarray analysis. (e) Genes that promote cellular senescence from the PKT206 model identified in microarray analysis. Results were obtained by comparing the steady state of the source and target scenario.

(a)

| Experiment source condition | Experiment target condition | Total number of genes | Number of up-regulated genes | Number of unchanged genes | Number of down-regulated genes |
| --- | --- | --- | --- | --- | --- |
| U2OS cells under DNA damage | SaOS2 cells under DNA damage | 200 | 19 (10%) | 161 (80%) | 20 (10%) |
| U2OS cells without DNA damage | SaOS2 cells without DNA damage | 200 | 19 (10%) | 164 (81%) | 17 (9%) |
| U2OS cells without DNA damage | U2OS cells under DNA damage | 200 | 25 (13%) | 153 (76%) | 22 (11%) |
| SaOS2 cells without DNA damage | SaOS2 cells under DNA damage | 200 | 27 (14%) | 145 (72%) | 28 (14%) |
| HCT116 cells p53+/+ without DNA damage | HCT116 cells p53-/- without DNA damage | 169 | 19 (11%) | 125 (74%) | 25 (15%) |

(b)

| Experiment source condition | Experiment target condition | Total number of pro-apoptotic genes | Number of pro-apoptotic genes up-regulated | Number of pro-apoptotic genes not changed | Number of pro-apoptotic genes down-regulated |
| --- | --- | --- | --- | --- | --- |
| U2OS cells under DNA damage | SaOS2 cells under DNA damage | 57 | 9  (DDIT4;  SEMA3B  IFITM2  COL18A1  DKK1  AR;  TNFRSF10A  IGFBP7  CDKN2A) | 38  (ESR1  ECT2  MSH2  DUSP4  CXCR4  XAF1  IL6  EGFR  PRKCA  BAX  IFI16  TLR3  CDC25A  FOS  MAP4K4  SIVA1  PCBP4  HNF4A  TP53INP1  CCNG1  CASP8  CDKN1B  BBC3  APAF1  BAK1  NLRC4  ATF3  PTGS2  GADD45A  NOTCH1  PTEN  AIFM2  BNIP3L  LATS2  SERPINB5  PERP  SIAH1  LRDD) | 8  (FGF2  CD44  DUSP2  FDXR  PEG3  DFNA5  FAS  TNFRSF10B) |
| U2OS cells without DNA damage | SaOS2 cells without DNA damage | 55 | 7  (SEMA3B  IFITM2  COL18A1  DKK1  TNFRSF10A  IGFBP7  CDKN2A) | 41  (ESR1  ECT2  MSH2  DUSP4  CXCR4  XAF1  IL6  EGFR  PRKCA  BAX  IFI16  TLR3  CDC25A  FOS  DDIT4  MAP4K4  SIVA1  PCBP4  HNF4A  TP53INP1  CCNG1  CASP8  CDKN1B  BBC3  APAF1  BAK1  NLRC4  ATF3  PTGS2  GADD45A  NOTCH1  FAS  PTEN  AIFM2  AR  BNIP3L  LATS2  SERPINB5  PERP  SIAH1  LRDD) | 7  (FGF2  CD44  DUSP2  FDXR  PEG3  DFNA5  TNFRSF10B) |
| U2OS cells without DNA damage | U2OS cells under DNA damage | 55 | 11  (IL6  CD44  DUSP2  FDXR  BAX  TLR3  FOS  ATF3  GADD45A  FAS  LRDD) | 37  (ESR1  MSH2  DUSP4  CXCR4  XAF1  FGF2  EGFR  PRKCA  IFI16  CDC25A  MAP4K4  SEMA3B  IFITM2  SIVA1  PCBP4  DFNA5  HNF4A  TP53INP1  CCNG1  CASP8  COL18A1  BBC3  APAF1  BAK1  NLRC4  PTGS2  NOTCH1  PTEN  AIFM2  DKK1  BNIP3L  TNFRSF10A  TNFRSF10B  IGFBP7  LATS2  SERPINB5  PERP) | 7  (ECT2  DDIT4  PEG3  CDKN1B  AR  SIAH1  CDKN2A) |
| SaOS2 cells without DNA damage | SaOS2 cells under DNA damage | 55 | 11  (BAX  TLR3  CDC25A  DDIT4  SIVA1  COL18A1  BBC3  ATF3  NOTCH1  DKK1  AR) | 37  (ESR1  MSH2  CXCR4  XAF1  FGF2  EGFR  DUSP2  FDXR  IFI16  FOS  MAP4K4  SEMA3B  PEG3  IFITM2  PCBP4  DFNA5  HNF4A  CCNG1  CASP8  CDKN1B  APAF1  BAK1  NLRC4  PTGS2  GADD45A  FAS  PTEN  AIFM2  BNIP3L  TNFRSF10A  TNFRSF10B  IGFBP7  LATS2  SERPINB5  PERP  SIAH1  CDKN2A) | 7  (ECT2  DUSP4  IL6  CD44  PRKCA  TP53INP1  LRDD) |
| HCT116 cells p53+/+ without DNA damage | HCT116 cells p53-/- without DNA damage | 48 | 3  (IFI16  GADD45A  SERPINB5) | 38  (ESR1  ECT2  MSH2  DUSP4  FGF2  EGFR  PRKCA  DUSP2  BAX  CDC25A  FOS  DDIT4  MAP4K4  SEMA3B  IFITM2  PCBP4  DFNA5  HNF4A  TP53INP1  CCNG1  CASP8  CDKN1B  COL18A1  BAK1  ATF3  NOTCH1  PTEN  DKK1  AR  BNIP3L  TNFRSF10A  TNFRSF10B  IGFBP7  LATS2  PERP  SIAH1  LRDD  CDKN2A) | 7  (CXCR4  IL6  CD44  FDXR  TLR3  BBC3  APAF1) |

(c)

| Experiment source condition | Experiment target condition | Total number of anti-apoptotic genes | Number of anti-apoptotic genes up-regulated | Number of anti-apoptotic genes not changed | Number of anti-apoptotic genes down-regulated |
| --- | --- | --- | --- | --- | --- |
| U2OS cells under DNA damage | SaOS2 cells under DNA damage | 38 | 4  (IGF1R  DDIT4  AR  C12orf5) | 28  (BCL3  GSTP1  ESR1  DUSP4  IL6  PDGFRB  WWP1  EGFR  PRKCA  TGFA  CDC25A  FOS  MAP4K4  EZH2  PSEN1  PRSS50  BCL2  CCNG1  TCF7L2  EPHB4  CDKN1B  ATF3  PTGS2  MCL1  IER3  NOTCH1  VEGFA  CKS2) | 6  (FGF2  CD44  DUSP2  FHL2  CDKN1A  SFN) |
| U2OS cells without DNA damage | SaOS2 cells without DNA damage | 38 | 2  (IGF1R  C12orf5) | 31  (BCL3  GSTP1  ESR1  DUSP4  IL6  PDGFRB  WWP1  EGFR  PRKCA  TGFA  CDC25A  FOS  DDIT4  MAP4K4  EZH2  PSEN1  PRSS50  BCL2  FHL2  CCNG1  TCF7L2  EPHB4  CDKN1B  ATF3  PTGS2  MCL1  IER3  NOTCH1  VEGFA  AR  CKS2) | 5  (FGF2  CD44  DUSP2  CDKN1A  SFN) |
| U2OS cells without DNA damage | U2OS cells under DNA damage | 38 | 9  (IL6  CD44  DUSP2  FOS  FHL2  ATF3  IER3  CDKN1A  SFN) | 25  (BCL3  GSTP1  ESR1  DUSP4  PDGFRB  FGF2  WWP1  IGF1R  EGFR  PRKCA  TGFA  CDC25A  MAP4K4  EZH2  PSEN1  PRSS50  BCL2  CCNG1  EPHB4  PTGS2  MCL1  NOTCH1  VEGFA  CKS2  C12orf5) | 4  (DDIT4  TCF7L2  CDKN1B  AR) |
| SaOS2 cells without DNA damage | SaOS2 cells under DNA damage | 38 | 9  (IGF1R  CDC25A  DDIT4  BCL2  EPHB4  ATF3  NOTCH1  CDKN1A  AR) | 22  (BCL3  GSTP1  ESR1  FGF2  WWP1  EGFR  DUSP2  FOS  MAP4K4  EZH2  PSEN1  PRSS50  FHL2  CCNG1  TCF7L2  CDKN1B  PTGS2  MCL1  IER3  CKS2  C12orf5  SFN) | 7  (DUSP4  IL6  PDGFRB  CD44  PRKCA  TGFA  VEGFA) |
| HCT116 cells p53+/+ without DNA damage | HCT116 cells p53-/- without DNA damage | 36 | 5  (PDGFRB  IGF1R  TGFA  SGK  IER3) | 26  (GSTP1  ESR1  DUSP4  FGF2  WWP1  EGFR  PRKCA  DUSP2  CDC25A  FOS  DDIT4  MAP4K4  EZH2  PSEN1  BCL2  FHL2  CCNG1  EPHB4  CDKN1B  ATF3  MCL1  NOTCH1  AR  CKS2  C12orf5  SFN) | 5  (BCL3  IL6  CD44  TCF7L2  CDKN1A) |

(d)

| Experiment source condition | Experiment target condition | Total number of anti- senescence genes | Number of anti- senescence genes up-regulated | Number of anti- senescence genes not changed | Number of anti- senescence genes down-regulated |
| --- | --- | --- | --- | --- | --- |
| U2OS cells under DNA damage | SaOS2 cells under DNA damage | 32 | 2  (DDIT4;  DKK1) | 27  (GAPDH;  MSH2;  CDK4;  ARID3A;  WWP1;  EGFR;  CKB;  EZH2;  BCL2;  PCBP4;  HIF1A;  CD59;  FOXM1;  HNF4A;  BRCA1;  APAF1;  BAK1;  S100A6;  MCL1;  GADD45A;  PTEN;  VEGFA;  TFDP1;  NME1;  MYC;  SESN2;  PPM1D) | 3  (RRM2B;  FGF2;  FHL2) |
| U2OS cells without DNA damage | SaOS2 cells without DNA damage | 32 | 2  (S100A6;  DKK1) | 29  (GAPDH;  MSH2;  RRM2B;  CDK4;  ARID3A;  WWP1;  EGFR;  CKB;  DDIT4;  EZH2;  BCL2;  PCBP4;  HIF1A;  CD59;  FOXM1;  HNF4A;  FHL2;  BRCA1;  APAF1;  BAK1;  MCL1;  GADD45A;  PTEN;  VEGFA;  TFDP1;  NME1;  MYC;  SESN2;  PPM1D) | 1  (FGF2) |
| U2OS cells without DNA damage | U2OS cells under DNA damage | 32 | 4  (RRM2B;  FHL2;  GADD45A;  PPM1D) | 27  (GAPDH;  MSH2;  CDK4;  ARID3A;  FGF2;  WWP1;  EGFR;  CKB;  EZH2;  BCL2;  PCBP4;  HIF1A;  CD59;  FOXM1;  HNF4A;  BRCA1;  APAF1;  BAK1;  S100A6;  MCL1;  PTEN;  VEGFA;  DKK1;  TFDP1;  NME1;  MYC;  SESN2) | 1  (DDIT4) |
| SaOS2 cells without DNA damage | SaOS2 cells under DNA damage | 32 | 4  (DDIT4;  BCL2;  BRCA1;  DKK1) | 27  (GAPDH;  MSH2;  RRM2B;  CDK4;  ARID3A;  FGF2;  WWP1;  EGFR;  CKB;  EZH2;  PCBP4;  HIF1A;  CD59;  FOXM1;  HNF4A;  FHL2;  APAF1;  BAK1;  S100A6;  MCL1;  GADD45A;  PTEN;  TFDP1;  NME1;  MYC;  SESN2;  PPM1D) | 1  (VEGFA) |
| HCT116 cells p53+/+ without DNA damage | HCT116 cells p53-/- without DNA damage | 30 | 3  (SGK;  GADD45A;  SESN2) | 25  (MSH2;  CDK4;  ARID3A;  FGF2;  WWP1;  EGFR;  CKB;  DDIT4;  EZH2;  BCL2;  PCBP4;  HIF1A;  CD59;  FOXM1;  HNF4A;  FHL2;  BRCA1;  BAK1;  S100A6;  MCL1;  PTEN;  DKK1;  TFDP1;  MYC;  PPM1D) | 2  (RRM2B;  APAF1) |

(e)

| Experiment source condition | Experiment target condition | Total number of pro- senescence genes | Number of pro- senescence genes up-regulated | Number of pro- senescence genes not changed | Number of pro- senescence genes down-regulated |
| --- | --- | --- | --- | --- | --- |
| U2OS cells under DNA damage | SaOS2 cells under DNA damage | 28 | 7  (IGF1R;  IFITM2;  DKK1;  AR;  IGFBP7;  C12orf5;  CDKN2A) | 18  (GAPDH;  MSH2;  IL6;  PRKCA;  TGFA;  IFI16;  PSEN1;  BCL2;  CD59;  CDKN1B;  BAK1;  PTGS2;  GADD45A;  NOTCH1;  PTEN;  MYC;  LATS2;  RAS) | 3  (DDB2;  CDKN1A;  SFN) |
| U2OS cells without DNA damage | SaOS2 cells without DNA damage | 28 | 6  (IGF1R;  IFITM2;  DKK1;  IGFBP7;  C12orf5;  CDKN2A) | 19  (GAPDH;  MSH2;  IL6;  PRKCA;  TGFA;  IFI16;  PSEN1;  BCL2;  CD59;  CDKN1B;  BAK1;  PTGS2;  GADD45A;  NOTCH1;  PTEN;  AR;  MYC;  LATS2;  RAS) | 3  (DDB2;  CDKN1A;  SFN) |
| U2OS cells without DNA damage | U2OS cells under DNA damage | 28 | 5  (DDB2;  IL6;  CDKN1A;  GADD45A;  SFN) | 20  (GAPDH;  MSH2;  IGF1R;  PRKCA;  TGFA;  IFI16;  PSEN1;  IFITM2;  BCL2;  CD59;  BAK1;  PTGS2;  NOTCH1;  PTEN;  DKK1;  MYC;  IGFBP7;  LATS2;  RAS;  C12orf5) | 3  (CDKN1B;  AR;  CDKN2A) |
| SaOS2 cells without DNA damage | SaOS2 cells under DNA damage | 28 | 7  (DDB2;  IGF1R;  CDKN1A;  BCL2;  NOTCH1;  DKK1  AR) | 18  (GAPDH;  MSH2;  IFI16;  PSEN1;  IFITM2;  CD59;  CDKN1B;  BAK1;  PTGS2;  GADD45A;  PTEN;  MYC;  IGFBP7;  LATS2;  RAS;  C12orf5;  SFN;  CDKN2A) | 3  (IL6;  PRKCA;  TGFA) |
| HCT116 cells p53+/+ without DNA damage | HCT116 cells p53-/- without DNA damage | 26 | 5  (IGF1R;  TGFA;  IFI16;  GADD45A;  RAS) | 18  (MSH2;  PRKCA;  PSEN1;  IFITM2;  BCL2;  CD59;  CDKN1B;  BAK1;  NOTCH1;  PTEN;  DKK1;  AR;  MYC;  IGFBP7;  LATS2;  C12orf5;  SFN;  CDKN2A) | 3  (DDB2;  IL6;  CDKN1A) |
